# Revealing the Iron-Catalyzed β-Methyl Scission of *tert*-Butoxyl Radicals via the Mechanistic Studies of Carboazidation of Alkenes

**DOI:** 10.3390/molecules25051224

**Published:** 2020-03-09

**Authors:** Mong-Feng Chiou, Haigen Xiong, Yajun Li, Hongli Bao, Xinhao Zhang

**Affiliations:** 1 Key Laboratory of Coal to Ethylene Glycol and Its Related Technology, State Key Laboratory of Structural Chemistry, Center for Excellence in Molecular Synthesis, Fujian Institute of Research on the Structure of Matter, Chinese Academy of Sciences, 155 Yangqiao Road West, Fuzhou 350002, Fujian, China; qiumengfeng@fjirsm.ac.cn (M.-F.C.); xionghaigen@fjirsm.ac.cn (H.X.); liyajun@fjirsm.ac.cn (Y.L.); 2 School of Chemistry and Chemical Engineering of University of Chinese Academy of Sciences, Beijing 100049, China; 3 Lab of Computational Chemistry and Drug Design, Key Laboratory of Chemical Genomics, Peking University Shenzhen Graduate School, Shenzhen 518055, China

**Keywords:** iron-catalysis, carboazidation, β-methyl scission, radical, DFT

## Abstract

We describe here a mechanistic study of the iron-catalyzed carboazidation of alkenes involving an intriguing metal-assisted β-methyl scission process. Although t-BuO radical has frequently been observed in experiments, the β-methyl scission from a t-BuO radical into a methyl radical and acetone is still broadly believed to be thermodynamically spontaneous and difficult to control. An iron-catalyzed β-methyl scission of t-BuO is investigated in this work. Compared to a free t-BuO radical, the coordination at the iron atom reduces the activation energy for the scission from 9.3 to 3.9 ~ 5.2 kcal/mol. The low activation energy makes the iron-catalyzed β-methyl scission of t-BuO radicals almost an incomparably facile process and explains the selective formation of methyl radicals at low temperature in the presence of some iron catalysts. In addition, a radical relay process and an outer-sphere radical azidation process in the iron-catalyzed carboazidation of alkenes are suggested by density functional theory (DFT) calculations.

## 1. Introduction

**Scheme 1:**
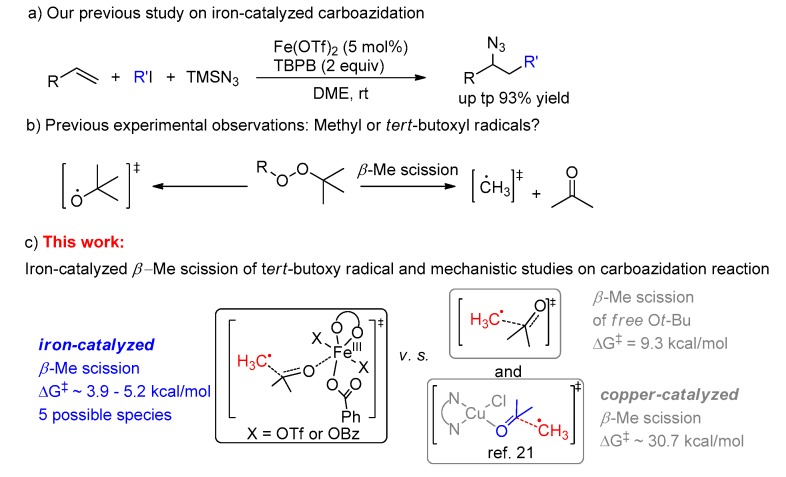
(**a**) Carboazidation of alkenes in previous study, [9] (**b**) selective formation of methyl radicals [33,34,35,36,37] or t-BuO radical, [10,11,12,13,14,15,16,17,18,19,20,21,22,23,24,25,26,27,28,29,30,31,32] and (**c**) mechanistic studies.

The carboazidation of alkenes, a powerful and promising method for the synthesis of amino acid precursors and other useful building blocks, has attracted much attention recently [1,2,3,4,5,6,7,8]. Iron-catalyzed carboazidation of alkenes has recently been developed by our group in which *tert*-butyl peroxybenzoate (TBPB) was employed as the initiator (Scheme 1a) [9].

*tert*-Butoxy-containing peroxides, including di-*tert*-butyl peroxide (DTBP), [10,11] *tert*-butyl hydroperoxide (TBHP), [12,13,14,15,16,17,18,19,20,21] and *tert*-butyl peroxybenzoate (TBPB), [22,23,24,25,26,27,28,29,30,31,32] have versatile roles in organic synthesis and have been proven to be good sources of t-BuO radical. However, these peroxides can also occasionally serve as a source of methyl radicals (Scheme 1b) [33,34,35,36,37]. The β-methyl scission of alkoxy radicals which is a common fragmentation process forming corresponding alkyl radicals was discovered more than fifty years ago [38], and is described in organic chemical textbooks [39]. The β-methyl scission from a t-BuO radical accordingly is believed to be an easily spontaneous process, [40,41,42,43,44] and offers a facile pathway to methyl radicals; however, it is inconsistent with the common experimental observation of t-BuO radical [25,26,27,28,29,30,31,32].

Although β-methyl scission from a t-BuO radical can afford methyl radical, the factors which determine the selective formation of methyl radicals or retaining as t-BuO radical are still unclear. To the best of our knowledge, no further investigation of t-BuO radical splitting has been reported. Very recently, our studies suggested that a copper catalyst may not assist the β-methyl scission (Scheme 1c, 30.7 kcal/mol) and the t-BuO radical can become untethered which serves as the radical initiator and does not proceed β-methyl scission [21]. On the other hand, the selective or dominant formation of methyl radicals in iron-catalyzed reactions has frequently been observed in our previous work [33,34,35]. It is questionable why the t-BuO radical behaves very differently when catalyzed by iron.

Herein, the crucial factors for the selective formation of methyl radicals have been investigated and a rare iron-catalyzed β-methyl scission was revealed (Scheme 1c). In addition, experimental and theoretical investigations were conducted to support a radical relay mechanism for carboazidation reactions [9]. 

## 2. Results and Discussion

**Scheme 2:**
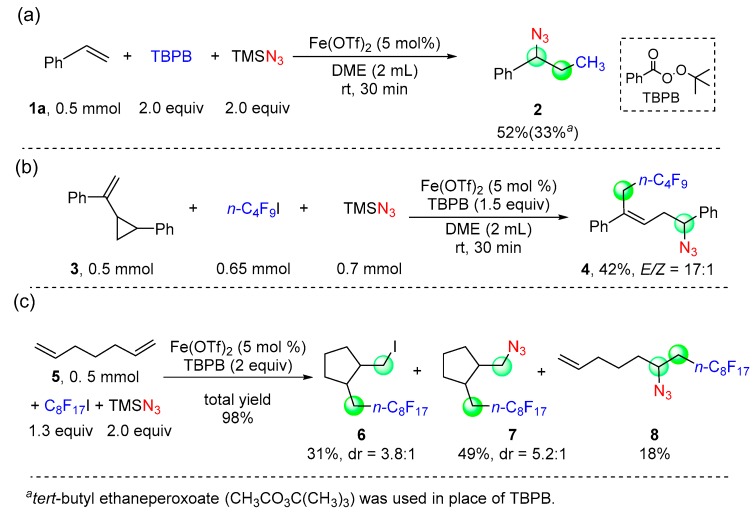
Experimental studies. (**a**) Participation of the methyl radical within the caboazidation of alkenes in the absence of further alkyl iodides, (**b**) and (**c**) Ring-opening and ring-closing experiments for exploring the radical relay mechanism.

Experiments exploring the carboazidation reactions of alkenes were conducted to probe the mechanism. First, the reaction was conducted in the absence of alkyl iodide, as expected, a methyl adduct **2**, (1-azidopropyl)benzene, was obtained in 52% yield (Scheme 2a). The reaction employing *tert*-butyl ethaneperoxoate as initiator instead of TBPB also delivers the desired product (**2**) in 33% yield demonstrating that the methyl radical can be easily generated at room temperature under these conditions. Because of the absence of diazidation product which can indirectly prove the existence of t-BuO radial [45] in all cases under standard conditions or these two conditions, the formation of methyl radical can be regarded as highly selective. Next, (2-phenylcyclopropyl)styrene (**3**), a radical clock compound, for which the rate constant of the ring opening step is approximately 10^8^ s^−1^, was used and afforded a ring-opened product (**4**) in 42% yield (Scheme 2b) [46]. A ring closure reaction was also conducted with 1,6-heptadiene **5** and the ring-closure products (**6** and **7**) and non-ring-closed product (**8**) were obtained in 80% and 18% yields, respectively (Scheme 2c). This result suggests that the azidation step is quite fast and is comparable to 5-exo-cyclization of 5-hexenyl radical (~10^5^/s). Besides, radical scavengers, 2,6-di-tert-butyl-4-methylphenol (BHT) or hydroquinone, can interrupt the standard reaction to reduce the yield of product (See Preliminary mechanistic studies in supporting information). These results are consistent with a radical mechanism [47,48].

Interestingly, the acetone and methyl iodide formed under standard conditions could be observed by GC-MS (See supporting information), implying that the radical relay process possibly begins with a methyl radical.

**Figure 1:**
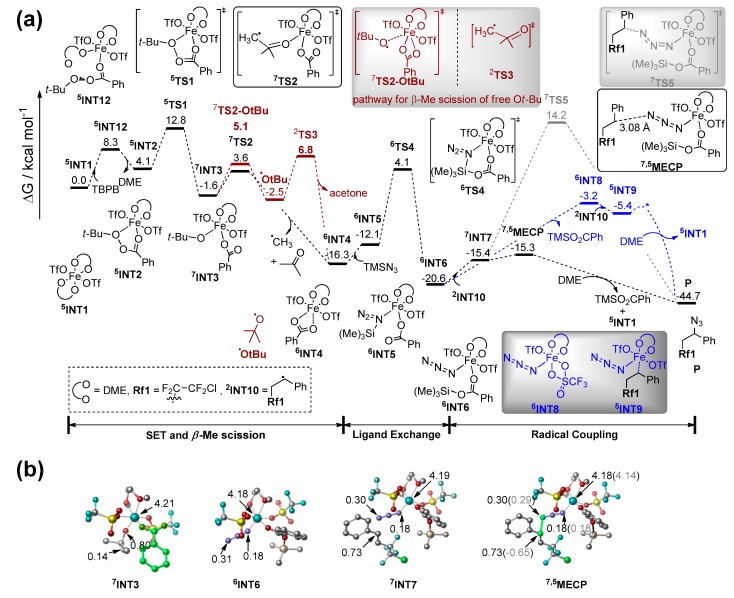
(**a**) The Gibbs free energy profile of the Fe-catalyzed carboazidation of alkenes. The transition state corresponding to reductive elimination from **^5^INT9** cannot be explicitly located and is indicated as *. (**b**) Optimized structures of selected intermediates. Spin densities on selected atoms are shown in each structure beside the arrows. For **^7,5^MECP**, the spin densities of the quintet state are shown in parenthesis. Hydrogen atoms are omitted for clarity.

Next, density functional theory (DFT) calculations on the iron-catalyzed carboazidation of styrene were performed in an attempt to understand the mechanism at the atomic level [49,50]. Figure 1a shows the overall potential energy surface of the iron-catalyzed reaction. According to the systematic computations on the spin states and the conformations of iron species, a quintet state of catalyst Fe(OTf)_2_ (**^5^INT1**) coordinated by two 1,2-dimethoxyethane (DME) molecules, was found to have the lowest free energy (Table S1), and thus can be considered as the starting catalyst for first cycle [51,52].

The interaction of TBPB with **^5^INT1** yields **^5^INT2** by an associative ligand exchange process. An associative intermediate, **^5^INT12**, with a relative free energy of 8.3 kcal/mol can be considered as a barrier to the ligand exchange (Supplementary Figure S1) [53,54]. Subsequently, a single electron transfer (SET) occurs, breaking the O-O bond of TBPB with an energy barrier of 8.7 kcal/mol and resulting in a septet, (**^7^INT3**) of the Fe(III) species coordinated by a tethered t-BuO radical with a exergonicity of 5.7 kcal/mol. As displayed in Figure 1b, the oxygen atom of the tethered t-BuO in **^7^INT3** acquires an unpaired spin density, indicating that the t-BuO moiety becomes a radical during the SET process. Since the selective formation of methyl radical was observed in this reaction and in our previous work [33], two pathways of methyl radical generation were therefore considered.

**Figure 2:**
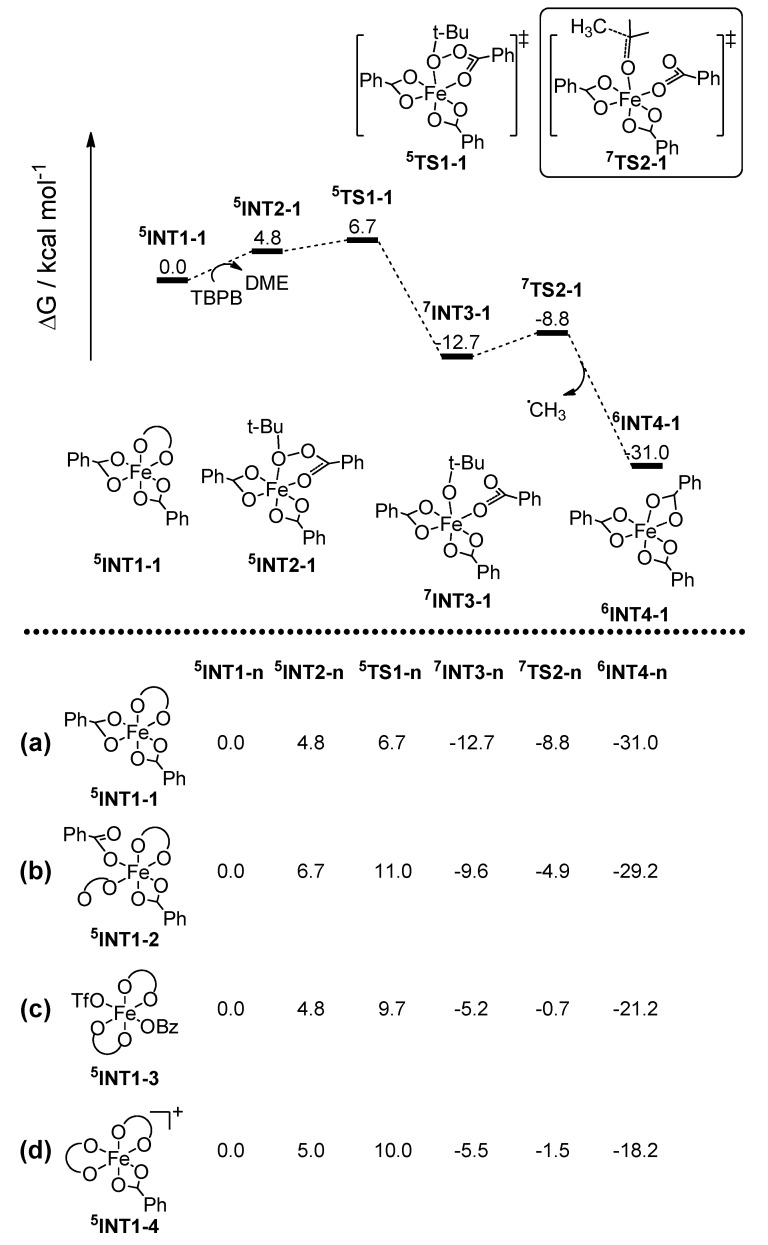
The Gibbs free energy profiles of SET and iron-catalyzed β-methyl scission processes for (**a**) Fe(OBz)_2_(DME), **^5^INT1-1**, (**b**) Fe(OBz)_2_(DME)_2_, **^5^INT1-2**, (**c**) Fe(OTf)(OBz)(DME)_2_, **^5^INT1-3** and (**d**) cation species [Fe(OBz)(DME)_2_]^+^, **^5^INT1-4**, showing that different possible Fe(II/III) species facilitate the β-scission of t-BuO radical. Relative free energies are in kcal/mol.

With a terminal carbon having a spin density in **^7^INT3** (cf. Figure 1b), a transition state **^7^TS2**, corresponding to C-C bond cleavage along this coordinate, is located with a quite low barrier, 5.2 kcal/mol, and leads to a sextet (**^6^INT4**) with a free methyl radical. Surprisingly, **^7^TS2-OtBu**, dissociation of an t-BuO radical from **^7^INT3** was found to be an unfavorable process, requiring a higher energy barrier (6.7 kcal/mol) to lead to a free t-BuO radical and a sextet **^6^INT4**, with only 0.9 kcal/mol small exergonicity (red path in Figure 1a). A much higher barrier of 9.3 kcal/mol is required (**^2^TS3**) for dissociation of a methyl radical from a free t-BuO radical [21] indicating that a free methyl radical generated directly from **^7^INT3** is thermodynamically and kinetically favorable. In addition, since there will be benzoate anions in the system following the occurrence of SET on the TBPB, several possible iron(III) species ligated by different anions with the tethered t-BuO radical were also examined for the possibility that they could assist the β-methyl scission. Figure 2 depicts the free energy profiles of SET and iron-catalysed β-methyl scission processes for four candidate iron complexes, **^5^INT1-1** to **^5^INT1-4** (Supplementary Table S3). Encouragingly, the energy barriers of SET for these species are in the range of 6.7–11.0 kcal/mol smaller than that for **^5^INT1**. Besides, the energy barriers of iron-catalyzed β-methyl scission for these species are in the range of 3.9–4.7 kcal/mol which are all smaller than that of β-methyl scission from the free t-BuO radical (9.3 kcal/mol) and even smaller than that of **^7^TS2** (5.2 kcal/mol) indicating that, in the reaction condition, these possible iron-catalyst species can also perform the SET on TBPB and assist the β-methyl scission well after initial catalytic cycle. These results are in good agreement with our experiment results in which no t-BuO radical derivative was observed due to the incomparable process of the generation of a free methyl radical. This implies that the Fe(III) catalyst may assist the β-methyl scission even at room temperature. This study offers a clear image of the whole decomposition process from TBPB to the t-BuO radical and a methyl radical.

**Figure 3:**
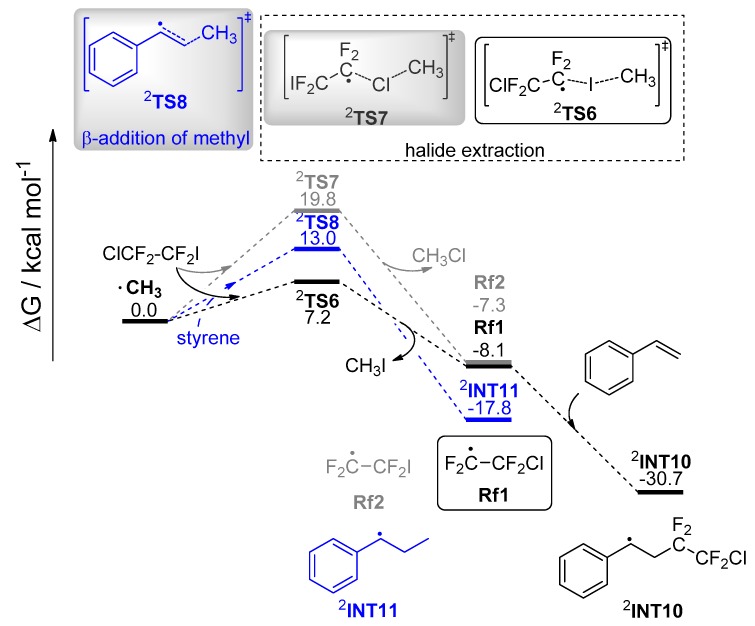
The Gibbs free energy profile for the benzyl radical generation from methyl radical via radical relay pathway. Relative free energies are in kcal/mol.

Subsequently, a radical relay starting from a free methyl radical and generating the benzyl radical **^2^INT10** was demonstrated to be a facile process and shown in Figure 3. Herein, 1-chloro-1,1,2,2-tetrafluoro-2-iodoethane is employed as a perhaloalkyl iodide model to conduct the radical relay process. Transition state, **^2^TS6**, corresponding to CH_3_I and perhaloalkyl radical **Rf1** generations is located with the lowest barrier of 7.2 kcal/mol which is much lower than that of chlorine extraction (**^2^TS7**, 19.8 kcal/mol). On the other hand, methyl β-addition to styrene [35,55] is also considered; however, a much higher barrier (13.0 kcal/mol of **^2^TS8**) is found for **^2^INT11** producing. Although the relative free energy of **^2^INT11** (−17.8 kcal/mol) is lower than that of **Rf1** (−8.1 kcal/mol), **Rf1** addition to styrene is barrierless to result in a much exergonic **^2^INT10** (−30.7 kcal/mol). Owing to the flat and long range effective (~3.22 Å) potential energy surface (PES), TS for **^2^INT10** production cannot be located, suggesting that this radical relay process should be fast (see Supplementary Figure S2).

We then focused on the iron-catalyzed azidation. Formation of **^6^INT5** by TMSN_3_ (trimethylsilyl azide) complexing with the Fe in **^6^INT4** via the internal nitrogen atom, was initially calculated. A trimethylsilyl group migration transition state (**^6^TS4**) leading to an exergonic azide complex (**^6^INT6**) was identified [8]. Charge transfer to the iron center from the azide increases gradually during trimethylsilyl group migration. In particular, spin spreads to the internal and terminal nitrogen atom, implying that the azide adopts a radical characteristic even though its net charge is negative (cf. Figure 1b). Three possible pathways leading to the C-N bond coupling via a septet state, a quintet state or a septet-quintet crossing, were considered. In the septet state, the reaction pathway in which the benzyl radical, **^2^INT10**, couples directly with the terminal nitrogen atom of the azide in **^6^INT6** was considered due to its larger spin density and the reduced steric hindrance. An outer-sphere azide reaction has also been proposed in Mn catalysis [56], but the transition state **^7^TS5** has an extremely high barrier of 34.8 kcal/mol.

The inner-sphere pathway via an intermediate with an iron-carbon bond was also considered [57]. A quintet, **^5^INT9**, formed through the TMSOBz-dissociated sextet **^6^INT8**, was found to have a much higher relative free energy (−5.4 kcal/mol), suggesting that this pathway involving an intermediate containing a newly formed Fe-C bond, leading to the dissociation of the TMSOBz and association of **^2^INT10** to **^6^INT6**, is unfavorable. This result suggests that the oxidation of a Fe(III) by a benzyl radical to form Fe(IV) is an unfavorable pathway in this reaction and this finding is consistent with Gutierrez’s study although they investigated different iron species [58].

We then sought a minimum energy crossing point (MECP) crossing between the septet and the quintet states. Intermediate **^7^INT7**, approaching an **^2^INT10** to **^6^INT6**, was located with the relative free energy only 5.2 kcal/mol higher than that of **^6^INT6**, and the MECP was found at a distance *d*(C_b_-N_t_), between carbon and the terminal nitrogen atom of 3.08 Å (cf. 3.23 Å in **^7^INT7**, Figure 1b). The electronic energy of the MECP was estimated to be slightly higher (~0.1 kcal/mol) than that of **^7^INT7** [59]. After spin state crossing, no transition state relevant to product formation can be located due to the flat potential energy surface corresponding to *d*(Fe-N_i_) elongation around *d*(C_b_-N_t_) of *ca.* 3.08 Å (Figure S3). The potential energy surface corresponding to *d*(Fe-N_i_) elongation accompanying the *d*(C_b_-N_t_) shortening shows no barrier and can proceed downhill to product formation. This result is analogous to the halogenations of carbon centered radicals with iron(III)-halide species, [60,61] and suggests that only 5.2~5.3 kcal/mol is required to conduct spin state crossing, after which product formation is spontaneous.

A septet intermediate, **^7^INT9**, has been calculated with the energy of 4.8 kcal/mol higher than **^5^INT9**, (see Supplementary Table S4). A transition state between **^6^INT8** and **^5^INT9** for inner-sphere radical coupling may exist but cannot be located. On the other hand, much effort was made to locate the transition state after the inner-sphere spin crossing point, but was unsuccessful. This result may be regarded as a barrierless reductive elimination for a high-valent metal complex, [62,63] but the absence of a transition state for the inner-sphere pathway does not affect the conclusion of a favorable outer-sphere pathway since the free energy of **^5^INT9** is much higher than that of **^7^INT7**. Moreover, such azidation processes with another possible Fe(III)N_3_ species, **^6^INT6-1**, has also been calculated, and as expected, the outer-sphere pathway remains the favorable route (see Supplementary Figure S4), indicating that the catalytic cycle can perform after first cycle as well as the Fe(III)N_3_ species with OTf^-^ anion.

**Scheme 3:**
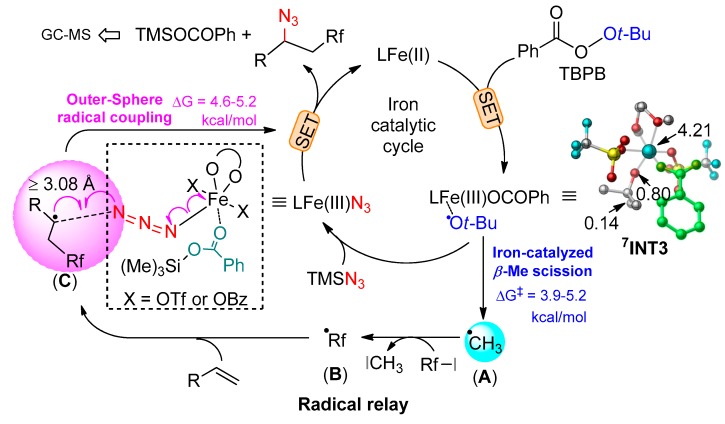
Proposed mechanism for carboazidation of alkenes. SET, single electron transfer. Optimized structure, **^7^INT3**, are depicted as in Figure 1b.

In view of the results of these mechanistic studies, a radical relay-involved catalytic cycle is proposed and is shown in Scheme 3. A SET between iron catalyst and TBPB initiates the reaction by generating a methyl radical (**A**), an Fe(III) species and acetone. A radical relay process then occurs between the methyl radical and the alkyl iodide affording a new carbon radical (**B**) and methyl iodide. This carbon radical adds to the olefin, generating an internal radical (**C**). Azidotrimethylsilane as a ligand delivers an Fe(III)N_3_ species, [8] which ultimately reacts with the radical (**C**) to deliver the desired alkylazidation products, regenerating Fe(II) [ (**C**) + Fe(III)N_3_ → (**C**)-N_3_ + Fe(II) ]. The C-N_3_ bond formation from alkenes can be facilitated by the Fe(III)N_3_ species as well as by the putative Mn(III) species [64]. According to the theoretical study, an iron-catalyzed β-methyl scission is an incomparable process for generation of the initial methyl radical; in addition, an outer-sphere radical coupling pathway [56,65,66] is thought to be the more favorable pathway. Similar outer-sphere radical capture for direct C-N bond formation have been reported on the C‑H amination of copper(II) anilides [67,68,69].

## 3. Materials and Methods

### 3.1. Experimental Section

#### 3.1.1. General Information

All reactions were carried out under an atmosphere of nitrogen in dried glassware with magnetic stirring unless otherwise indicated. Compound **3** in Scheme 2 was synthesized in our lab and other chemicals obtained from commercial suppliers were used without further purification. The purity of iron catalyst between different vendors (Energy Chemical, Bokachem and ^®^HEOWNS) did not change the yields of products when other batches of iron triflate were purchased. Solvents were dried by Innovative Technology Solvent Purification System. Liquids and solutions were transferred via syringe. All reactions were monitored by thin-layer chromatography. GC and GC-MS data were recorded on Thermo Trace 1300 (Thermo Fisher Scientific, Milan, Italy) and Thermo ISQ QD, respectively. ^1^H-, ^19^F-, and ^13^C-NMR spectra were recorded on Bruker-BioSpin AVANCE III HD-400 Hz (Bruker BioSpin GmbH, Rheinstetten, Germany). Data for ^1^H-NMR spectra are reported relative to chloroform as an internal standard (7.26 ppm) and are reported as follows: chemical shift (ppm), multiplicity, coupling constant (Hz), and integration. Data for ^13^C-NMR spectra were reported relative to chloroform as an internal standard (77.00 ppm) and are reported in terms of chemical shift (ppm). IR data were obtained from Bruker VERTEX 70. All melting points were determined on a Beijing Science Instrument Dianguang Instrument (Beijing, China) Factory XT4B melting point apparatus and are uncorrected. HRMS(ESI) data were recorded on Agilent Technologies 6224 TOF LC/MS (Agilent, Palo Alto, CA, USA); HRMS(EI) data were recorded on Waters Micromass GCT Premier (Waters, MMAS, New York, NY, USA).

#### 3.1.2. General Procedure for Confirmation of Methyl Radical

To a dried Schlenk tube equipped with a magnetic bar, Fe(OTf)_2_ (9 mg, 0.025 mmol) was added, flushed with nitrogen gas (3 times) and maintained the nitrogen atmosphere using the balloon. A thoroughly mixed solution of vinylarene (0.5 mmol), TMSN_3_ (1.0 mmol) and TBPB (or *tert*-butyl ethaneperoxoate) (1.0 mmol) in DME (2 mL) was added to the catalyst via syringe and stirred vigorously for 30 min at room temperature. The solvent was evaporated, and the residue was purified by flash chromatography on silica gel to give the corresponding product **9** in 52% (33%) yield.

### 3.2. Computational Method and Details

Density functional theory (DFT) studies on the iron-catalyzed carboazidation of styrene were performed at B3LYP [70,71] -D3 [72,73] /Def2-SVP [74,75] level of theory in gas-phase for geometrical optimizations, thermal energy calculations, and frequency analyses. Transition state structures were searched by simply performing a crude relaxed potential energy surface (RPES) scan connecting reactants and products, and then optimized by the three-structure synchronous transit-guided quasi-Newton (STQN) method, [76,77] and rational function optimization (RFO) method of TS as well [78]. In addition, transition state vibrational frequencies were verified to have one and only one imaginary frequency and confirm the correctness of the imaginary frequency by viewing normal mode vibrational vector. All optimized stationary points were characterized by frequency calculation for identification of minimum points and saddle points. Single point energies based upon the optimized structures were calculated at the B3LYP-D3/Def2-TZVP [74,75] level of theory with SMD solvation model calculation in DME solution, [79] and the reported Gibbs free energy is obtained by adding the solution-phase electronic energy with the gas-phase Gibbs free energy correction for saving the computational time consumption. To verify the reliability of the geometries and thermal corrections obtained in the gas phase, geometrical optimizations as well as the frequency calculations for **^5^INT1**, TBPB, DME, **^5^INT2**, **^5^TS1,** and **^7^INT3** were also carried out with SMD solvation model. Figure S5 depicts the free energy profile of pathway from **^5^INT1** to **^7^INT3** in which the gas-phase thermal energy correction shows well comparative to the solvation thermal energy correction. Other functionals including types of generalized gradient approximation (GGA), meta-GGA and hybrid functional including dispersion were also employed for **^5^INT1** (i.e., Fe(OTf)_2_(DME)_2_) optimization on quintet, triplet and singlet spin states to confirm the validity of quintet state. Supplementary Table S2 shows the similar energetic tendency supporting that employing the quintet state **^5^INT1** to initiate studies should be reliable. On the other hand, for radical coupling, minimum energy crossing point (MECP) was also located by using the hybrid approach method of Harvey [80]. All calculations were performed by the Gaussian 09 package (Gaussian, Wallingford, CT, USA) [81].

## 4. Conclusions

In summary, experimental studies have established the selective formation of methyl radical formation for this iron-catalyzed carboazidation of alkenes. The methyl radical can be identified by GC-MS and be found in the product in the absence of further alkyl iodides indicating that a methyl radical is easily propagated at room temperature under the reaction conditions. Theoretical studies reveal that the methyl radical propagation via β-Me scission of the t-BuO radical will be assisted by the iron catalysis. The energy barrier of methyl radical release from a coordinated t-BuO radical is far lower than that of untethered one. In addition, the formed methyl radical has a lower barrier to abstract an iodine atom from the alkyl iodide instead of reacting with the styrene which explains the outcome of products through the radical relay process. In the end, the iron-catalyzed carboazidation of alkenes may undergo an outer-sphere radical coupling via an Fe(III)N_3_ intermediate to form products. This study may shed some light on the metal-catalyzed SET reactions of peroxides and may offer a partial explanation of the formation of methyl radical [21,36,37,44].

## Supplementary Materials

The following are available online, Supporting Information including all NMR spectroscopic analysis, characterization data, GC-MS analysis, Figure S1–S5, Tables S1–S4 and Cartesian coordinates of all optimized structures.

## References

[1] Huang W.-Y., Lü L. (1992). The reaction of perfluoroalkanesulfinates VII. Fenton reagent-initiated addition of sodium perfluoroalkanesulfinates to alkenes. Chin. J. Chem..

[2] Renaud P., Ollivier C., Panchaud P. (2002). Radical Carboazidation of Alkenes: An Efficient Tool for the Preparation of Pyrrolidinone Derivatives. Angew. Chem. Int. Ed..

[3] Weidner K., Giroult A., Panchaud P., Renaud P. (2010). Efficient carboazidation of alkenes using a radical desulfonylative azide transfer process. J. Am. Chem. Soc..

[4] Wang F., Qi X., Liang Z., Chen P., Liu G. (2014). Copper-Catalyzed Intermolecular Trifluoromethylazidation of Alkenes: Convenient Access to CF3-Containing Alkyl Azides. Angew. Chem. Int. Ed..

[5] Dagousset G., Carboni A., Magnier E., Masson G. (2014). Photoredox-induced three-component azido- and aminotrifluoromethylation of alkenes. Org. Lett..

[6] Bunescu A., Ha T.M., Wang Q., Zhu J. (2017). Copper-Catalyzed Three-Component Carboazidation of Alkenes with Acetonitrile and Sodium Azide. Angew. Chem. Int. Ed..

[7] Geng X., Lin F., Wang X., Jiao N. (2017). Azidofluoroalkylation of Alkenes with Simple Fluoroalkyl Iodides Enabled by Photoredox Catalysis. Org. Lett..

[8] Zhu C.-L., Wang C., Qin Q.-X., Yruegas S., Martin C.D., Xu H. (2018). Iron(II)-Catalyzed Azidotrifluoromethylation of Olefins and N-Heterocycles for Expedient Vicinal Trifluoromethyl Amine Synthesis. ACS Catal..

[9] Xiong H., Ramkumar N., Chiou M.-F., Jian W., Li Y., Su J.-H., Zhang X., Bao H. (2019). Iron-catalyzed carboazidation of alkenes and alkynes. Nat. Commun..

[10] Li Z., Cao L., Li C.-J. (2007). FeCl2-Catalyzed Selective C–C Bond Formation by Oxidative Activation of a Benzylic C–H Bond. Angew. Chem. Int. Ed..

[11] Lv L., Lu S., Guo Q., Shen B., Li Z. (2015). Iron-Catalyzed Acylation-Oxygenation of Terminal Alkenes for the Synthesis of Dihydrofurans Bearing a Quaternary Carbon. J. Org. Chem..

[12] Pan S., Liu J., Li H., Wang Z., Guo X., Li Z. (2010). Iron-Catalyzed N-Alkylation of Azoles via Oxidation of C−H Bond Adjacent to an Oxygen Atom. Org. Lett..

[13] Liu W., Li Y., Liu K., Li Z. (2011). Iron-Catalyzed Carbonylation-Peroxidation of Alkenes with Aldehydes and Hydroperoxides. J. Am. Chem. Soc..

[14] Boess E., Schmitz C., Klussmann M. (2012). A Comparative Mechanistic Study of Cu-Catalyzed Oxidative Coupling Reactions with N-Phenyltetrahydroisoquinoline. J. Am. Chem. Soc..

[15] Leifert D., Daniliuc C.G., Studer A. (2013). 6-Aroylated phenanthridines via base promoted homolytic aromatic substitution (BHAS). Org. Lett..

[16] Wang J., Liu C., Yuan J., Lei A. (2013). Copper-Catalyzed Oxidative Coupling of Alkenes with Aldehydes: Direct Access to α,β-Unsaturated Ketones. Angew. Chem. Int. Ed..

[17] Wei W.-T., Zhou M.-B., Fan J.-H., Liu W., Song R.-J., Liu Y., Hu M., Xie P., Li J.-H. (2013). Synthesis of Oxindoles by Iron-Catalyzed Oxidative 1,2-Alkylarylation of Activated Alkenes with an Aryl C(sp2)–H Bond and a C(sp3)–H Bond Adjacent to a Heteroatom. Angew. Chem. Int. Ed..

[18] Zhou M.-B., Song R.-J., Ouyang X.-H., Liu Y., Wei W.-T., Deng G.-B., Li J.-H. (2013). Metal-free oxidative tandem coupling of activated alkenes with carbonyl C(sp2)–H bonds and aryl C(sp2)–H bonds using TBHP. Chem. Sci..

[19] Wei W.-T., Song R.-J., Li J.-H. (2014). Copper-Catalyzed Oxidative α-Alkylation of α-Amino Carbonyl Compounds with Ethers via Dual C(sp3)-H Oxidative Cross- Coupling. Adv. Synth. Catal..

[20] Zhao J., Li P., Xia C., Li F. (2014). Direct N-acylation of azoles via a metal-free catalyzed oxidative cross-coupling strategy. Chem. Commun..

[21] Jiao Y., Chiou M.-F., Li Y., Bao H. (2019). Copper-Catalyzed Radical Acyl-Cyanation of Alkenes with Mechanistic Studies on the tert-Butoxy Radical. ACS Catal..

[22] Kharasch M.S., Sosnovsky G. (1958). The Reactions of *t*-Butyl Perbenzoate and Olefins—A Stereospecific Reaction. J. Am. Chem. Soc..

[23] Kochi J.K., Mains H.E. (1965). Studies on the Mechanism of the Reaction of Peroxides and Alkenes with Copper Salts. J. Org. Chem..

[24] Pryor W.A., Hendrickson W.H. (1983). Reaction of nucleophiles with electron acceptors by SN2 or electron transfer (ET) mechanisms: Tert-butyl peroxybenzoate/dimethyl sulfide and benzoyl peroxide/N,N-dimethylaniline systems. J. Am. Chem. Soc..

[25] Sekar G., DattaGupta A., Singh V.K. (1998). Asymmetric Kharasch Reaction:  Catalytic Enantioselective Allylic Oxidation of Olefins Using Chiral Pyridine Bis(diphenyloxazoline)−Copper Complexes and tert-Butyl Perbenzoate. J. Org. Chem..

[26] Zhou S.-L., Guo L.-N., Wang H., Duan X.-H. (2013). Copper-Catalyzed Oxidative Benzylarylation of Acrylamides by Benzylic C–H Bond Functionalization for the Synthesis of Oxindoles. Chem. Eur. J..

[27] Cai Z.-J., Lu X.-M., Zi Y., Yang C., Shen L.-J., Li J., Wang S.-Y., Ji S.-J. (2014). I_2_/TBPB Mediated Oxidative Reaction of N-Tosylhydrazones with Anilines: Practical Construction of 1,4-Disubstituted 1,2,3-Triazoles under Metal-Free and Azide-Free Conditions. Org. Lett..

[28] Chen C., Xu X.-H., Yang B., Qing F.-L. (2014). Copper-Catalyzed Direct Trifluoromethylthiolation of Benzylic C–H Bonds via Nondirected Oxidative C(sp3)–H Activation. Org. Lett..

[29] Tang S., Liu K., Long Y., Gao X., Gao M., Lei A. (2015). Iodine-Catalyzed Radical Oxidative Annulation for the Construction of Dihydrofurans and Indolizines. Org. Lett..

[30] Chen W., Zhang Y., Li P., Wang L. (2018). tert-Butyl peroxybenzoate mediated formation of 3-alkylated quinolines from N-propargylamines via a cascade radical addition/cyclization reaction. Org. Chem. Front..

[31] Shen S.-J., Zhu C.-L., Lu D.-F., Xu H. (2018). Iron-Catalyzed Direct Olefin Diazidation via Peroxyester Activation Promoted by Nitrogen-Based Ligands. ACS Catal..

[32] Yu H., Li Z., Bolm C. (2018). Nondirected Copper-Catalyzed Sulfoxidations of Benzylic C–H Bonds. Org. Lett..

[33] Zhu N., Zhao J., Bao H. (2017). Iron catalyzed methylation and ethylation of vinyl arenes. Chem. Sci..

[34] Jian W., Ge L., Jiao Y., Qian B., Bao H. (2017). Iron-Catalyzed Decarboxylative Alkyl Etherification of Vinylarenes with Aliphatic Acids as the Alkyl Source. Angew. Chem. Int. Ed..

[35] Qian B., Chen S., Wang T., Zhang X., Bao H. (2017). Iron-Catalyzed Carboamination of Olefins: Synthesis of Amines and Disubstituted beta-Amino Acids. J. Am. Chem. Soc..

[36] Guo S., Wang Q., Jiang Y., Yu J.-T. (2014). tert-Butyl Peroxybenzoate-Promoted α-Methylation of 1,3-Dicarbonyl Compounds. J. Org. Chem..

[37] Bao X., Yokoe T., Ha T.M., Wang Q., Zhu J. (2018). Copper-catalyzed methylative difunctionalization of alkenes. Nat. Commun..

[38] Gray P., Williams A. (1959). The Thermochemistry And Reactivity Of Alkoxyl Radicals. Chem. Rev..

[39] Carey F.A., Sundberg R.J. (2007). Free Radical Reactions. Advanced Organic Chemistry, Part A: Structure and Mechanisms.

[40] Tran B.L., Driess M., Hartwig J.F. (2014). Copper-Catalyzed Oxidative Dehydrogenative Carboxylation of Unactivated Alkanes to Allylic Esters via Alkenes. J. Am. Chem. Soc..

[41] Tran B.L., Li B., Driess M., Hartwig J.F. (2014). Copper-Catalyzed Intermolecular Amidation and Imidation of Unactivated Alkanes. J. Am. Chem. Soc..

[42] Bunescu A., Wang Q., Zhu J. (2015). Synthesis of Functionalized Epoxides by Copper-Catalyzed Alkylative Epoxidation of Allylic Alcohols with Alkyl Nitriles. Org. Lett..

[43] Tang S., Wang P., Li H., Lei A. (2016). Multimetallic catalysed radical oxidative C(sp3)–H/C(sp)–H cross-coupling between unactivated alkanes and terminal alkynes. Nat. Commun..

[44] Wu X., Riedel J., Dong V.M. (2017). Transforming Olefins into γ,δ-Unsaturated Nitriles through Copper Catalysis. Angew. Chem. Int. Ed..

[45] Zhou H., Jian W., Qian B., Ye C., Li D., Zhou J., Bao H. (2017). Copper-Catalyzed Ligand-Free Diazidation of Olefins with TMSN3 in CH3CN or in H2O. Org. Lett..

[46] Newcomb M. (2012). Radical Kinetics and Clocks in Encyclopedia of Radicals in Chemistry, Biology and Materials.

[47] Pryor W.A., Gu J.T., Church D.F. (1985). Trapping free radicals formed in the reaction of ozone with simple olefins using 2,6-di-tert-butyl-4-cresol (BHT). J. Org. Chem..

[48] Thavasi V., Leong L.P., Bettens R.P. (2006). Investigation of the influence of hydroxy groups on the radical scavenging ability of polyphenols. J. Phys. Chem. A.

[49] Cheng G.-J., Zhang X., Chung L.W., Xu L., Wu Y.-D. (2015). Computational Organic Chemistry: Bridging Theory and Experiment in Establishing the Mechanisms of Chemical Reactions. J. Am. Chem. Soc..

[50] Plata R.E., Singleton D.A. (2015). A Case Study of the Mechanism of Alcohol-Mediated Morita Baylis–Hillman Reactions. The Importance of Experimental Observations. J. Am. Chem. Soc..

[51] Sameera W.M., Hatanaka M., Kitanosono T., Kobayashi S., Morokuma K. (2015). The Mechanism of Iron(II)-Catalyzed Asymmetric Mukaiyama Aldol Reaction in Aqueous Media: Density Functional Theory and Artificial Force-Induced Reaction Study. J. Am. Chem. Soc..

[52] Verma P., Varga Z., Klein J., Cramer C.J., Que L., Truhlar D.G. (2017). Assessment of electronic structure methods for the determination of the ground spin states of Fe(ii), Fe(iii) and Fe(iv) complexes. Phys. Chem. Chem. Phys..

[53] Mazumder S., Crandell D.W., Lord R.L., Baik M.-H. (2014). Switching the Enantioselectivity in Catalytic [4 + 1] Cycloadditions by Changing the Metal Center: Principles of Inverting the Stereochemical Preference of an Asymmetric Catalysis Revealed by DFT Calculations. J. Am. Chem. Soc..

[54] Lee Y., Baek S.-Y., Park J., Kim S.-T., Tussupbayev S., Kim J., Baik M.-H., Cho S.H. (2017). Chemoselective Coupling of 1,1-Bis[(pinacolato)boryl]alkanes for the Transition-Metal-Free Borylation of Aryl and Vinyl Halides: A Combined Experimental and Theoretical Investigation. J. Am. Chem. Soc..

[55] Zhu N., Wang T., Ge L., Li Y., Zhang X., Bao H. (2017). gamma-Amino Butyric Acid (GABA) Synthesis Enabled by Copper-Catalyzed Carboamination of Alkenes. Org. Lett..

[56] Huang X., Bergsten T.M., Groves J.T. (2015). Manganese-catalyzed late-stage aliphatic C-H azidation. J. Am. Chem. Soc..

[57] Toriyama F., Cornella J., Wimmer L., Chen T.G., Dixon D.D., Creech G., Baran P.S. (2016). Redox-Active Esters in Fe-Catalyzed C-C Coupling. J. Am. Chem. Soc..

[58] Lee W., Zhou J., Gutierrez O. (2017). Mechanism of Nakamura’s Bisphosphine-Iron-Catalyzed Asymmetric C(sp(2))-C(sp(3)) Cross-Coupling Reaction: The Role of Spin in Controlling Arylation Pathways. J. Am. Chem. Soc..

[59] Harvey J.N. (2014). Spin-forbidden reactions: Computational insight into mechanisms and kinetics. Wiley Interdiscip. Rev. Comput. Mol. Sci..

[60] Kulik H.J., Blasiak L.C., Marzari N., Drennan C.L. (2009). First-Principles Study of Non-heme Fe(II) Halogenase SyrB2 Reactivity. J. Am. Chem. Soc..

[61] Rana S., Biswas J.P., Sen A., Clémancey M., Blondin G., Latour J.-M., Rajaraman G., Maiti D. (2018). Selective C–H halogenation over hydroxylation by non-heme iron(iv)-oxo. Chem. Sci..

[62] Shin K., Park Y., Baik M.-H., Chang S. (2017). Iridium-catalysed arylation of C–H bonds enabled by oxidatively induced reductive elimination. Nat. Chem..

[63] Chiou M.-F., Jayakumar J., Cheng C.-H., Chuang S.-C. (2018). Impact of the Valence Charge of Transition Metals on the Cobalt- and Rhodium-Catalyzed Synthesis of Indenamines, Indenols, and Isoquinolinium Salts: A Catalytic Cycle Involving MIII/MV M = Co, Rh for 4 + 2 Annulation. J. Org. Chem..

[64] Fu N., Sauer G.S., Saha A., Loo A., Lin S. (2017). Metal-catalyzed electrochemical diazidation of alkenes. Science.

[65] Chen B., Fang C., Liu P., Ready J.M. (2017). Rhodium-Catalyzed Enantioselective Radical Addition of CX_4_ Reagents to Olefins. Angew. Chem. Int. Ed..

[66] Lo J.C., Kim D., Pan C.-M., Edwards J.T., Yabe Y., Gui J., Qin T., Gutierrez S., Giacoboni J., Smith M.W. (2017). Fe-Catalyzed C-C Bond Construction from Olefins via Radicals. J. Am. Chem. Soc..

[67] Jang E.S., McMullin C.L., Käß M., Meyer K., Cundari T.R., Warren T.H. (2014). Copper(II) Anilides in sp3 C-H Amination. J. Am. Chem. Soc..

[68] Gephart Iii R.T., Huang D.L., Aguila M.J.B., Schmidt G., Shahu A., Warren T.H. (2012). Catalytic C–H Amination with Aromatic Amines. Angew. Chem. Int. Ed..

[69] Carsch K.M., DiMucci I.M., Iovan D.A., Li A., Zheng S.-L., Titus C.J., Lee S.J., Irwin K.D., Nordlund D., Lancaster K.M. (2019). Synthesis of a copper-supported triplet nitrene complex pertinent to copper-catalyzed amination. Science.

[70] Lee C., Yang W., Parr R.G. (1988). Development of the Colle-Salvetti correlation-energy formula into a functional of the electron density. Phys. Rev. B.

[71] Becke A.D. (1993). Density-functional thermochemistry. III. The role of exact exchange. J. Chem. Phys..

[72] Grimme S., Antony J., Ehrlich S., Krieg H. (2010). A consistent and accurate ab initio parametrization of density functional dispersion correction (DFT-D) for the 94 elements H-Pu. J. Chem. Phys..

[73] Grimme S., Ehrlich S., Goerigk L. (2011). Effect of the damping function in dispersion corrected density functional theory. J. Comput. Chem..

[74] Weigend F. (2006). Accurate Coulomb-fitting basis sets for H to Rn. Phys. Chem. Chem. Phys..

[75] Weigend F., Ahlrichs R. (2005). Balanced basis sets of split valence, triple zeta valence and quadruple zeta valence quality for H to Rn: Design and assessment of accuracy. Phys. Chem. Chem. Phys..

[76] Peng C., Bernhard Schlegel H. (1993). Combining Synchronous Transit and Quasi-Newton Methods to Find Transition States. Isr. J. Chem..

[77] Peng C., Ayala P.Y., Schlegel H.B., Frisch M.J. (1996). Using redundant internal coordinates to optimize equilibrium geometries and transition states. J. Comput. Chem..

[78] Besalú E., Bofill J.M. (1998). On the automatic restricted-step rational-function-optimization method. Theor. Chem. Acc..

[79] Marenich A.V., Cramer C.J., Truhlar D.G. (2009). Universal Solvation Model Based on Solute Electron Density and on a Continuum Model of the Solvent Defined by the Bulk Dielectric Constant and Atomic Surface Tensions. J. Phys. Chem. B.

[80] Harvey J.N., Aschi M., Schwarz H., Koch W. (1998). The singlet and triplet states of phenyl cation. A hybrid approach for locating minimum energy crossing points between non-interacting potential energy surfaces. Theor. Chem. Acc..

[81] Frisch M.J., Trucks G.W., Schlegel H.B., Scuseria G.E., Robb M.A., Cheeseman J.R., Scalmani G., Barone V., Mennucci B., Petersson G.A. (2013). Gaussian ~09 Revision D.01.

