# Supplementary material for: Revealing the Iron-Catalyzed β-Methyl Scission of tert-Butoxyl Radicals via the Mechanistic Studies of Carboazidation of Alkenes
Source: Molecules. 2020 Mar 9;25(5):1224. doi: 10.3390/molecules25051224 (PMC7179474; doi:10.3390/molecules25051224)
Supplement: Supplementary file 1 [file molecules-25-01224-s001.pdf]

Supporting Information

Revealing the Iron-Catalyzed β-Methyl Scission of *tert*-Butoxyl Radicals via the Mechanistic Studies of Carboazidation of Alkenes

Mong-Feng Chiou [1], Haigen Xiong [1,2], Yajun Li [1], Hongli Bao[1,2,*] and Xinhao Zhang [3,*]

[1] Key Laboratory of Coal to Ethylene Glycol and Its Related Technology, State Key Laboratory of Structural Chemistry, Center for Excellence in Molecular Synthesis, Fujian Institute of Research on the Structure of Matter, Chinese Academy of Sciences, 155 Yangqiao Road West, Fuzhou, Fujian 350002, China; qiumengfeng@fjirsm.ac.cn (M.-F.C.); xionghaigen@fjirsm.ac.cn (H.X.); liyajun@fjirsm.ac.cn (Y.L.)

[2] School of Chemistry and Chemical Engineering of University of Chinese Academy of Sciences, Beijing, 100049, China.

[3] Lab of Computational Chemistry and Drug Design, Key Laboratory of Chemical Genomics, Peking University Shenzhen Graduate School, Shenzhen 518055, China.

Contents

Preliminary mechanistic studies 3

(a) Controlled experiments without alkyl iodide 3

(b) Ring opening experiment 4

(c) Ring closure reaction 5

(d) GC-MS analysis of the crude mixture 6

(d.i) GC-MS observation of product 9 and trimethylsilyl benzoate 8

(d.ii) GC-MS observation of acetone, CH_3I and DME 10

(e) Radical trapping experiments 12

(e.i) With BHT 12

(e.ii) With hydroquinone 12

References for experimental studies 13

Copies of ^1H, ^{19}F and ^{13}C NMR spectra 14

Computational data 20

Figures and Tables 20

Coordinate of optimized structures 28

Preliminary mechanistic studies

(a) Controlled experiments without alkyl iodide

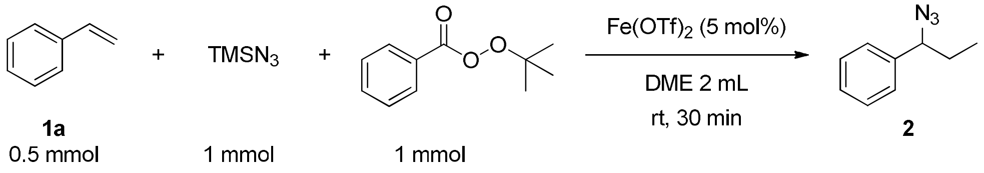

To a dried Schlenk tube equipped with a magnetic bar, $Fe(OTf)_2$ (9 mg, 0.025 mmol) was added, flushed with nitrogen gas (3 times), and maintained the nitrogen atmosphere using the balloon. A thoroughly mixed solution of vinylarene (0.5 mmol), $TMSN_3$ (1.0 mmol) and TBPB (1.0 mmol) in DME (2 mL) was added to the catalyst via syringe and stirred vigorously for 30 minutes at room temperature. The solvent was evaporated and the residue was purified by flash chromatography on silica gel to give the corresponding product **2** in 52% yield.

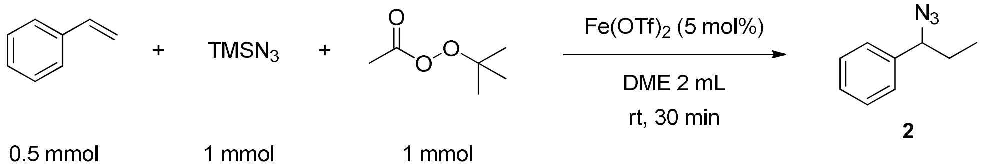

To a dried Schlenk tube equipped with a magnetic bar, $Fe(OTf)_2$ (9 mg, 0.025 mmol) was added, flushed with nitrogen gas (3 times) and maintained the nitrogen atmosphere using the balloon. A thoroughly mixed solution of vinylarene (0.5 mmol), $TMSN_3$ (1.0 mmol) and tert-butyl ethaneperoxoate (1.0 mmol) in DME (2 mL) was added to the catalyst via syringe and stirred vigorously for 30 minutes at room temperature. The solvent was evaporated and the residue was purified by flash chromatography on silica gel to give the corresponding product **2** in 33% yield.

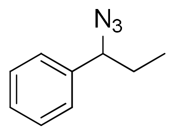

2

2: clear oil; IR (KBr): υ 2927, 2096, 1454, 1242, 699 cm^{-1}; ^{1}H NMR (400 MHz, $CDCl_3$) δ 7.41 – 7.26 (m, 5H), 4.34 (t, *J* = 7.1 Hz, 1H), 1.93 – 1.72 (m, 2H), 0.93 (t, *J* = 7.4 Hz, 3H); ^{13}C NMR (100 MHz, $CDCl_3$) δ 139.70, 128.73, 128.16, 126.94, 67.90, 29.32, 10.77. The NMR data is consistent with the reported value. [1]

(b) Ring opening experiment

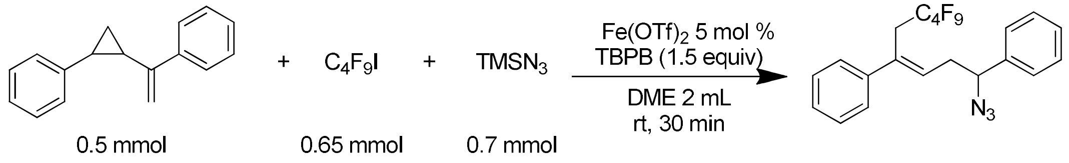

3 **4**

To a dried Schlenk tube equipped with a magnetic bar, $Fe(OTf)_2$ (9 mg, 0.025 mmol) was added. This tube was flushed with nitrogen gas (3 times) and maintained a nitrogen atmosphere using a nitrogen balloon. A thoroughly mixed solution of vinylarene **3** (0.5 mmol), [2] alkyl iodide (0.65 mmol), $TMSN_3$ (0.7 mmol) and TBPB (0.75 mmol) in DME (2 mL) was added to the catalyst via syringe and the mixture was stirred vigorously for 30 minutes at room temperature. The solvent was then evaporated and the residue was purified by flash chromatography on silica gel to give the corresponding product **4** in 42% yield (E/Z = 17:1).

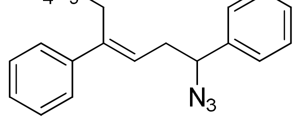

4

4: Yield: 102 mg, 42% (E/Z = 17:1); clear oil; IR (KBr): υ 3032, 2099, 1236, 1133,

913, 749 cm^{-1}; ^{1}H NMR (400 MHz, $CDCl_3$) δ 7.43 – 7.24 (m, 10H), 6.00 (t, *J* = 7.4 Hz, 1H), 4.59 (t, *J* = 7.0 Hz, 1H), 3.21 (t, *J* = 18.3 Hz, 2H), 2.77 – 2.59 (m, 2H); ^{19}F NMR (376 MHz, $CDCl_3$) δ −79.38 – −82.74 (m, 3F), −110.28 – −112.60 (m, 2F), −122.65 – −124.90 (m, 2F), −125.05 – −127.45 (m, 2F); ^{13}C NMR (100 MHz, $CDCl_3$) δ 142.00, 138.93, 131.54, 131.33, 128.93, 128.53, 128.45, 127.56, 126.79, 126.34, 65.78, 36.39, 31.20 (t, *J* = 22.0 Hz). The NMR data is consistent with the reported value. [3]

(c) Ring closure reaction

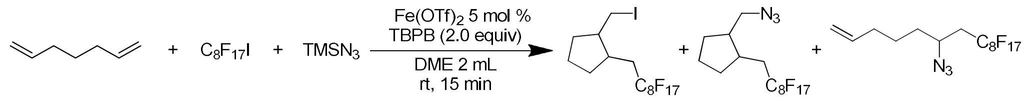

5, 0.5 mmol   0.65 mmol   1 mmol   **6**   **7**   **8**

To a dried Schlenk tube equipped with a magnetic bar, $Fe(OTf)_2$ (9 mg, 0.025 mmol) was added. This tube was flushed with nitrogen gas (3 times) and maintained a nitrogen atmosphere using a nitrogen balloon. A thoroughly mixed solution of alkene (0.5 mmol), alkyl iodide (0.65 mmol), $TMSN_3$ (1.0 mmol) and TBPB (1.0 mmol) in DME (2 mL) was added to the catalyst via syringe and the mixture was stirred vigorously for 15 minutes at room temperature. The solvent was then evaporated and the residue was purified by flash chromatography on silica gel to give the corresponding products **6** (31%, dr = 3.8:1), **7** (49%, dr = 5.2:1), and **8** (18%).

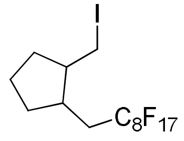

6

6: Yield: 100 mg, 31% (dr = 3.8:1); clear oil; IR (KBr): υ 2961, 2877, 1456, 1437, 1257, 1051, 966, 705, 658 cm^{-1}; ^{1}H NMR (400 MHz, $CDCl_3$) δ 3.19 (dd, *J* = 9.7, 5.6 Hz, 1H), 3.00 (t, *J* = 9.8 Hz, 1H), 2.54 – 2.10 (m, 3H), 2.04 – 1.84 (m, 3H), 1.81 – 1.40 (m, 4H); ^{19}F NMR (376 MHz, $CDCl_3$) δ −79.19 – −82.26 (m, 3F), −110.95 – −115.70 (m, 2F), −118.25 – −125.47 (m, 10F), −125.64 – −127.56 (m, 2F); ^{13}C NMR (100 MHz, $CDCl_3$) δ 45.70, 35.83, 31.78, 30.44 (d, *J* = 2.1 Hz), 30.04 (t, *J* = 21.6 Hz), 21.99, 7.36; HRMS (EI) calcd for $[C_{15}H_{12}F_{17}I]^+$($[M]^+$): 641.9712, found: 641.9716.

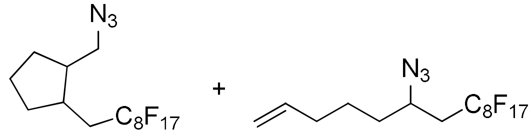

7 **8**

7: Yield: 49% (dr = 5.2:1); **8**: Yield: 18%; clear oil; IR (KBr): υ 2965, 2101, 1206, 1149, 913, 748 cm^{-1}; ^{1}H NMR (400 MHz, $CDCl_3$) δ 5.88 – 5.70 (m, 0.43H), 5.03 (t, *J* = 14.2 Hz, 0.86H), 3.84 – 3.67 (m, 0.43H), 3.30 (dd, *J* = 12.2, 6.2 Hz, 1H), 3.16 (dd, *J* = 12.2, 7.8 Hz, 1H), 2.41 – 1.39 (m, 15.34H); ^{19}F NMR (376 MHz, $CDCl_3$) δ −78.69 – −83.33 (m, 3F), −110.53 – −116.20 (m, 2F), −119.58 – −125.41 (m, 10F), −125.79 – −127.47 (m, 2F); ^{13}C NMR (100 MHz, $CDCl_3$) δ 137.66, 115.37, 77.32, 77.00, 76.68, 55.83, 55.19, 51.97, 45.54, 41.56, 35.65 (t, *J* = 21.1 Hz), 34.77, 34.65, 33.11, 31.09, 31.07, 30.60 (t, *J* = 21.6 Hz), 29.70, 28.85, 24.88, 24.08, 22.36; HRMS (EI) calcd for $[C_{15}H_{12}F_{17}N]^+$($[M{-}N_2]^+$): 529.0698, found: 529.0701.

(d) GC-MS analysis of the crude mixture

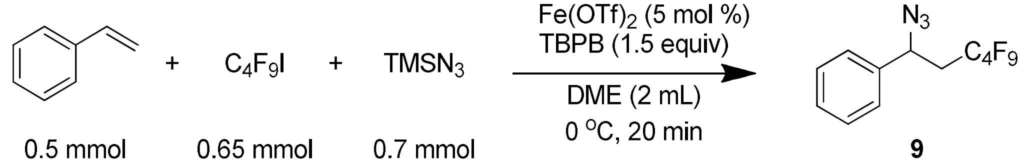

9

To a dried Schlenk tube equipped with a magnetic bar, $Fe(OTf)_2$ (9 mg, 0.025 mmol) was added. This tube was flushed with nitrogen gas (3 times) and maintained a nitrogen atmosphere using a nitrogen balloon. A thoroughly mixed solution of vinylarene (0.5 mmol), alkyl iodide (0.65 mmol), $TMSN_3$ (0.7 mmol) and TBPB (0.75 mmol) in DME (2 mL) was added to the catalyst via syringe and the mixture was stirred vigorously for 20 minutes at 0 ^{o}C. *The reaction mixture was filtered and detected by GC-MS (Ion trap, EI). Acetone (m/z (%) = 58.04 [M$^+$] (100)), CH_3I (m/z (%) = 141.94 [M$^+$] (100)), DME (m/z (%) = 90.06 [M$^+$] (100)), product* ***9*** *(m/z (%) = 365.08 [M$^+$] (100)) and trimethylsilyl benzoate (m/z (%) = 194.08 [M$^+$] (100)) could be found by GC-MS.* The solvent was then evaporated and the residue was purified by flash chromatography on silica gel to give the corresponding product **9** in 78% yield.

(**d.i**) GC-MS observation of product **9** and trimethylsilyl benzoate

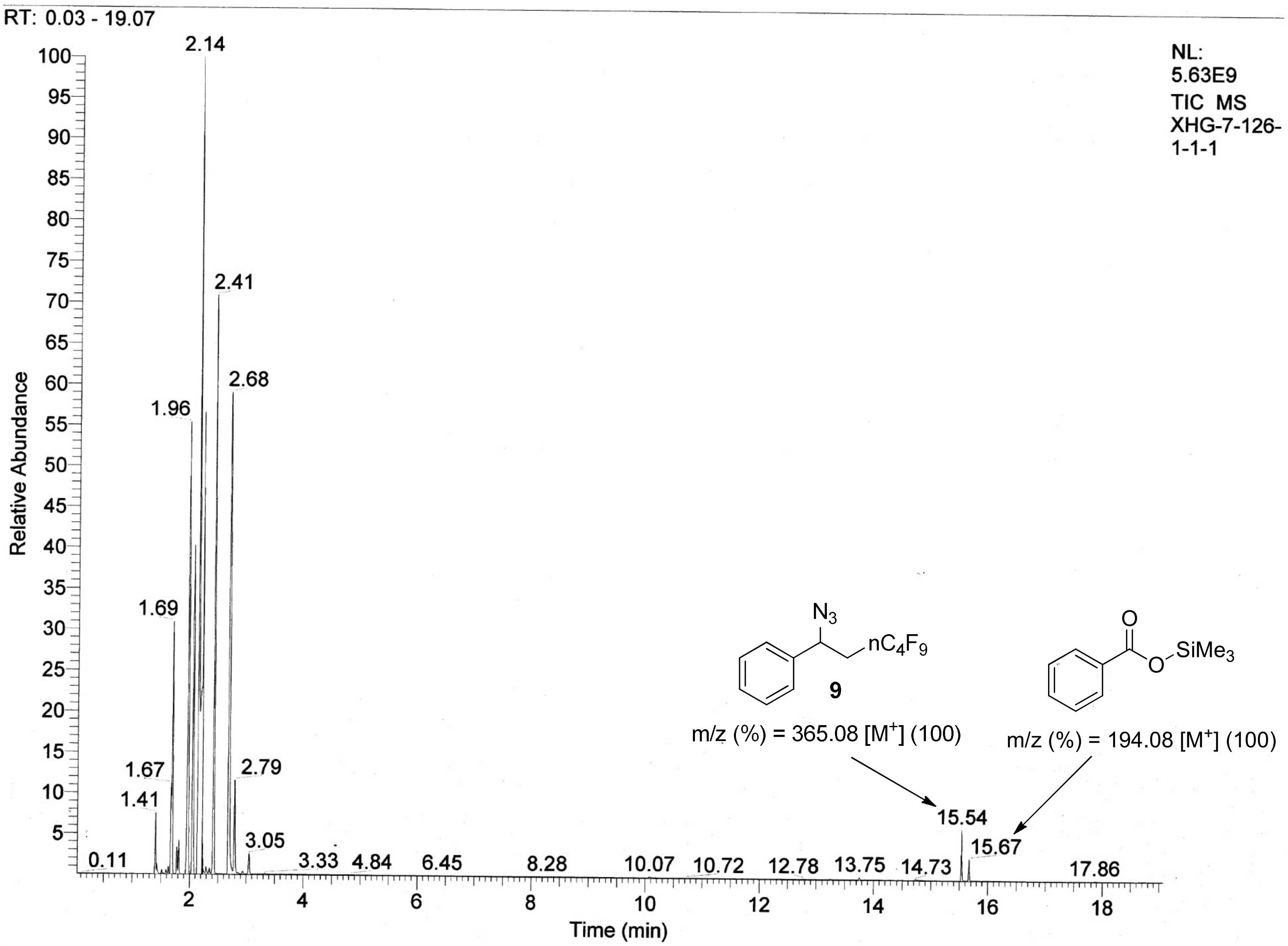

(**d.ii**) GC-MS observation of acetone, CH_3I and DME

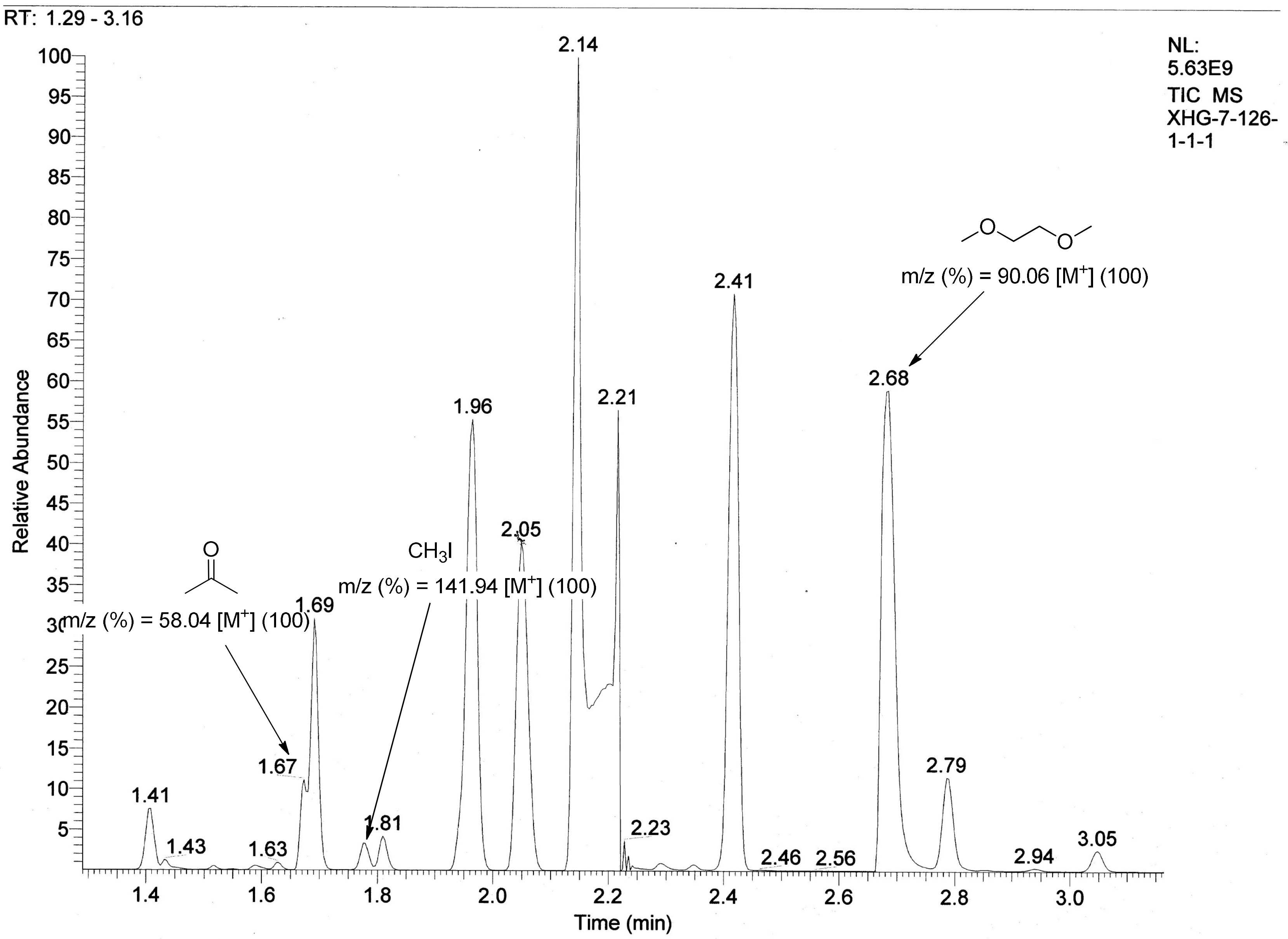

(e) Radical trapping experiments

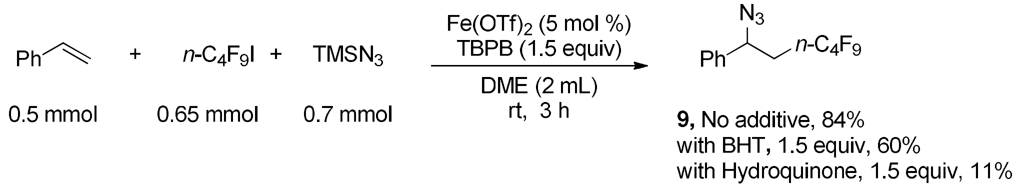

When radical trapping experiments are performed with 2,6-di-tert-butyl-4-methylphenol (BHT) or hydroquinone as a radical scavenger, the yields drops to 60% and 11% respectively. The two experiments suggested that this reaction might be hampered by the radical scavenger.

(**e.i**) With BHT

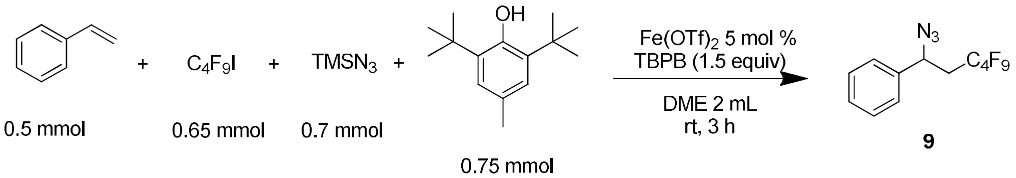

To a dried Schlenk tube equipped with a magnetic bar, $Fe(OTf)_2$ (9 mg, 0.025 mmol) was added. This tube was flushed with nitrogen gas (3 times) and maintained a nitrogen atmosphere using a nitrogen balloon. A thoroughly mixed solution of vinylarene (0.5 mmol), alkyl iodide (0.65 mmol), $TMSN_3$ (0.7 mmol), TBPB (0.75 mmol) and BHT (2,6-di-tert-butyl-4-methylphenol) (0.75 mmol) in DME (2 mL) was added to the catalyst via syringe and the mixture was stirred vigorously for 3 h at room temperature. The solvent was then evaporated and the residue was purified by flash chromatography on silica gel to give the corresponding product **9** in 60% yield.

(**e.ii**) With hydroquinone

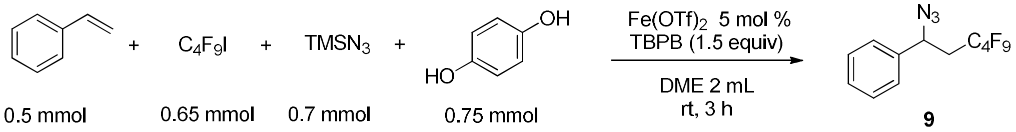

To a dried Schlenk tube equipped with a magnetic bar, $Fe(OTf)_2$ (9 mg, 0.025 mmol) was added. This tube was flushed with nitrogen gas (3 times) and maintained a nitrogen atmosphere using a nitrogen balloon. A thoroughly mixed solution of vinylarene (0.5 mmol), alkyl iodide (0.65 mmol), $TMSN_3$ (0.7 mmol), TBPB (0.75 mmol) and hydroquinone (0.75 mmol) in DME (2 mL) was added to the catalyst via syringe and the mixture was stirred vigorously for 3 h at room temperature. The solvent was then evaporated and the residue was purified by flash chromatography on silica gel to give the corresponding product **9** in 11% yield.

References for experimental studies

1. Huang, X.; Bergsten, T. M.; Groves, J. T., Manganese-catalyzed late-stage aliphatic C-H azidation. *J. Am. Chem. Soc.* **2015,** *137*, 5300–5303.

2. Zhang, Z.-Q.; Meng, X.-Y.; Sheng, J.; Lan, Q.; Wang, X.-S., Enantioselective Copper-Catalyzed 1,5-Cyanotrifluoromethylation of Vinylcyclopropanes. *Org. Lett.* **2019,** *21*, 8256–8260.

3. Geng, X.; Lin, F.; Wang, X.; Jiao, N., Azidofluoroalkylation of Alkenes with Simple Fluoroalkyl Iodides Enabled by Photoredox Catalysis. *Org. Lett.* **2017**, *19*, 4738–4741.

Copies of ^1H, ^{19}F and ^{13}C NMR spectra

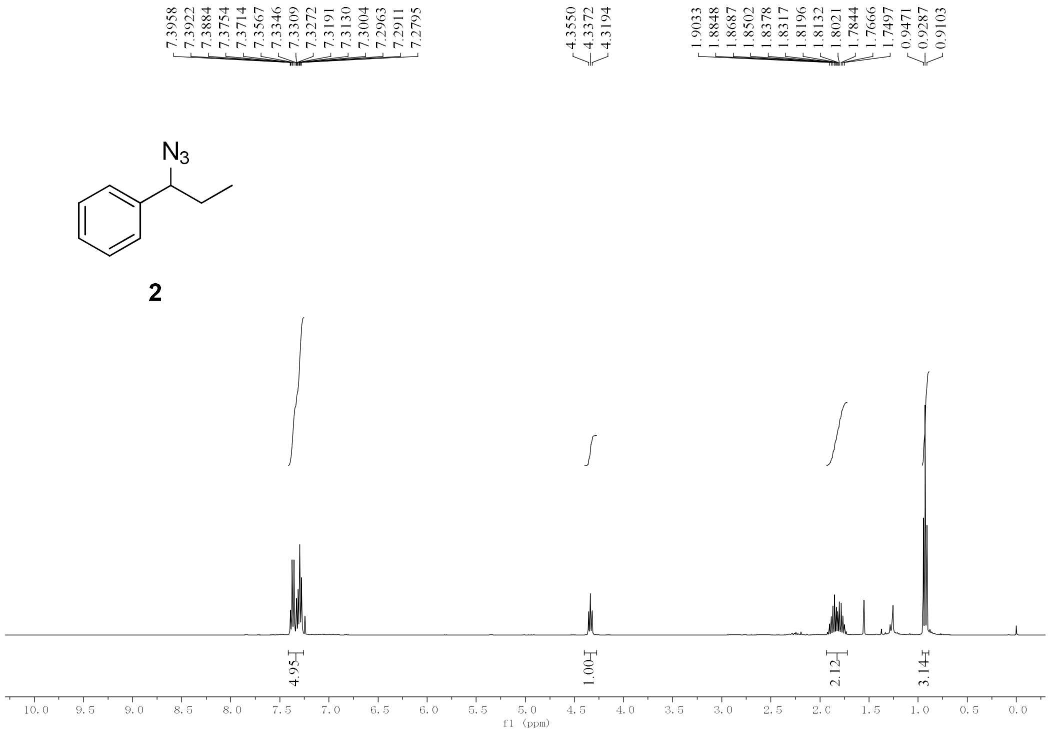

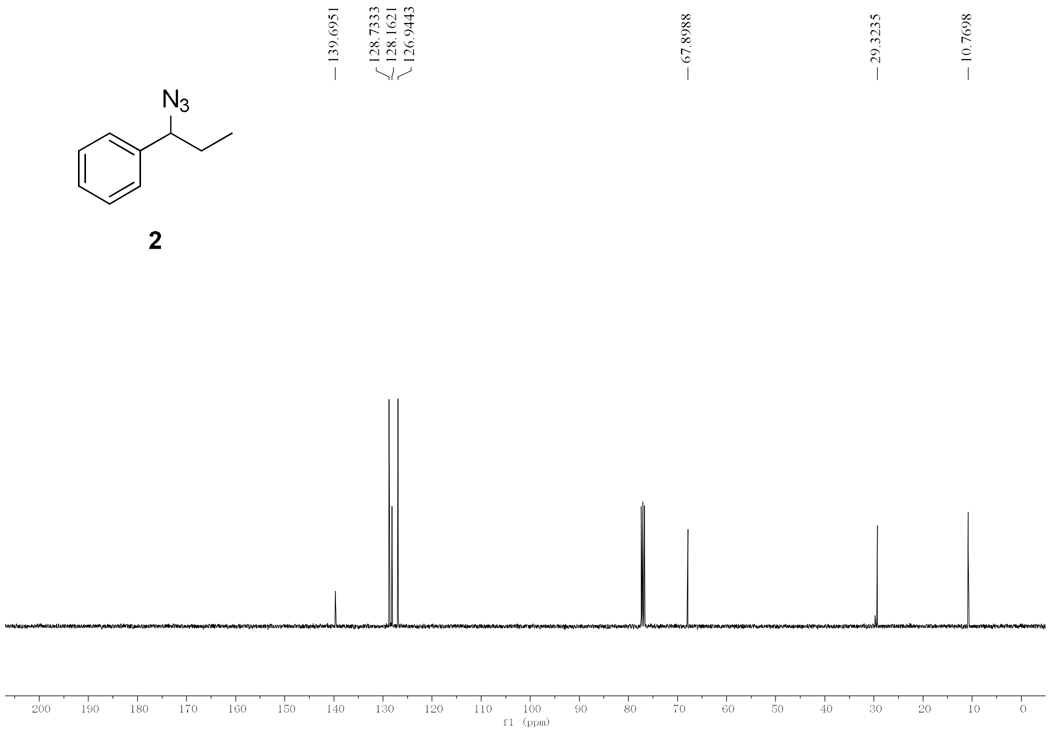

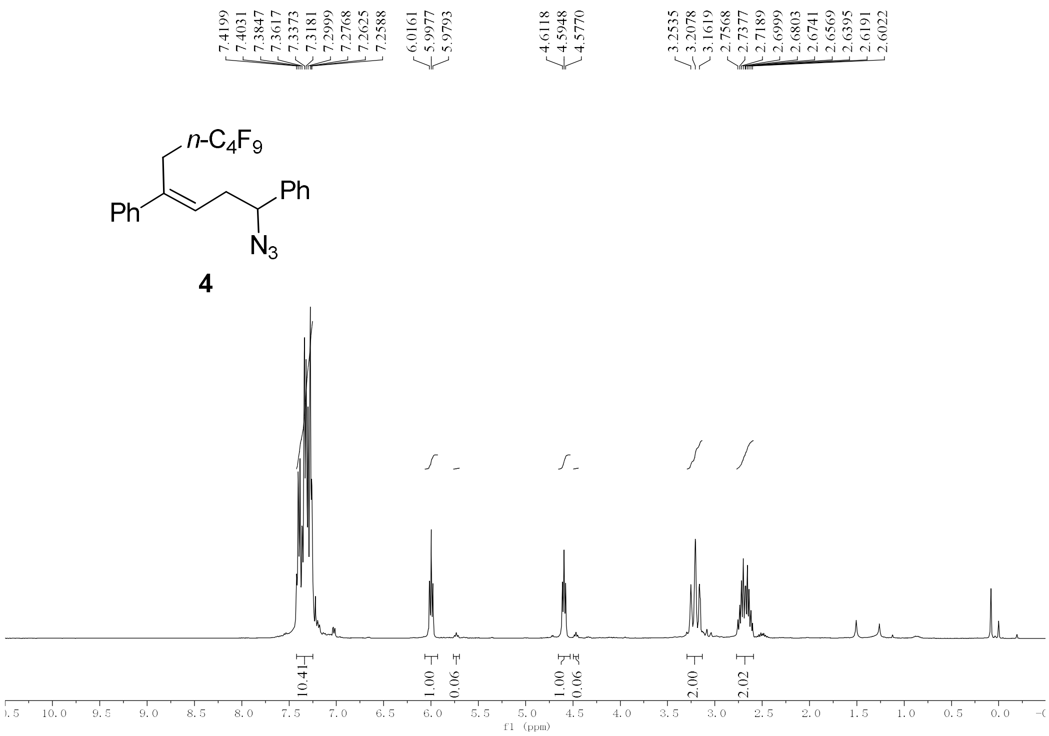

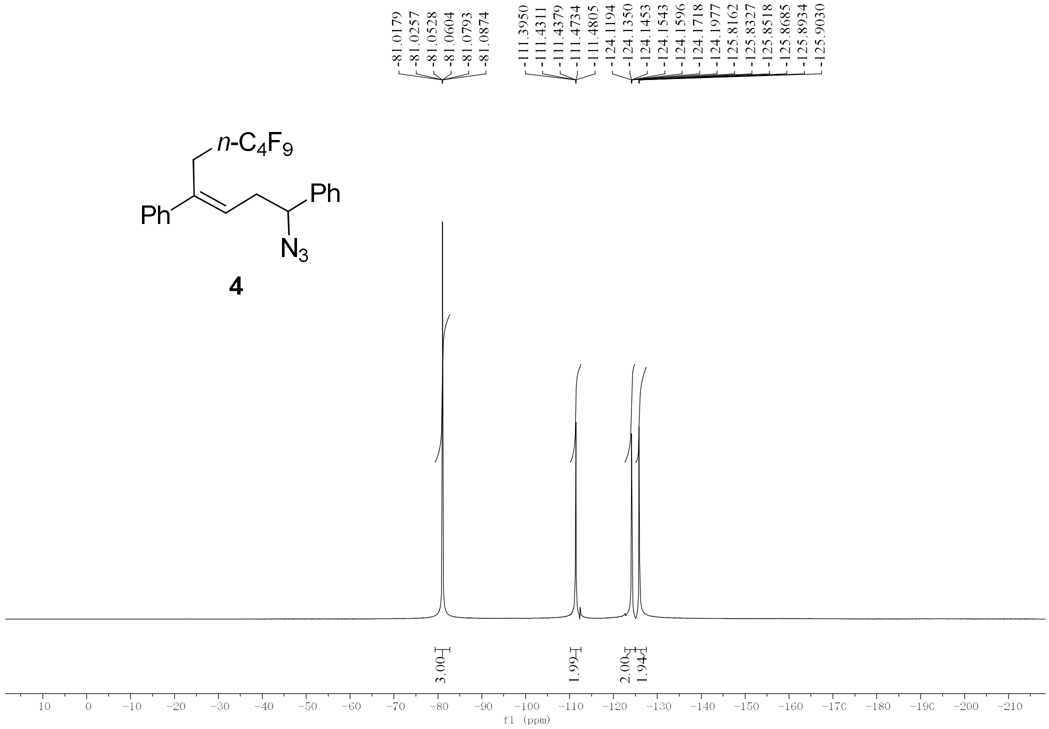

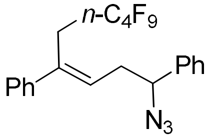
n-C4F9
Ph
Ph
N3

4

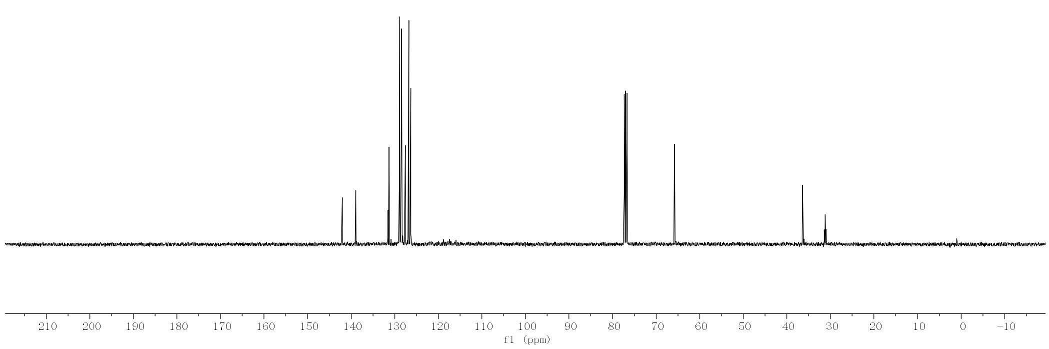
142.0041
138.9275
131.5423
131.3335
128.9262
128.5291
128.4501
127.5644
126.7856
126.3402
65.7812
36.3868
31.4160
31.1966
30.9782
210 200 190 180 170 160 150 140 130 120 110 100 90 80 70 60 50 40 30 20 10 0 -10
f1 (ppm)

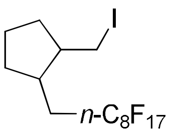
I
n-C8F17

6

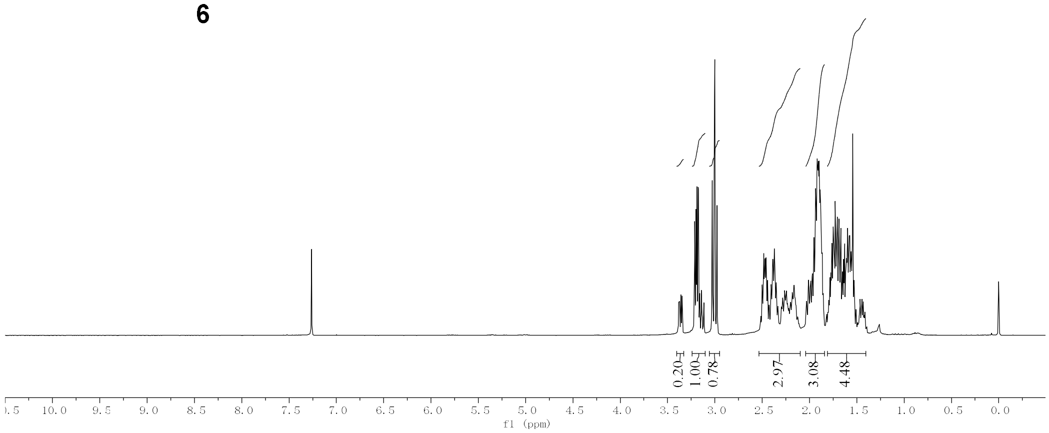
3.2132
3.1994
3.1891
3.1752
3.0265
3.0020
2.9775
2.4994
2.4841
2.4708
2.4593
2.4439
2.4042
2.3887
2.3722
2.3523
2.2823
2.2625
2.2446
2.1819
2.1630
2.0095
1.9882
1.9698
1.9516
1.9358
1.9192
1.9135
1.9058
1.8972
1.8881
1.8824
1.8697
1.8654
1.7491
1.7276
1.7066
1.6900
1.6691
1.6286
1.6058
1.5968
1.5822
1.5751
1.5423
1.4645
1.4451
1.4327
0.0000
0.20
1.00
0.78
2.97
3.08
4.48
10.0 9.5 9.0 8.5 8.0 7.5 7.0 6.5 6.0 5.5 5.0 4.5 4.0 3.5 3.0 2.5 2.0 1.5 1.0 0.5 0.0
f1 (ppm)

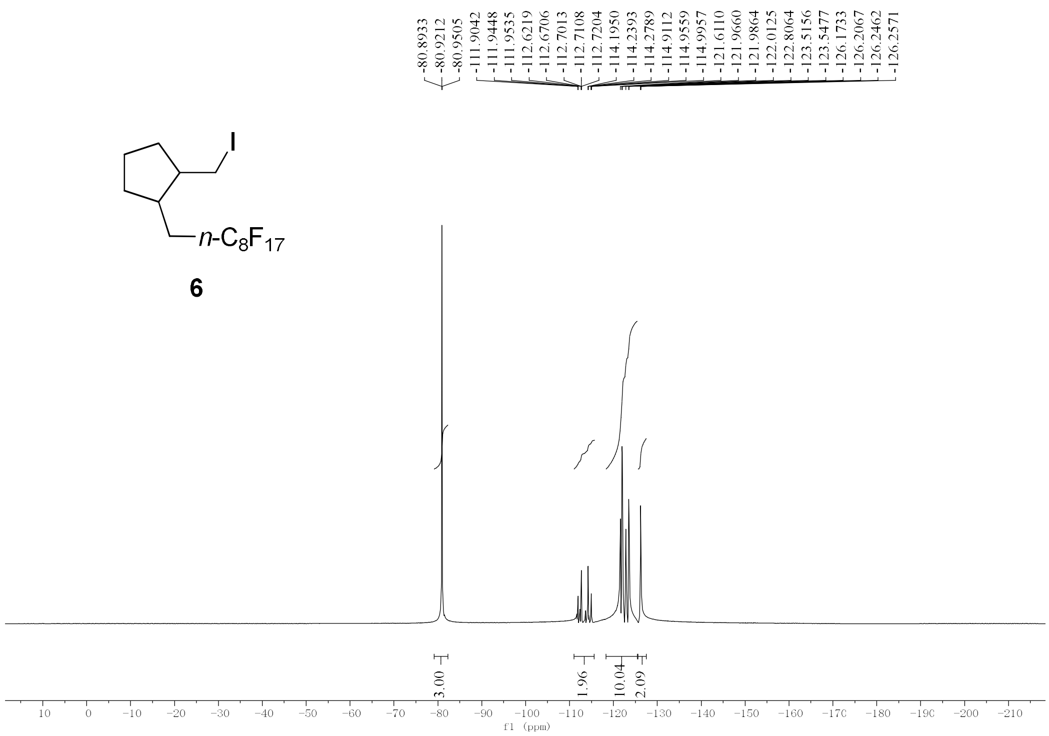

6

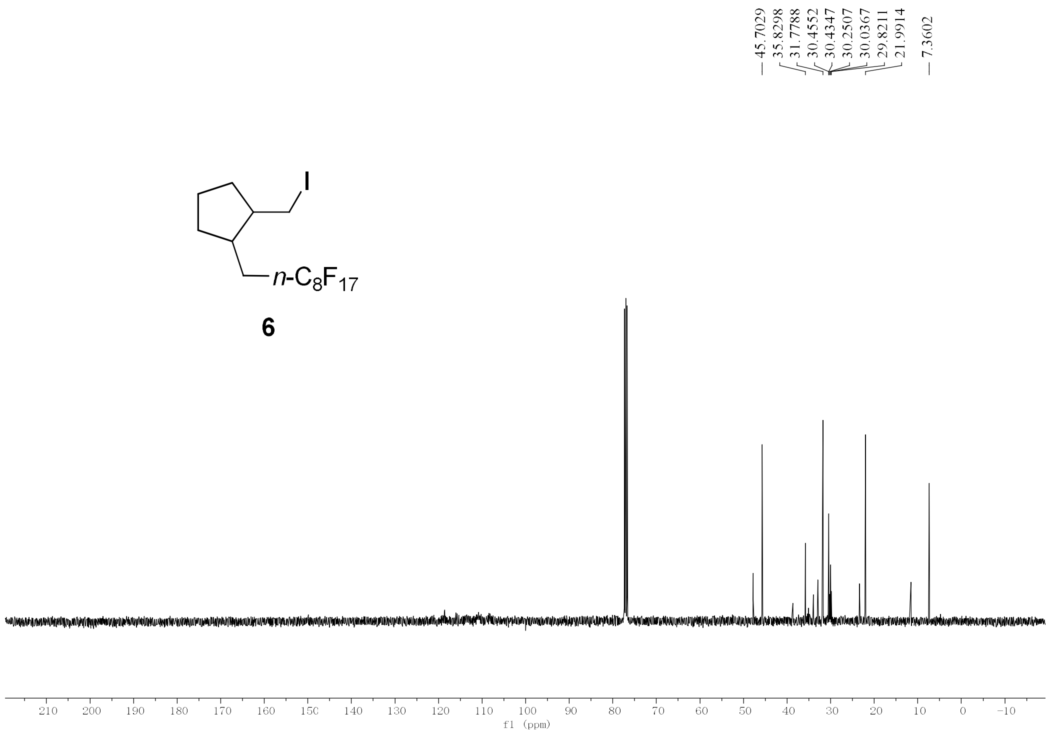

6

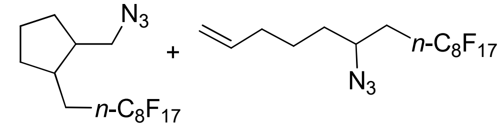

N3
+
n-C8F17
n-C8F17
N3

7 **8**

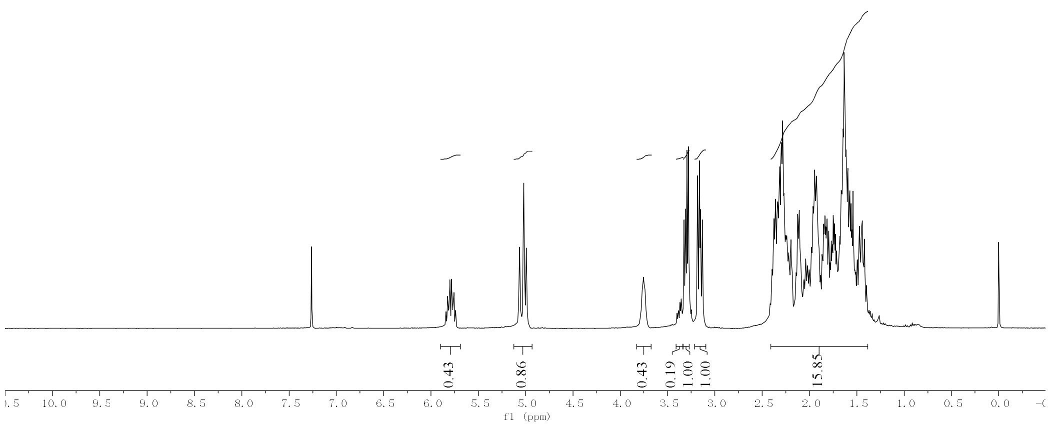

5.8425
5.8257
5.8169
5.8087
5.8001
5.7832
5.7742
5.7662
5.7576
5.7410
5.0641
5.0203
4.9932
3.7552
3.3251
3.3094
3.2944
3.2789
3.1821
3.1625
3.1515
3.1320
2.3593
2.3372
2.3161
2.2977
2.2839
2.2688
2.1240
2.1087
1.9618
1.9447
1.9265
1.8502
1.8420
1.8362
1.8131
1.7485
1.7372
1.6455
1.6334
1.6133
1.4716
1.4505
1.4407
1.4190
-0.0000
0.43
0.86
0.43
0.19
1.00
1.00
15.85
f1 (ppm)

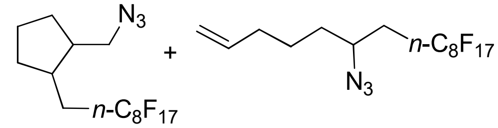

N3
+
n-C8F17
n-C8F17
N3

7 **8**

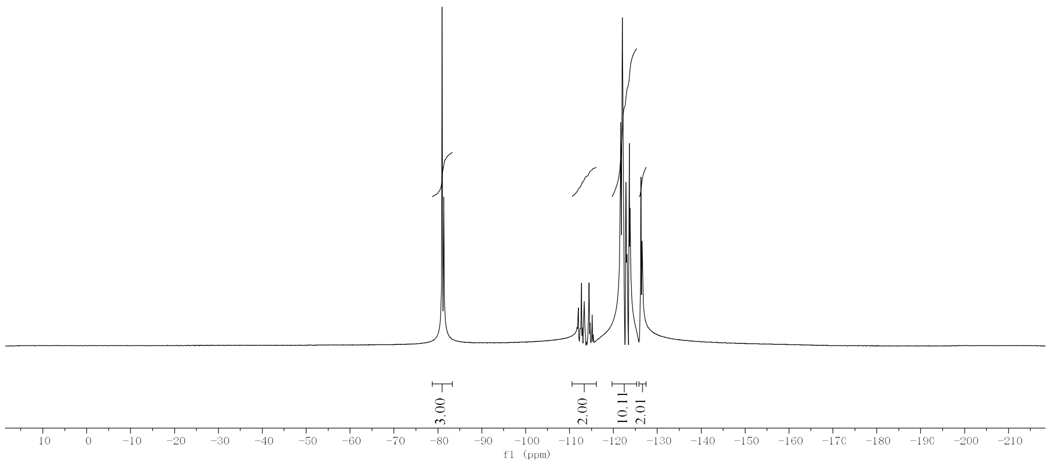

-80.9266
-80.9555
-80.9844
-81.3319
-81.3580
-112.0113
-112.6888
-112.7304
-112.7708
-113.3388
-113.3749
-113.4215
-114.3961
-114.4364
-114.4765
-115.1518
-121.6634
-122.0310
-122.2075
-122.8453
-123.0556
-123.6044
-123.8073
-126.2483
-126.2713
-126.5315
3.00
2.00
10.11
2.01
f1 (ppm)

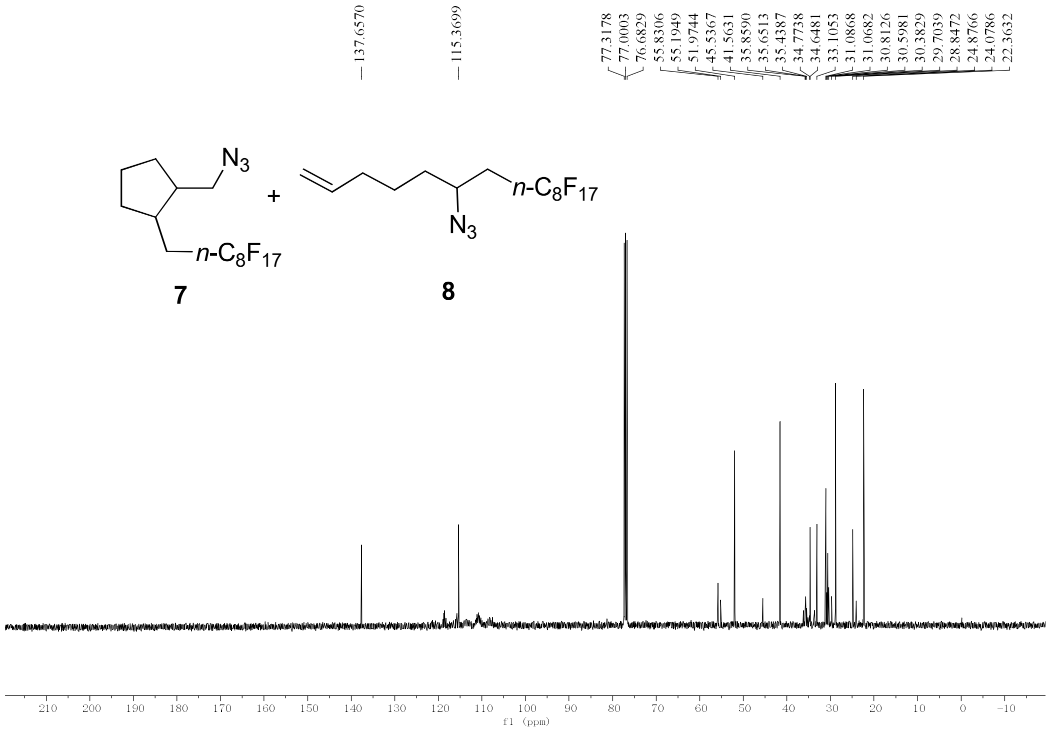
137.6570
115.3699
77.3178
77.0003
76.6829
55.8306
55.1949
51.9744
45.5367
41.5631
35.8590
35.6513
35.4387
34.7738
34.6481
33.1053
31.0868
31.0682
30.8126
30.5981
30.3829
29.7039
28.8472
24.8766
24.0786
22.3632
N3
n-C8F17
7
+
8
210 200 190 180 170 160 150 140 130 120 110 100 90 80 70 60 50 40 30 20 10 0 -10
f1 (ppm)

Computational data

Figures and Tables

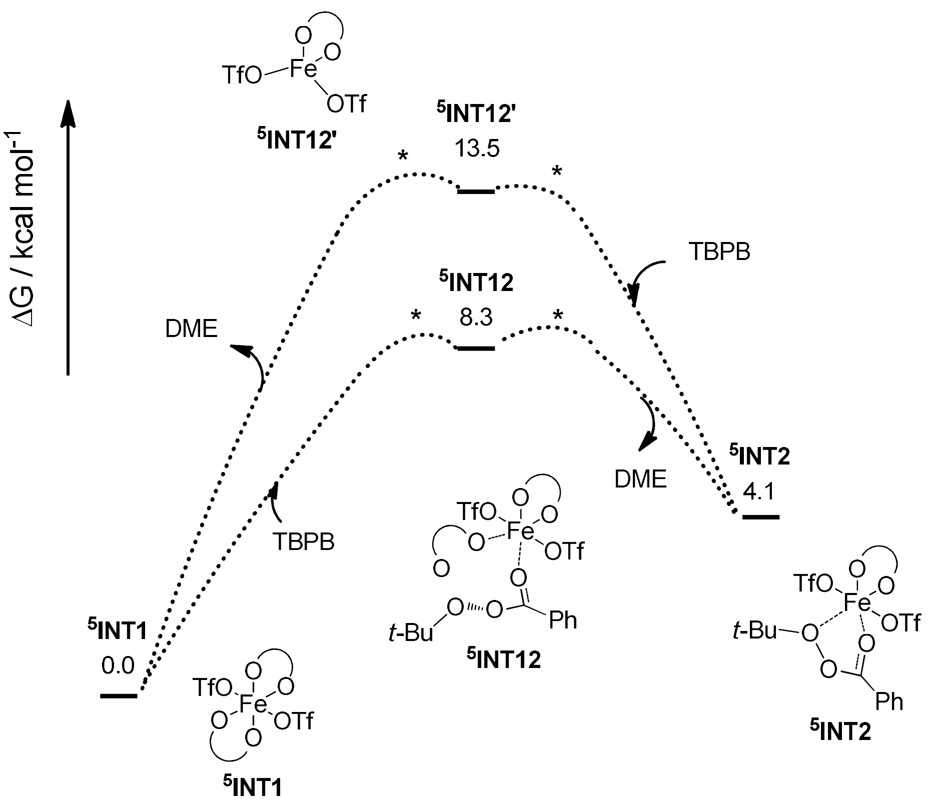

Figure S1. The Gibbs free energy profile corresponding to TBPB association to ^{5}**INT1** and DME dissociation. Intermediate ^{5}**INT12** is found by approaching a TBPB to ^{5}**INT1** with relative free energy 8.3 kcal/mol. Two transition states corresponding to two O-Fe bond of DME cleavages in both association and dissociation ligand exchange pathways indicated by * were not explicitly located.

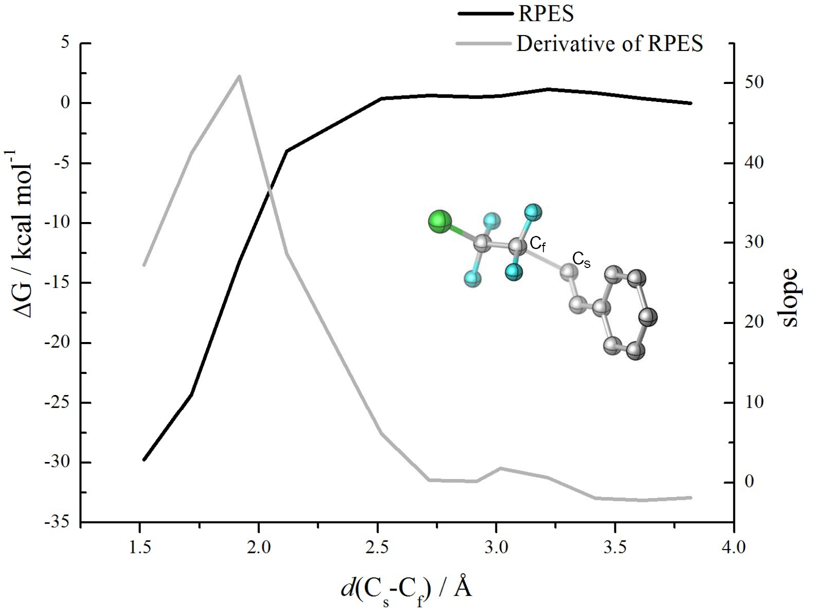

Figure S2. The relaxed potential energy surface (RPES) scan along d(C_s-C_f) for perhaloalkyl radical **Rf1** addition to styrene. The Derivative of RPES (gray line) becomes positive at 3.22 Å implying that the interaction between C_s and C_f turns to be an attractive force. Relative free energies are in kcal/mol.

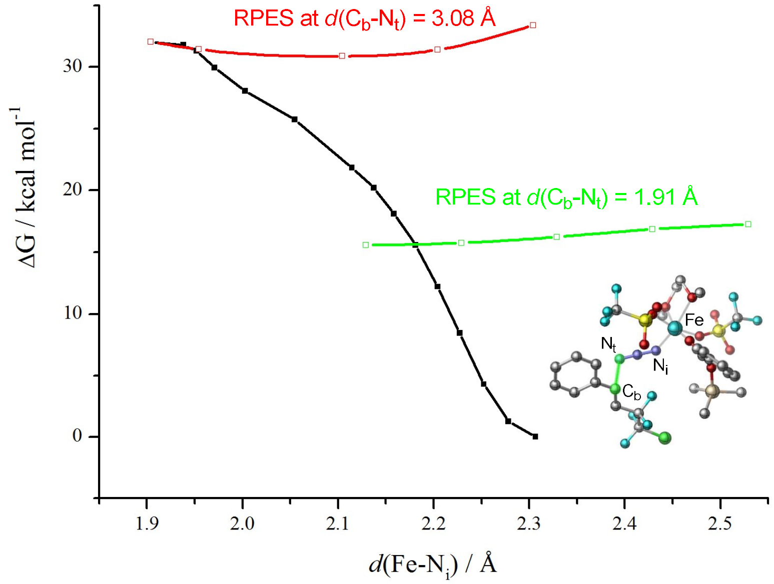

Figure S3. The relaxed potential energy surfaces (RPESs) of **^{2}INT10** directly coupling with the terminal nitrogen atom of azide in **^{6}INT6** scanned along d(Fe-N_i) on quintet state surface. Quintet PSEs fixing at d(C_b-N_t) equal to 3.08 and 1.91 Å are also shown by the red and green line, respectively.

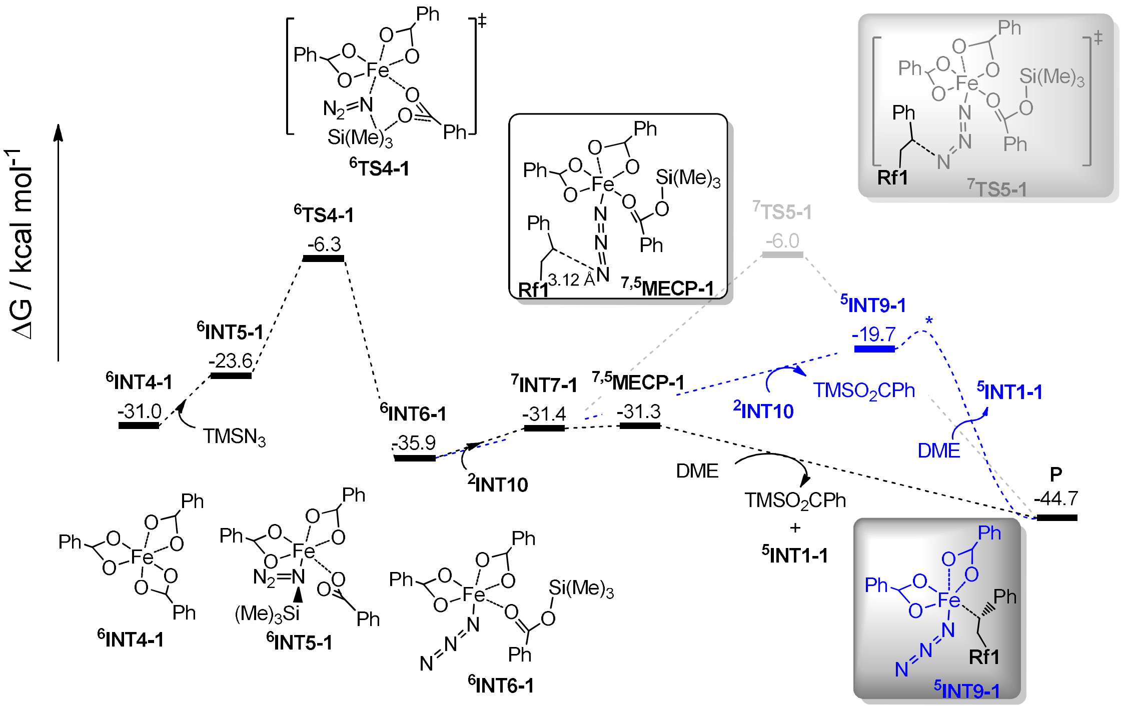

Figure S4. The Gibbs free energy profile of radical coupling azidation from the possible catalyst species, **⁶INT4-1**. Transition states corresponding to C-N bond coupling through inner-sphere pathway indicated by * were not explicitly located.

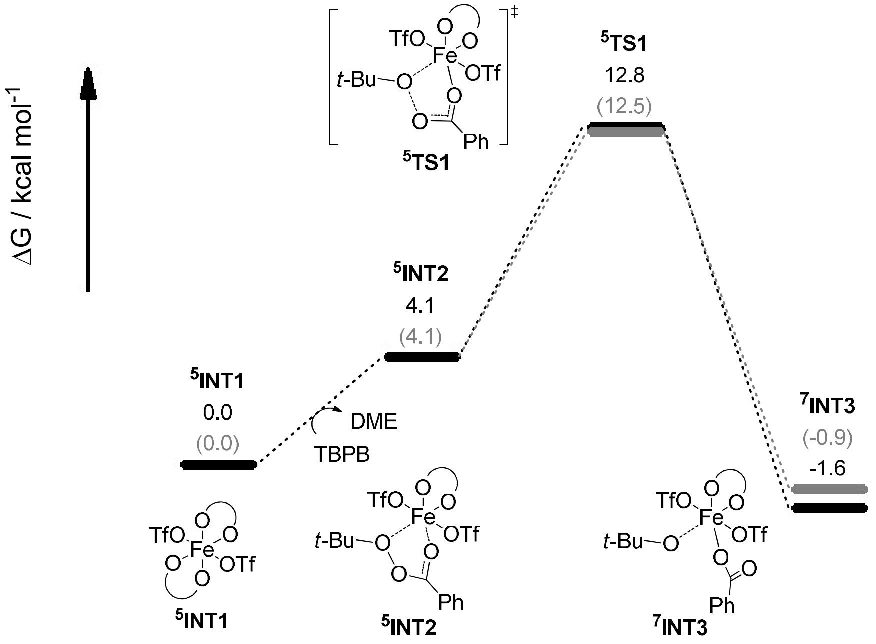

Figure S5. The Gibbs free energy profile from **⁵INT1** to **⁷INT3**. Energies in parenthesis (gray) are corrected by adding the solvation free energy correction.

Table S1. Relative free energy of iron catalyst coordinated by solvent molecule, DME, at singlet, triplet and quintet spin states.

	singlet	triplet	quintet
$Fe(OTf)_2$	71.5	56.7	39.0
$Fe(OTf)_2(DME)$	50.4	40.3	13.5[b]
$Fe(OTf)_2(DME)_2$	35.7	31.9	0.0[a]

[a]A most possible catalyst species **^{5}INT1** in the Fe(II)-catalyzed reaction. [b]Named **^{5}INT12′**.

Table S2. Relative free energy of $Fe(OTf)_2(DME)_2$, **^{5}INT1**, calculated at singlet, triplet and quintet spin states by several functionals.

DFT\spin state	singlet	triplet	quintet
B3LYP	35.7	31.9	0.0
M06-L	36.3	31.8	0.0
M06	51.0	47.2	0.0
wB97XD	36.7	33.7	0.0
PBE1PBE	46.3	38.4	0.0

Table S3. Relative free energy of several possible iron(II) species ligated by different anions or solvent molecule, DME, at quintet spin states.

	iron(II) species	E_{sol}	G_{298} corr.	ΔG_{298}
**^{5}INT1**	$Fe(OTf)_2(DME)_2$	-3805.499999	0.278698	0.0
**^{5}INT1-1**	$Fe(OBz)_2(DME)$	-2413.707752	0.293427	-35.3
**^{5}INT1-2**	$Fe(OBz)_2(DME)_2$	-2722.728123	0.426532	-32.1
**^{5}INT1-3**	$Fe(OTf)(OBz)(DME)_2$	-3264.114964	0.353745	-15.9
**^{5}INT1-4**	$[Fe(OBz)(DME)_2]^+$	-2302.154827	0.333872	-13.5
**^{5}INT1-5**	$[Fe(OBz)(DME)_3]^+$	-2611.167299	0.467809	-4.8
**^{5}INT1-6**	$[Fe(OTf)(DME)_2]^+$	-2843.518318	0.256916	14.7
**^{5}INT1-7**	$[Fe(OTf)(DME)_3]^+$	-3152.548479	0.390881	12.3

Table S4. Electronic potential energies and correction to zero point energies, thermal energies, enthalpies, free energies (in Hartree) and imaginary frequencies (cm^{-1}) of optimized structures calculated at the B3LYP-D3/Def2-TZVP-(SMD-DME)//B3LYP-D3/Def2-SVP.

Entry	Structure	$E_{0,sol}$	$E_{0,gas}$	$cZPE_{gas}$	$cU_{298,gas}$	$cH_{298,gas}$	$cG_{298,gas}$	Imaginary Frequency
1	**^{5}INT1**	-3805.500021	-3802.856243	0.347970	0.382490	0.383434	0.278728	
2	**^{5}INT2**	-4149.983598	-4146.964551	0.435006	0.476616	0.477560	0.354166	
3	**^{5}TS1**	-4149.970470	-4146.952296	0.433407	0.474348	0.475292	0.354894	-511.2694
4	**^{7}INT3**	-4149.990928	-4146.969698	0.432654	0.474534	0.475478	0.352434	
5	**^{7}TS2**	-4149.980161	-4146.954134	0.429574	0.471530	0.472474	0.349938	-265.9877
6	**^{6}INT4**	-3916.853890	-3914.083945	0.308966	0.343143	0.344087	0.236497	
7	**^{6}INT5**	-4490.537644	-4487.349208	0.435293	0.480705	0.481649	0.352141	
8	**^{6}TS4**	-4490.513576	-4487.329479	0.435287	0.479634	0.480579	0.353885	-131.5157
9	**^{6}INT6**	-4490.549692	-4487.360850	0.435082	0.480612	0.481556	0.350644	
10	**^{7}INT7**	-5736.352476	-5732.106360	0.596037	0.657175	0.658119	0.489480	
11	**^{7}TS5**	-5736.311086	-5732.067643	0.596833	0.656483	0.657427	0.495173	-621.5407
12	**^{6}INT8**	-3660.699979	-3658.215240	0.216513	0.245773	0.246717	0.151965	
13	**^{5}INT9**	-4906.515728	-4902.966471	0.379055	0.423956	0.424901	0.291911	
14	**^{7,5}MECP**	-5736.352339/-5736.353869						
15	**P**	-1410.087265	-1408.823752	0.176819	0.193081	0.194025	0.129835	
16	**^{7}TS2-OtBu**	-4149.980923	-4146.959216	0.432488	0.473562	0.474506	0.353075	-109.3829
17	OtBu	-233.114927	-232.841872	0.121746	0.128176	0.129121	0.092325	
18	**^{2}TS3**	-233.095161	-232.816626	0.117662	0.124663	0.125608	0.087378	-476.8338
19	CH_3	-39.860112	-39.809533	0.029384	0.032479	0.033423	0.009546	
20	**^{2}TS6**	-1273.674867	-1272.873191	0.057171	0.068562	0.069506	0.012747	-198.0746
21	**Rf1**	-935.949206	-935.209607	0.022908	0.029440	0.030384	-0.009503	
22	**^{2}INT10**	-1245.789428	-1244.717136	0.159910	0.173333	0.174278	0.117121	

23	**^{2}TS7**	-1273.659539	-1272.855278	0.057385	0.067838	0.068783	0.017548	-475.3052
24	**Rf2**	-773.524106	-772.931073	0.021719	0.028762	0.029706	-0.012714	
25	**^{2}TS8**	-349.637031	-349.247291	0.165667	0.175194	0.176138	0.129558	-292.5131
26	**^{2}INT11**	-349.693939	-349.310483	0.171613	0.180067	0.181011	0.137480	
27	**^{5}INT12**	-4459.009340	-4455.629675	0.580366	0.630097	0.631041	0.494298	
28	**^{5}INT12′**	-3496.448010	-3494.149640	0.202344	0.228638	0.229582	0.140637	
29	**^{7}INT9**	-4906.506241	-4902.956980	0.377160	0.422292	0.423236	0.290208	
30	DME	-309.000989	-308.636337	0.140717	0.148487	0.149431	0.108599	
31	TBPB	-653.496221	-652.754790	0.230287	0.243982	0.244926	0.189181	
32	acetone	-193.248617	-193.017719	0.083096	0.088483	0.089427	0.054696	
33	$TMSN_3$	-573.662402	-573.220853	0.123794	0.134205	0.135149	0.087685	
34	TMSOCOPh	-829.796891	-829.098643	0.216053	0.231013	0.231957	0.173562	
35	ClC_2F_4I	-1233.812422	-1233.059753	0.024615	0.032607	0.033551	-0.010586	
36	CH_3I	-337.738235	-337.675604	0.036197	0.039386	0.040331	0.010417	
37	CH_3Cl	-500.162126	-499.947745	0.037348	0.040377	0.041321	0.013669	
38	styrene	-309.779660	-309.437874	0.133350	0.140067	0.141011	0.102055	
39	**^{5}INT1-1**	-2413.707752	-2412.240648	0.354183	0.379746	0.380690	0.293427	
40	**^{5}INT1-2**	-2722.728123	-2720.911936	0.498079	0.532710	0.533654	0.426532	
41	**^{5}INT1-3**	-3264.114964	-3261.884626	0.423250	0.457600	0.458544	0.353745	
42	**^{5}INT1-4**	-2302.154827	-2300.759720	0.393624	0.419812	0.420757	0.333872	
43	**^{5}INT2-1**	-2758.192708	-2756.345716	0.442113	0.474264	0.475208	0.371437	
44	**^{5}TS1-1**	-2758.188152	-2756.340752	0.440102	0.471992	0.472936	0.369842	-585.8323
45	**^{7}INT3-1**	-2758.219172	-2756.372150	0.440325	0.472670	0.473614	0.369903	
46	**^{7}TS2-1**	-2758.207342	-2756.354008	0.436762	0.469602	0.470546	0.364337	-331.6695
47	**^{6}INT4-1**	-2525.086878	-2523.494493	0.315606	0.340513	0.341457	0.253047	
48	**^{5}INT2-2**	-3067.210805	-3065.015857	0.585853	0.627018	0.627962	0.505237	
49	**^{5}TS1-2**	-3067.203325	-3065.008176	0.584122	0.624804	0.625748	0.504682	-619.1538

50	^{7}**INT3-2**	-3067.235237	-3065.035657	0.584465	0.625659	0.626604	0.503757	
51	^{7}**TS2-2**	-3067.220305	-3065.013873	0.579877	0.621871	0.622815	0.496224	-359.2748
52	^{6}**INT4-2**	-2834.105784	-2832.164158	0.460509	0.494209	0.495154	0.387528	
53	^{5}**INT2-3**	-3608.597578	-3605.987557	0.509941	0.551342	0.552286	0.429368	
54	^{5}**TS1-3**	-3608.588924	-3605.978609	0.508262	0.549099	0.550044	0.428571	-586.1345
55	^{7}**INT3-3**	-3608.615171	-3606.007194	0.509411	0.550485	0.551429	0.430958	
56	^{7}**TS2-3**	-3608.602848	-3605.987840	0.505443	0.547033	0.547977	0.425787	-335.5835
57	^{6}**INT4-3**	-3375.476674	-3373.122750	0.384273	0.418123	0.419067	0.311429	
58	^{5}**INT2-4**	-2646.639994	-2644.872389	0.481485	0.514146	0.515091	0.412320	
59	^{5}**TS1-4**	-2646.632287	-2644.865127	0.479861	0.511971	0.512915	0.412558	-555.6746
60	^{7}**INT3-4**	-2646.654420	-2644.883612	0.479781	0.512592	0.513537	0.410124	
61	^{7}**TS2-4**	-2646.646019	-2644.869556	0.476362	0.509344	0.510288	0.408029	-291.4152
62	^{6}**INT4-4**	-2413.515472	-2411.997662	0.357192	0.382605	0.383549	0.295421	
63	^{6}**INT5-1**	-3098.762949	-3096.746619	0.443075	0.479424	0.480369	0.366119	
64	^{6}**TS4-1**	-3098.736980	-3096.723065	0.443547	0.479021	0.479965	0.367753	-151.9294
65	^{6}**INT6-1**	-3098.782263	-3096.764795	0.442177	0.478648	0.479592	0.365932	
66	^{7}**INT7-1**	-4344.589570	-4341.508407	0.604299	0.655872	0.656816	0.508024	
67	^{7,5}**MECP-1**	-4344.589280/-4344.589227						
68	^{7}**TS5-1**	-4344.550568	-4341.471060	0.603732	0.654377	0.655321	0.509492	-611.6531
69	^{5}**INT9-1**	-3514.748704	-3512.373409	0.386048	0.421413	0.422358	0.309254	
70	OBz^{-}	-420.521421	-419.963543	0.102187	0.109050	0.109994	0.070331	
71	OTf^{-}	-961.931056	-960.954704	0.027229	0.034443	0.035387	-0.005432	

Entry	Structure	$E_{0,sol}$(Def2-TZVP)	$E_{0,sol}$	$cZPE_{298,sol}$	$cU_{298,sol}$	$cH_{298,sol}$	$cG_{298,sol}$	Imaginary Frequency
72	^{5}**INT1**	-3805.511232	-3802.884399	0.347189	0.381831	0.382775	0.277729	
73	^{5}**INT2**	-4150.002648	-4147.000084	0.433807	0.475662	0.476606	0.352967	
74	^{5}**TS1**	-4149.989567	-4146.987379	0.432241	0.473368	0.474312	0.353196	-526.8048

75	^{7}**INT3**	-4150.008670	-4147.006241	0.431588	0.473657	0.474601	0.350929
76	DME	-309.005617	-308.645515	0.140069	0.147848	0.148792	0.107971
77	TBPB	-653.509349	-652.775424	0.229948	0.243652	0.244596	0.188956

Coordinate of optimized structures

Structure S1. ^{5}INT1

E(B3LYP)$_{sol}$ = -3805.50002075 E(B3LYP) = -3802.85624325

26	-0.057950	-0.051635	0.402634
8	3.455478	-1.607099	0.284599
16	3.006057	-0.438503	-0.482905
8	2.750954	-0.593475	-1.919357
8	1.915514	0.357891	0.234449
6	4.389995	0.810513	-0.355159
9	5.466713	0.369445	-0.996772
9	4.004276	1.968962	-0.902124
9	4.701225	1.025028	0.922902
8	-0.443648	1.624556	1.770091
8	-0.330743	1.661219	-0.956489
6	-0.729140	2.824091	1.047585
1	-1.803579	2.848274	0.794871
1	-0.475381	3.706176	1.662601
6	0.081998	2.810651	-0.229749
1	-0.131629	3.728012	-0.807742
1	1.165400	2.755789	-0.021441
6	-1.349568	1.364101	2.841744
1	-1.071970	0.391023	3.265014
1	-1.268371	2.150626	3.611370
1	-2.382629	1.296563	2.465632
6	0.172343	1.556136	-2.288977
1	1.249797	1.330055	-2.279697
1	-0.389554	0.738403	-2.754534
1	-0.023850	2.491066	-2.840994
8	0.177473	-1.958694	-0.651221
8	0.456952	-1.468701	1.987645
6	-0.209476	-2.985025	0.266182
1	-1.288464	-2.899612	0.478177
1	-0.006125	-3.978761	-0.167316
6	0.644590	-2.804918	1.502893
1	0.355539	-3.531167	2.283632
1	1.704996	-2.938783	1.234498
6	0.005527	-2.250543	-2.040256
1	0.499585	-1.439856	-2.585641
1	0.514754	-3.197650	-2.283427
1	-1.062155	-2.299358	-2.298711
6	1.457033	-1.050363	2.920813
1	2.456850	-1.129861	2.468135
1	1.253838	0.002492	3.153409
1	1.396442	-1.658118	3.839794
8	-3.592074	-2.020891	-0.514150
16	-3.002602	-0.688563	-0.644875
8	-2.041600	-0.367597	0.500536
8	-2.490333	-0.273638	-1.957635
6	-4.353495	0.540696	-0.238613
9	-4.812858	0.338228	0.997744
9	-5.357920	0.431262	-1.099689
9	-3.864180	1.788146	-0.302967

Optimized with SMD method

26	0.058035	0.057993	0.403465
8	-3.531377	1.589061	0.300033
16	-3.053605	0.438677	-0.480258

8	-2.851022	0.618312	-1.924079
8	-1.937059	-0.336489	0.201431
6	-4.414185	-0.835197	-0.338567
9	-5.523186	-0.386320	-0.926391
9	-4.045987	-1.974099	-0.933956
9	-4.681016	-1.091547	0.943133
8	0.464265	-1.604848	1.732340
8	0.345800	-1.626479	-0.973228
6	0.731208	-2.820541	1.022530
1	1.809496	-2.876511	0.795080
1	0.445020	-3.691258	1.637819
6	-0.058753	-2.797716	-0.263902
1	0.175533	-3.702463	-0.851515
1	-1.145979	-2.763059	-0.074535
6	1.338464	-1.380817	2.840100
1	1.102122	-0.390493	3.248609
1	1.182071	-2.151650	3.613553
1	2.390661	-1.385486	2.511905
6	-0.153137	-1.520395	-2.305322
1	-1.235133	-1.312497	-2.301918
1	0.392816	-0.692314	-2.772623
1	0.052291	-2.448825	-2.865020
8	-0.222230	1.955724	-0.655057
8	-0.455132	1.463962	1.975847
6	0.165556	2.994901	0.251760
1	1.249456	2.926807	0.443466
1	-0.059820	3.983733	-0.180251
6	-0.653388	2.806257	1.507074
1	-0.343419	3.527501	2.283096
1	-1.720632	2.946690	1.272291
6	-0.038753	2.237687	-2.041760
1	-0.506092	1.415164	-2.593325
1	-0.554239	3.176575	-2.304710
1	1.030681	2.305367	-2.292619
6	-1.421668	1.048341	2.945715
1	-2.438030	1.123162	2.528916
1	-1.211379	-0.001150	3.187040
1	-1.335763	1.662266	3.858107
8	3.727312	2.005581	-0.480575
16	3.064819	0.707063	-0.640927
8	2.058984	0.426059	0.463104
8	2.594217	0.332679	-1.982213
6	4.365782	-0.570135	-0.218137
9	4.786555	-0.414607	1.039549
9	5.409403	-0.445994	-1.037741
9	3.867030	-1.804546	-0.345957

Structure S2. [5]INT2
E(B3LYP)$_{sol}$ = -4149.98359847 E(B3LYP) = -4146.96455106

26	-0.099905	-0.890376	-0.498423
8	-2.859494	1.531120	0.141376
16	-3.142834	0.191424	-0.382090
8	-3.612938	0.059293	-1.766580
8	-2.060645	-0.831268	-0.033306
6	-4.535116	-0.500179	0.658044
9	-5.616199	0.262945	0.534557
9	-4.826478	-1.738087	0.258209
9	-4.180075	-0.540551	1.946384
8	2.814876	-1.488422	1.745099
16	2.932401	-0.716458	0.497702

8	1.885666	-1.125644	-0.540076
8	3.123581	0.733683	0.582920
6	4.458130	-1.397835	-0.340243
9	4.323863	-2.714551	-0.528403
9	5.523349	-1.180607	0.426494
9	4.644301	-0.812173	-1.520120
8	-0.195182	-3.108564	-0.307532
8	-0.578538	-1.579429	-2.479365
6	-0.591154	-3.731000	-1.526462
1	0.310700	-4.026895	-2.093681
1	-1.191174	-4.635777	-1.317788
6	-1.395199	-2.742959	-2.346064
1	-1.607683	-3.178490	-3.338217
1	-2.343450	-2.469440	-1.855430
6	0.842201	-3.810505	0.377498
1	1.103859	-3.231974	1.268583
1	0.495658	-4.819174	0.660666
1	1.744597	-3.877279	-0.252390
6	-0.987810	-0.638876	-3.479246
1	-2.011154	-0.289359	-3.278851
1	-0.290045	0.204607	-3.410462
1	-0.918574	-1.104131	-4.477180
8	0.020273	1.179564	-1.029387
6	0.323131	2.027292	-0.200119
8	0.406837	1.790771	1.108761
8	0.195471	0.399288	1.404329
6	-0.137254	0.270941	2.838176
6	-0.393229	-1.232478	2.930178
1	0.519173	-1.779197	2.658589
1	-0.657587	-1.483861	3.967687
1	-1.223095	-1.524192	2.270520
6	1.078144	0.700558	3.654063
1	1.965071	0.135195	3.339647
1	1.274170	1.774321	3.523533
1	0.884567	0.513672	4.721394
6	-1.388101	1.087627	3.139713
1	-1.189666	2.164118	3.044190
1	-2.197578	0.825993	2.449862
1	-1.710469	0.884741	4.172111
6	0.625671	3.435246	-0.518252
6	-0.019532	4.015743	-1.621535
6	1.549747	4.169212	0.242735
6	0.251084	5.342411	-1.953343
1	-0.737857	3.422301	-2.190224
6	1.823565	5.491933	-0.106574
1	2.065364	3.687032	1.074736
6	1.172292	6.078474	-1.197849
1	-0.256592	5.805776	-2.802425
1	2.551895	6.066260	0.470485
1	1.386808	7.116535	-1.464479

Optimized with SMD method

26	0.098779	-0.945633	-0.472020
8	-3.008722	1.134516	0.249937
16	-3.083163	-0.201153	-0.354581
8	-3.515481	-0.302026	-1.754831
8	-1.886613	-1.083732	-0.027666
6	-4.403599	-1.124110	0.597362
9	-5.570194	-0.492116	0.475126
9	-4.530786	-2.362123	0.116503
9	-4.093008	-1.201278	1.894065

8	3.224975	-0.932060	1.741657
16	3.108814	-0.227733	0.454335
8	2.114290	-0.885449	-0.493945
8	3.030666	1.236612	0.464540
6	4.694359	-0.642809	-0.445285
9	4.843145	-1.966237	-0.534151
9	5.732896	-0.139163	0.222575
9	4.684628	-0.128511	-1.674909
8	0.306272	-3.112096	-0.220935
8	-0.260274	-1.741103	-2.425442
6	-0.039823	-3.852274	-1.396730
1	0.884327	-4.102403	-1.947565
1	-0.554809	-4.790088	-1.123839
6	-0.934221	-2.995340	-2.264157
1	-1.073324	-3.486105	-3.241986
1	-1.919079	-2.820881	-1.801503
6	1.383492	-3.689629	0.518273
1	1.718853	-2.954176	1.256066
1	1.051740	-4.614738	1.019859
1	2.234337	-3.913276	-0.147568
6	-0.769682	-0.896276	-3.463077
1	-1.820591	-0.636402	-3.267258
1	-0.158253	0.015183	-3.457730
1	-0.671676	-1.400605	-4.438976
8	-0.090996	1.114224	-1.007949
6	-0.032823	2.016014	-0.179653
8	0.100695	1.801873	1.130099
8	0.208670	0.399426	1.427614
6	-0.071398	0.206058	2.869299
6	0.015499	-1.314275	2.970996
1	1.028812	-1.652200	2.719279
1	-0.206735	-1.612464	4.006129
1	-0.716794	-1.793428	2.304062
6	1.025981	0.898875	3.669263
1	2.013773	0.561009	3.327706
1	0.963960	1.991583	3.561447
1	0.909116	0.653910	4.736042
6	-1.467290	0.723017	3.189241
1	-1.525265	1.814048	3.071898
1	-2.210790	0.265221	2.527687
1	-1.710930	0.474394	4.233301
6	-0.116657	3.451440	-0.508068
6	-0.644744	3.802897	-1.762560
6	0.313806	4.446993	0.387199
6	-0.746272	5.147504	-2.116526
1	-0.982867	3.016935	-2.440224
6	0.215133	5.789312	0.020771
1	0.737254	4.170268	1.353837
6	-0.315003	6.139709	-1.227091
1	-1.162734	5.424399	-3.087956
1	0.556563	6.565965	0.709233
1	-0.392085	7.193227	-1.508531

Structure S3. ⁵TS1

E(B3LYP)$_{sol}$ = -4149.97047007 E(B3LYP) = -4146.95229639

26	0.200380	-0.789890	-0.331856
8	-3.075680	0.838468	0.365354
16	-2.995181	-0.452919	-0.322989
8	-3.297799	-0.502997	-1.758230
8	-1.740147	-1.254715	0.043591

6	-4.278592	-1.558770	0.470556
9	-5.491416	-1.055735	0.266825
9	-4.215988	-2.777983	-0.068949
9	-4.067132	-1.661118	1.783970
8	3.352400	-0.696521	1.599263
16	3.162150	0.085362	0.367886
8	2.165090	-0.579551	-0.595284
8	2.984260	1.533397	0.467623
6	4.726195	-0.194971	-0.617988
9	4.903243	-1.503748	-0.825784
9	5.769407	0.277093	0.058528
9	4.654436	0.421169	-1.794466
8	0.646142	-2.976083	-0.249054
8	-0.105903	-1.550405	-2.341324
6	0.316495	-3.678635	-1.449103
1	1.238694	-3.820439	-2.040107
1	-0.106387	-4.669904	-1.205969
6	-0.668256	-2.858080	-2.257170
1	-0.768140	-3.300270	-3.263558
1	-1.660613	-2.802168	-1.784370
6	1.887653	-3.406074	0.319259
1	2.111440	-2.755410	1.167687
1	1.808364	-4.457095	0.644177
1	2.703152	-3.288711	-0.412372
6	-0.603353	-0.715239	-3.394445
1	-1.690937	-0.590612	-3.298832
1	-0.111896	0.257057	-3.274071
1	-0.335580	-1.156613	-4.369204
8	-0.279499	1.122034	-0.920538
6	-0.270751	1.999829	-0.040357
8	-0.014058	1.799757	1.208641

8	0.332574	0.133706	1.444702
6	0.065333	-0.084378	2.853442
6	0.398049	-1.580552	3.001334
1	1.466078	-1.746134	2.815707
1	0.161625	-1.879478	4.033954
1	-0.209142	-2.184561	2.311342
6	1.029527	0.772831	3.670464
1	2.056110	0.618852	3.311427
1	0.774895	1.836927	3.575915
1	0.965983	0.491476	4.732633
6	-1.404635	0.182345	3.160444
1	-1.661978	1.229743	2.958259
1	-2.050576	-0.450408	2.542377
1	-1.598660	-0.031824	4.222423
6	-0.610666	3.410191	-0.350270
6	-1.663804	3.643376	-1.248426
6	0.092116	4.471833	0.237427
6	-2.008761	4.957782	-1.561231
1	-2.209130	2.792553	-1.660983
6	-0.252040	5.782551	-0.097183
1	0.912074	4.258718	0.925950
6	-1.301596	6.024152	-0.991037
1	-2.833287	5.153034	-2.250946
1	0.299630	6.618130	0.339840
1	-1.572584	7.052233	-1.244747

Frequencies -- -511.2694

Red. masses -- 13.1573

Frc consts -- 2.0264

IR Inten -- 611.8932

Optimized with SMD method

26	0.105847	-0.836918	-0.341181
8	-3.065717	1.093072	0.421255
16	-3.079792	-0.155120	-0.348475
8	-3.400588	-0.082753	-1.779320
8	-1.900672	-1.074726	-0.038589
6	-4.455834	-1.193472	0.380134
9	-5.619762	-0.564410	0.225600
9	-4.516321	-2.372364	-0.242721
9	-4.254867	-1.408925	1.682000
8	3.310075	-0.856281	1.625711
16	3.151291	-0.119683	0.361763
8	2.109618	-0.743380	-0.567053
8	3.089053	1.343420	0.407174
6	4.690621	-0.539447	-0.613355
9	4.807628	-1.863089	-0.740035
9	5.764034	-0.073700	0.025381
9	4.641881	0.006445	-1.827572
8	0.392516	-3.003442	-0.221244
8	-0.212575	-1.581009	-2.338979
6	0.013783	-3.727552	-1.402226
1	0.928806	-3.983199	-1.963063
1	-0.509028	-4.659859	-1.127955
6	-0.873734	-2.848369	-2.253926
1	-0.975928	-3.297493	-3.255678
1	-1.873864	-2.707931	-1.815867
6	1.538744	-3.563283	0.431638
1	1.935592	-2.816099	1.123457
1	1.256548	-4.481414	0.973085
1	2.324549	-3.791662	-0.306684
6	-0.660364	-0.722704	-3.395237
1	-1.732154	-0.503747	-3.284462
1	-0.085931	0.207819	-3.311235
1	-0.458512	-1.198122	-4.369457
8	-0.157425	1.133523	-0.899892
6	-0.076131	1.995014	-0.005865
8	0.092809	1.739486	1.250333
8	0.276189	0.044250	1.460457
6	-0.029943	-0.172908	2.864927
6	0.186914	-1.690426	2.995506
1	1.240951	-1.941964	2.828734
1	-0.091485	-1.982252	4.019852
1	-0.449167	-2.242888	2.288931
6	0.978564	0.596644	3.713367
1	1.996661	0.390621	3.355251
1	0.792621	1.678221	3.659456
1	0.892345	0.282377	4.764646
6	-1.478715	0.198654	3.154678
1	-1.656940	1.264041	2.960055
1	-2.160250	-0.382469	2.523830
1	-1.701538	-0.009935	4.212210
6	-0.201839	3.442062	-0.310815
6	-0.991072	3.814532	-1.411782
6	0.444527	4.412345	0.471914
6	-1.130274	5.165962	-1.727470
1	-1.498352	3.042241	-1.993167
6	0.308696	5.761051	0.139223
1	1.062294	4.105311	1.317694
6	-0.478752	6.137195	-0.955815
1	-1.748329	5.464155	-2.577850
1	0.820128	6.521283	0.734572
1	-0.585773	7.194987	-1.210342

Frequencies -- -526.8048

Red. masses -- 13.2015

Frc consts -- 2.1586

IR Inten -- 1193.2012

Structure S4. [7]INT3

E(B3LYP)$_{sol}$ = -4149.99092806 E(B3LYP) = -4146.96969828

26	-0.132831	-0.769948	-0.303643
8	-2.970918	1.653707	0.603092
16	-3.071752	0.499863	-0.292515
8	-3.163074	0.704701	-1.738521
8	-2.083406	-0.627063	0.075375
6	-4.651392	-0.381426	0.188815
9	-5.693556	0.400091	-0.064570
9	-4.770809	-1.510155	-0.515339
9	-4.635172	-0.683803	1.486381
8	3.004406	-2.138432	1.674452
16	2.859748	-1.048319	0.709364
8	1.744525	-1.344457	-0.319704
8	2.803653	0.332807	1.201136
6	4.347965	-1.147921	-0.423992
9	4.362844	-2.331287	-1.035581
9	5.448586	-1.025390	0.314105
9	4.311992	-0.182897	-1.327033
8	-0.545739	-2.949854	-0.158903
8	-0.525925	-1.409397	-2.289843
6	-0.884318	-3.548868	-1.405875
1	0.030094	-3.952207	-1.877547
1	-1.603791	-4.372755	-1.251241
6	-1.479701	-2.473946	-2.290399
1	-1.612778	-2.860350	-3.314306
1	-2.443839	-2.104640	-1.907156
6	0.303961	-3.754417	0.667392
1	-0.197267	-4.710894	0.889774
1	1.272014	-3.924844	0.170871
1	0.483925	-3.189433	1.586608
6	-0.605103	-0.456034	-3.368638
1	-1.510821	0.156006	-3.257235
1	0.298451	0.159467	-3.291661
1	-0.608261	-1.003925	-4.324348
8	0.066311	1.007687	-0.765507
6	1.041229	1.787212	-1.244716
8	1.892214	1.372153	-2.003932
8	0.042900	-0.856212	1.789116
6	-0.089909	0.025491	2.835588
6	-1.549501	-0.306896	3.363890
1	-1.627660	-1.368774	3.630337
1	-1.699379	0.320679	4.254612
1	-2.281591	-0.054525	2.590167
6	0.937016	-0.363819	3.920376
1	0.836044	-1.423645	4.192906
1	1.945101	-0.197012	3.518523
1	0.780804	0.262596	4.810803
6	-0.005931	1.487154	2.411059
1	0.977914	1.677694	1.962050
1	-0.785637	1.713237	1.674773
1	-0.140735	2.144230	3.282585
6	0.957659	3.202786	-0.780604
6	-0.235452	3.731410	-0.261299
6	2.098545	4.012653	-0.891400

6	-0.278388	5.065805	0.148690
1	-1.123829	3.101626	-0.192310
6	2.053436	5.340187	-0.466058
1	3.009903	3.577424	-1.306267
6	0.864439	5.867645	0.053685
1	-1.208709	5.480081	0.545117
1	2.944915	5.968111	-0.540078
1	0.828922	6.909536	0.383302

Optimized with SMD method

26	-0.131167	-0.816196	-0.299168
8	-3.019980	1.581709	0.723353
16	-3.088193	0.492100	-0.256689
8	-3.169846	0.825765	-1.679884
8	-2.097557	-0.645026	0.040873
6	-4.673307	-0.424833	0.133536
9	-5.709911	0.381317	-0.076902
9	-4.789217	-1.495150	-0.653492
9	-4.679317	-0.820936	1.405308
8	3.131576	-2.003690	1.701671
16	2.914962	-0.957003	0.700971
8	1.787566	-1.317119	-0.282123
8	2.854839	0.440145	1.146276
6	4.379979	-1.068906	-0.460134
9	4.446877	-2.291736	-0.986087
9	5.491348	-0.836952	0.238807
9	4.284331	-0.177212	-1.435089
8	-0.485913	-2.962365	-0.141541
8	-0.491167	-1.436972	-2.280855
6	-0.762838	-3.595292	-1.397705
1	0.181943	-3.962845	-1.835146
1	-1.447995	-4.446422	-1.248044
6	-1.387223	-2.560328	-2.299473
1	-1.475240	-2.954378	-3.323907
1	-2.378089	-2.238171	-1.941727
6	0.321237	-3.760319	0.736290
1	-0.172250	-4.730853	0.905343
1	1.323862	-3.909040	0.306066
1	0.411040	-3.212246	1.678756
6	-0.646180	-0.489304	-3.355471
1	-1.596297	0.051914	-3.244522
1	0.203502	0.198800	-3.287020
1	-0.610582	-1.030744	-4.313574
8	0.067170	0.978886	-0.743430
6	1.011976	1.778318	-1.229693
8	1.873630	1.392538	-1.996129
8	0.056403	-0.873276	1.793296
6	-0.084642	-0.012251	2.850827
6	-1.541211	-0.385228	3.377557
1	-1.597229	-1.452935	3.625668
1	-1.691830	0.225847	4.280017
1	-2.280220	-0.129932	2.611236
6	0.942861	-0.390594	3.935443
1	0.859093	-1.452743	4.205830
1	1.951165	-0.203195	3.541376
1	0.773472	0.228083	4.828692
6	-0.034041	1.455874	2.445642
1	0.950847	1.675654	2.011708
1	-0.807883	1.668907	1.699697
1	-0.197188	2.098358	3.323120
6	0.894620	3.198012	-0.774617

6	-0.310912	3.700896	-0.257538
6	2.014449	4.037626	-0.886214
6	-0.389791	5.036386	0.145437
1	-1.181288	3.047441	-0.183981
6	1.935653	5.366683	-0.467144
1	2.940673	3.629155	-1.296196
6	0.732801	5.867449	0.048599
1	-1.330851	5.429046	0.539481
1	2.811802	6.016081	-0.542892
1	0.670199	6.909871	0.372848

Structure S5. ^{7}TS2

E(B3LYP)$_{sol}$ = -4149.98016067 E(B3LYP) = -4146.95413418

26	-0.207976	-0.782916	-0.277230
8	-2.876817	1.967558	-0.123600
16	-3.141281	0.603095	-0.585778
8	-3.430634	0.364637	-2.002733
8	-2.164387	-0.434733	-0.013499
6	-4.666403	0.049364	0.344257
9	-5.697766	0.822780	0.027606
9	-4.956029	-1.214635	0.036920
9	-4.451504	0.133219	1.662164
8	2.825634	-1.964901	1.869639
16	2.747083	-1.045244	0.731882
8	1.644341	-1.459533	-0.262862
8	2.751161	0.401164	0.992298
6	4.255353	-1.391034	-0.320010
9	4.231407	-2.658233	-0.731545
9	5.343301	-1.195883	0.422740
9	4.288645	-0.586053	-1.369090
8	-0.740720	-2.921439	-0.038442
8	-0.614310	-1.506059	-2.246274
6	-1.071501	-3.577613	-1.258406
1	-0.161257	-4.036110	-1.685750
1	-1.822027	-4.367332	-1.075415
6	-1.609988	-2.530909	-2.209235
1	-1.740012	-2.966408	-3.213780
1	-2.565810	-2.102703	-1.869408
6	0.051674	-3.706678	0.858185
1	-0.491953	-4.629776	1.119402
1	1.026435	-3.941320	0.402165
1	0.221204	-3.092849	1.747718
6	-0.642592	-0.611682	-3.375878
1	-1.529916	0.033973	-3.316655
1	0.283340	-0.028452	-3.321262
1	-0.656607	-1.211013	-4.300365
8	0.147710	0.939869	-0.858628
6	1.205795	1.585908	-1.355652
8	1.973265	1.078194	-2.146571
8	-0.204675	-0.776934	1.781739
6	-0.498706	0.099700	2.658954
6	-1.455323	-0.362185	3.740537
1	-1.214044	-1.380867	4.072237
1	-1.496734	0.323708	4.596618
1	-2.448267	-0.383534	3.256521
6	1.275476	0.086189	3.792119
1	1.428299	-0.990439	3.887043
1	1.930423	0.587666	3.077461
1	0.946901	0.640097	4.675013
6	-0.521179	1.551343	2.263331

1	0.323668	1.778950	1.603003
1	-1.445273	1.711723	1.680371
1	-0.527799	2.222102	3.132336
6	1.326762	2.990042	-0.864243
6	0.208065	3.686702	-0.379188
6	2.582691	3.612846	-0.910378
6	0.353759	5.005671	0.058149
1	-0.766496	3.194034	-0.360772
6	2.724727	4.923733	-0.455384
1	3.432635	3.045679	-1.294828
6	1.609960	5.621515	0.027181
1	-0.517521	5.554745	0.424304
1	3.705144	5.406390	-0.477248
1	1.721764	6.651116	0.377880

Frequencies -- -265.9877

Red. masses -- 6.8162

Frc consts -- 0.2841

IR Inten -- 15.9697

Structure S6. ⁶INT4

E(B3LYP)$_{sol}$ = -3916.85389041 E(B3LYP) = -3914.08394546

26	-0.058289	-0.651363	0.235248
8	-3.549464	-1.622188	-2.112648
16	-3.113860	-1.017631	-0.862063
8	-3.478758	-1.655214	0.411729
8	-1.605223	-0.672270	-0.909622
6	-3.829166	0.711587	-0.764326
9	-5.147731	0.622741	-0.618307
9	-3.314680	1.325928	0.304251
9	-3.546673	1.396738	-1.855222
8	-1.088932	3.379080	1.203365
16	-0.354184	2.550176	0.259965
8	-0.101783	1.135003	0.882440
8	-0.743878	2.459630	-1.141120
6	1.424080	3.150277	0.272204
9	1.859766	3.279238	1.520401
9	1.498206	4.324352	-0.344130
9	2.199555	2.271241	-0.369429
8	-0.932905	-1.316634	1.996822
8	-0.108378	-2.869125	0.032058
6	-0.730169	-2.701742	2.300528
1	0.300321	-2.813835	2.673701
1	-1.445473	-3.029741	3.069249
6	-0.936476	-3.485944	1.020052
1	-0.636276	-4.539893	1.158153
1	-1.991475	-3.430093	0.707666
6	-1.842339	-0.552640	2.807147
1	-2.861086	-0.941958	2.674636
1	-1.519661	-0.594265	3.858964
1	-1.782020	0.478456	2.441203
6	-0.262156	-3.383136	-1.296297
1	-1.293219	-3.237515	-1.656079
1	0.427927	-2.810991	-1.927997
1	0.004551	-4.452580	-1.312018
8	1.507440	-0.676668	-1.019193
6	2.353764	-0.873393	-0.064583
8	1.882340	-1.047005	1.093358
6	3.802443	-0.873890	-0.343141
6	4.268065	-0.595546	-1.638706
6	4.710573	-1.136749	0.695474

6	5.639603	-0.585792	-1.892408
1	3.544039	-0.382285	-2.427080
6	6.080454	-1.127903	0.435228
1	4.324097	-1.340587	1.695797
6	6.544040	-0.853060	-0.857507
1	6.007048	-0.366181	-2.897569
1	6.791064	-1.331525	1.239778
1	7.618423	-0.843718	-1.058869

Structure S7. [6]INT5

E(B3LYP)$_{sol}$ = -4490.53764364 E(B3LYP) = -4487.34920844

26	0.152589	-0.638283	-0.411108
8	-3.076657	0.257234	0.997794
16	-3.011138	-0.519846	-0.245406
8	-3.348161	0.118265	-1.516000
8	-1.717138	-1.362595	-0.322098
6	-4.220269	-1.934129	-0.043980
9	-5.460423	-1.459022	-0.034948
9	-4.086586	-2.785160	-1.064683
9	-3.990276	-2.584731	1.092721
8	3.648843	-2.170871	-0.606978
16	3.366704	-0.905988	0.076980
8	2.069711	-0.242123	-0.416202
8	3.475206	-0.854897	1.538419
6	4.609614	0.325698	-0.589675
9	4.516966	0.396321	-1.910082
9	5.830913	-0.075665	-0.251191
9	4.385987	1.529784	-0.065906
8	0.667704	-2.766830	-0.005807
8	0.172950	-1.568394	-2.341208
6	0.803123	-3.542031	-1.201061
1	1.851945	-3.484820	-1.535677
1	0.528837	-4.592360	-1.000985
6	-0.133161	-2.961358	-2.233688
1	0.035497	-3.450706	-3.208041
1	-1.187713	-3.077027	-1.934020
6	1.327804	-3.335766	1.131275
1	0.713418	-4.152364	1.544866
1	2.325950	-3.698279	0.847592
1	1.459989	-2.537102	1.868790
6	-0.575256	-0.869874	-3.358527
1	-1.631542	-0.789549	-3.057762
1	-0.125415	0.125774	-3.441922
1	-0.475026	-1.415132	-4.310112
8	-0.405025	1.044937	-0.907645
6	-0.149268	2.138335	-1.623858
8	0.562286	2.120904	-2.607760
6	-0.813093	3.371696	-1.102388
6	-1.857089	3.297952	-0.165600
6	-0.377195	4.620163	-1.573431
6	-2.450598	4.472200	0.302240
1	-2.211616	2.325024	0.174110
6	-0.966325	5.790072	-1.093771
1	0.426537	4.648984	-2.311957
6	-2.002789	5.716774	-0.154470
1	-3.269461	4.413253	1.023515
1	-0.620906	6.762567	-1.453461
1	-2.466543	6.634187	0.217844
7	-0.026445	-0.422016	1.745886
7	-0.796272	-1.229511	2.278361

14	0.514954	1.029331	2.785830
7	-1.487241	-2.001126	2.728272
6	1.590796	0.268632	4.114096
6	1.436411	2.198596	1.675089
6	-1.097819	1.729316	3.428954
1	1.924937	1.040136	4.827885
1	1.049379	-0.503073	4.685849
1	2.477657	-0.186307	3.646358
1	2.281952	1.704785	1.182575
1	0.776501	2.631327	0.912457
1	1.827322	3.021913	2.296368
1	-1.766713	1.966187	2.587574
1	-1.621406	1.013575	4.083189
1	-0.915902	2.649228	4.008781

Structure S8. [6]TS4

E(B3LYP)$_{sol}$ = -4490.51357594 E(B3LYP) = -4487.32947868

26	0.239932	-0.844169	0.037612
8	-3.065292	0.402483	0.551521
16	-2.907481	-0.723972	-0.366491
8	-3.114119	-0.522094	-1.804340
8	-1.621553	-1.537101	-0.075892
6	-4.154311	-2.023690	0.139880
9	-5.382069	-1.556163	-0.053835
9	-3.981066	-3.119704	-0.600632
9	-3.997503	-2.335518	1.421685
8	3.716197	-1.967772	-0.860955
16	3.566877	-0.619189	-0.303887
8	2.140178	-0.362196	0.210356
8	4.559819	-0.113312	0.635787
6	3.598041	0.544497	-1.774157
9	2.705624	0.150240	-2.687198
9	4.806941	0.540103	-2.320908
9	3.292828	1.780337	-1.385874
8	0.802308	-2.905222	0.332134
8	0.452717	-1.627681	-1.967539
6	1.035435	-3.638311	-0.879694
1	2.097632	-3.540056	-1.150706
1	0.772219	-4.699089	-0.726479
6	0.162458	-3.028843	-1.953050
1	0.412800	-3.472404	-2.931449
1	-0.910192	-3.170441	-1.740115
6	1.666227	-3.263017	1.417173
1	1.469018	-4.305230	1.716379
1	2.718883	-3.140799	1.121028
1	1.433522	-2.584058	2.245313
6	-0.124962	-0.906825	-3.068587
1	-1.220025	-1.008125	-3.052843
1	0.148557	0.143229	-2.927921
1	0.302779	-1.287880	-4.009490
8	-0.245687	0.916152	-0.634467
6	-0.427004	1.992981	0.042634
8	0.018563	2.181056	1.199119
6	-1.209024	3.075007	-0.609370
6	-2.042073	2.780452	-1.701003
6	-1.131714	4.386934	-0.115505
6	-2.786205	3.799453	-2.296052
1	-2.132332	1.750711	-2.047239
6	-1.866692	5.403959	-0.724254
1	-0.488993	4.593765	0.741944

6	-2.694476	5.110386	-1.814598
1	-3.447447	3.568233	-3.134547
1	-1.799303	6.427106	-0.346632
1	-3.276204	5.906652	-2.286488
7	-0.013966	-0.296252	1.962009
7	-1.005904	-0.593903	2.616815
14	1.185921	1.344943	2.663305
7	-1.909111	-0.879177	3.242854
6	2.250514	-0.004458	3.488266
6	2.528615	2.490626	1.992808
6	0.083973	2.092901	3.987796
1	2.930474	0.489399	4.204262
1	1.662065	-0.758699	4.034080
1	2.878739	-0.503059	2.736039
1	3.026468	2.018890	1.134469
1	2.143116	3.473025	1.686985
1	3.294873	2.622276	2.774638
1	-0.446995	2.980919	3.617832
1	-0.662802	1.382783	4.375897
1	0.723891	2.387470	4.836516

Frequencies -- -131.5157

Red. masses -- 7.4484

Frc consts -- 0.0759

IR Inten -- 9.8731

Structure S9. [6]INT6

E(B3LYP)$_{sol}$ = -4490.54969239 E(B3LYP) = -4487.36084973

26	-0.584317	-0.974223	0.312620
8	-2.817943	1.550377	1.251964
16	-3.108980	0.958953	-0.051873
8	-3.019012	1.800449	-1.252969
8	-2.422125	-0.407121	-0.255153
6	-4.881270	0.364037	0.005119
9	-5.694623	1.403852	0.159318
9	-5.185824	-0.263540	-1.131526
9	-5.046833	-0.477474	1.021918
8	2.405107	-3.571557	-0.603987
16	2.593557	-2.167558	-0.220195
8	1.319733	-1.548351	0.340222
8	3.763893	-1.798839	0.576402
6	2.833114	-1.257198	-1.858017
9	2.125111	-1.831963	-2.831084
9	4.118233	-1.293868	-2.187219
9	2.454862	0.023689	-1.755381
8	-1.046575	-3.025591	-0.172610
8	-0.399934	-1.079119	-1.930477
6	-0.794217	-3.371340	-1.541160
1	0.274918	-3.607968	-1.662726
1	-1.403912	-4.249411	-1.816278
6	-1.178631	-2.181790	-2.385501
1	-0.952135	-2.391670	-3.445596
1	-2.250439	-1.941020	-2.274875
6	-0.637257	-4.036353	0.761081
1	-1.211533	-4.958607	0.575171
1	0.441759	-4.226140	0.660054
1	-0.859764	-3.648861	1.761463
6	-0.563418	0.104446	-2.717280
1	-1.588149	0.493677	-2.625804
1	0.141868	0.845047	-2.330608
1	-0.315317	-0.120422	-3.767670

8	0.044671	0.956202	0.219651
6	1.078963	1.645048	0.252776
8	2.142186	1.305358	0.909939
6	1.140178	2.909371	-0.515051
6	-0.034748	3.415157	-1.101655
6	2.357533	3.598824	-0.657507
6	0.017407	4.606266	-1.825484
1	-0.979319	2.882503	-0.980703
6	2.401137	4.783519	-1.390701
1	3.260153	3.187885	-0.202877
6	1.231159	5.287959	-1.972834
1	-0.895869	5.003648	-2.274099
1	3.347262	5.316482	-1.510624
1	1.266906	6.219436	-2.543902
7	-1.073956	-1.134784	2.144453
7	-2.082483	-0.954066	2.787247
14	2.697026	0.988534	2.538912
7	-3.018922	-0.800159	3.425642
6	2.104952	-0.633012	3.230435
6	4.547009	1.034866	2.310408
6	1.987947	2.446455	3.484066
1	2.382101	-0.668909	4.298485
1	1.015271	-0.749557	3.146427
1	2.589744	-1.467924	2.708754
1	4.830820	0.235582	1.607638
1	4.887997	2.004461	1.914123
1	5.063145	0.850740	3.266953
1	2.302604	3.405183	3.041661
1	0.885553	2.409888	3.477340
1	2.317016	2.428764	4.536228

Structure S10. ^{7}**INT7**
E(B3LYP)$_{sol}$ = -5736.35247612 E(B3LYP) = -5732.10635985

26	-1.302531	-1.009679	-0.922914
8	1.691021	-1.451242	0.898368
16	0.993575	-2.701321	0.576715
8	0.466991	-3.503497	1.687239
8	-0.016293	-2.538574	-0.567462
6	2.245476	-3.797014	-0.282324
9	3.162505	-4.186311	0.602428
9	1.639965	-4.874086	-0.780292
9	2.837854	-3.131699	-1.266110
8	-4.906114	-0.289318	-2.474563
16	-4.319100	0.503591	-1.387544
8	-2.835683	0.210418	-1.192030
8	-4.588300	1.939278	-1.331143
6	-5.089259	-0.196410	0.188361
9	-5.352194	-1.496100	0.051242
9	-6.222740	0.446377	0.441088
9	-4.264249	-0.036915	1.230712
8	-1.938069	-2.036661	-2.713044
8	-2.755883	-2.545084	-0.207322
6	-3.005768	-2.972081	-2.508397
1	-3.965249	-2.431121	-2.534695
1	-2.981592	-3.737920	-3.302951
6	-2.794725	-3.608183	-1.156556
1	-3.637023	-4.285052	-0.930897
1	-1.844834	-4.168547	-1.114918
6	-2.063763	-1.273994	-3.922373
1	-2.018855	-1.953936	-4.788770

1	-3.011376	-0.715326	-3.917862
1	-1.218542	-0.577323	-3.945659
6	-2.759436	-2.979424	1.157801
1	-1.834603	-3.527570	1.390994
1	-2.816595	-2.078554	1.775531
1	-3.649877	-3.604189	1.335576
8	-1.116583	-0.470139	1.019513
6	-1.309757	0.529398	1.737101
8	-1.803142	1.622859	1.269757
6	-1.022979	0.442960	3.187825
6	-0.283843	-0.648256	3.679917
6	-1.488764	1.434818	4.069030
6	-0.004521	-0.730877	5.043800
1	0.077405	-1.412046	2.992394
6	-1.213461	1.339211	5.432127
1	-2.081212	2.264463	3.681569
6	-0.466929	0.258850	5.918754
1	0.578793	-1.572504	5.423985
1	-1.581702	2.105639	6.118012
1	-0.246841	0.188073	6.987144
7	0.033613	0.022748	-1.802228
7	1.180799	-0.156977	-2.126212
14	-1.636357	3.243124	0.649808
7	2.268125	-0.295309	-2.459466
6	-1.303744	3.142272	-1.176513
6	-3.303010	3.985071	1.044588
6	-0.237211	3.985238	1.650494
1	-1.148816	4.156496	-1.579106
1	-0.405563	2.546937	-1.388231
1	-2.166395	2.682553	-1.677596
1	-4.085382	3.412603	0.522743

1	-3.508820	3.980919	2.127028
1	-3.351733	5.028438	0.691064
1	-0.454811	3.951120	2.729403
1	0.711230	3.458930	1.467355
1	-0.085856	5.039028	1.365929
6	4.106679	0.544752	0.062446
6	5.189946	-0.357049	0.198814
6	4.150461	1.769787	-0.792944
6	5.005562	-1.566989	0.935894
6	6.472118	-0.138695	-0.388866
1	5.092492	2.330420	-0.687602
1	4.058127	1.499608	-1.861477
6	3.030126	2.742197	-0.474245
6	6.031975	-2.491480	1.066961
1	4.026907	-1.761929	1.375754
6	7.494881	-1.069371	-0.242823
1	6.658089	0.768623	-0.967842
6	2.939149	3.891746	-1.517735
9	3.198474	3.289804	0.754760
9	1.817221	2.114638	-0.465313
6	7.286877	-2.252086	0.483035
1	5.854493	-3.416409	1.621808
1	8.468051	-0.878167	-0.703356
9	4.143940	4.469122	-1.639514
9	2.605111	3.368334	-2.705902
17	1.742376	5.139133	-1.070252
1	8.093374	-2.981935	0.588426
1	3.148776	0.262623	0.500158

Structure S11. **^{7}TS5**

E(B3LYP)$_{sol}$ = -5736.31108567 E(B3LYP) = -5732.06764329

26	-0.857360	-0.768177	-0.977232
8	1.569224	-1.716626	1.394780
16	0.799532	-2.844301	0.859550
8	-0.010291	-3.634391	1.795830
8	0.039910	-2.500915	-0.422682
6	2.031588	-4.052310	0.131714
9	2.868552	-4.467971	1.083527
9	1.388692	-5.104962	-0.370148
9	2.729315	-3.466789	-0.837372
8	-3.838622	0.463336	-3.405342
16	-3.615335	0.988758	-2.052397
8	-2.279343	0.556653	-1.469071
8	-3.880568	2.404243	-1.794364
6	-4.869328	0.073783	-0.975735
9	-5.093436	-1.156552	-1.438695
9	-6.014931	0.744219	-0.976655
9	-4.432281	-0.021126	0.288544
8	-1.011780	-1.497313	-3.012473
8	-2.573830	-2.237556	-0.952935
6	-2.149314	-2.333438	-3.267171
1	-3.020053	-1.696778	-3.489081
1	-1.935796	-2.991377	-4.127551
6	-2.396758	-3.155872	-2.027218
1	-3.311247	-3.760103	-2.161550
1	-1.543291	-3.818955	-1.803650
6	-0.735952	-0.572876	-4.075797
1	-0.508643	-1.134765	-4.996508
1	-1.601222	0.088170	-4.231824
1	0.137505	0.012365	-3.771273
6	-2.945473	-2.855740	0.281127
1	-2.131605	-3.492034	0.659626
1	-3.142694	-2.050790	0.994416
1	-3.868081	-3.440772	0.133077
8	-1.188774	-0.290426	0.957524
6	-1.888233	0.427592	1.697531
8	-2.211291	1.646453	1.422544
6	-2.416615	-0.126277	2.966553
6	-1.936273	-1.363760	3.431770
6	-3.390363	0.575570	3.698816
6	-2.432632	-1.890517	4.624051
1	-1.175906	-1.903243	2.866660
6	-3.888522	0.036399	4.883811
1	-3.755900	1.531139	3.320182
6	-3.408481	-1.195177	5.347540
1	-2.053207	-2.848176	4.987605
1	-4.652472	0.575192	5.449262
1	-3.797684	-1.613856	6.279373
7	0.794135	0.123353	-1.282655
7	1.907436	-0.078231	-1.775263
14	-1.542209	3.186956	0.900236
7	3.092839	-0.089797	-1.735046
6	-0.722723	3.110465	-0.762928
6	-3.068892	4.258929	0.905008
6	-0.348996	3.569292	2.295507
1	-0.162132	4.046459	-0.915065
1	-0.011198	2.278218	-0.837968
1	-1.479586	3.008563	-1.549907
1	-3.780439	3.869365	0.160529
1	-3.550484	4.281608	1.895502

1	-2.811319	5.293275	0.622530
1	-0.877766	3.657337	3.257844
1	0.411250	2.778271	2.380689
1	0.186362	4.511843	2.098347
6	3.859253	0.319004	-0.030832
6	4.941362	-0.660295	0.104537
6	4.252042	1.772766	-0.151573
6	4.738526	-1.829024	0.869464
6	6.186633	-0.492015	-0.541388
1	4.897084	2.034463	0.704416
1	4.834199	1.967007	-1.063852
6	3.086523	2.747513	-0.157147
6	5.741093	-2.790519	0.983607
1	3.782742	-1.969893	1.372824
6	7.187373	-1.454900	-0.423079
1	6.370363	0.391084	-1.156875
6	3.558591	4.210733	0.081833
9	2.191091	2.444875	0.818988
9	2.430401	2.716444	-1.342681
6	6.970042	-2.609726	0.339316
1	5.555634	-3.690433	1.574820
1	8.142797	-1.308669	-0.933744
9	4.151378	4.289376	1.282058
9	4.466801	4.529309	-0.852295
17	2.216183	5.386216	0.018483
1	7.755243	-3.364969	0.427333
1	2.982546	0.124750	0.596815

Frequencies -- -621.5407

Red. masses -- 11.2972

Frc consts -- 2.5714

IR Inten -- 110.0862

Structure S12. [6]**INT8**

E(B3LYP)$_{sol}$ = -3660.69997936 E(B3LYP) = -3658.21523965

26	-0.289121	0.735968	0.159907
8	-3.773410	1.626371	-1.441282
16	-2.838974	0.743795	-0.775429
8	-1.553359	0.382735	-1.502660
8	-2.368965	1.120195	0.618455
6	-3.753236	-0.915852	-0.562504
9	-3.065019	-1.898519	-1.131843
9	-3.912038	-1.185461	0.732085
9	-4.941876	-0.824351	-1.135943
8	2.686353	-1.287350	1.263118
16	2.670020	-0.314059	0.160689
8	1.284561	-0.248839	-0.514127
8	3.238952	1.013208	0.392039
6	3.635723	-1.107841	-1.230271
9	3.089275	-2.282986	-1.535603
9	4.889184	-1.296802	-0.833903
9	3.622559	-0.324015	-2.300148
8	0.217754	0.619816	2.226220
8	-0.981436	-1.296911	0.785185
6	0.019616	-0.649578	2.872661
1	0.936289	-1.248243	2.755955
1	-0.187937	-0.485073	3.943963
6	-1.177956	-1.286914	2.199140
1	-1.347319	-2.307984	2.576945
1	-2.078187	-0.680927	2.368552
6	1.199270	1.460738	2.854312

1	0.892842	1.661994	3.893234
1	2.186974	0.979161	2.821068
1	1.226966	2.393637	2.282659
6	-0.372607	-2.468534	0.224270
1	-1.003266	-3.343678	0.447837
1	-0.332525	-2.301975	-0.857284
1	0.650048	-2.603293	0.605654
7	0.346716	2.464552	-0.122210
7	1.202306	3.175872	-0.585012
7	1.998021	3.875109	-1.017548

Structure S13. [5]INT9

E(B3LYP)$_{sol}$ = -4906.51572813 E(B3LYP) = -4902.96647099

26	1.038831	0.569509	-0.378767
8	-1.653821	2.478126	-1.656276
16	-1.431552	2.683510	-0.228544
8	-2.553618	2.550106	0.703485
8	-0.190390	1.935744	0.302410
6	-0.788050	4.431100	-0.028535
9	-1.678381	5.302428	-0.479093
9	-0.539838	4.681742	1.259730
9	0.351485	4.570636	-0.712751
8	4.528599	-1.377344	-0.730105
16	3.128294	-1.793093	-0.845394
8	2.213877	-0.957679	0.073711
8	2.553840	-2.014771	-2.170185
6	2.985812	-3.427022	0.055378
9	3.416125	-3.306863	1.312360
9	3.698227	-4.355547	-0.565665
9	1.701613	-3.806695	0.086156
8	2.833509	1.556830	-1.289034
8	2.244711	1.554994	1.360385
6	3.850364	1.892298	-0.344475
1	4.400334	0.978812	-0.062062
1	4.549864	2.621612	-0.789263
6	3.160507	2.511205	0.843021
1	3.907101	2.777280	1.613269
1	2.615817	3.426251	0.541828
6	3.340857	1.221615	-2.587646
1	3.784191	2.119319	-3.050176
1	4.089625	0.419311	-2.509892
1	2.492143	0.868619	-3.180801
6	1.603230	1.988145	2.556461
1	0.966207	2.865809	2.366111
1	0.980834	1.163406	2.909755
1	2.363061	2.223773	3.321488
7	0.400909	0.296745	-2.094820
7	-0.328953	0.187990	-3.040596
7	-1.021713	0.075149	-3.946048
6	-0.685715	-1.042286	0.438613
6	-0.535080	-0.851762	1.852686
6	-1.977851	-0.745838	-0.267016
6	0.468840	-1.577442	2.556200
6	-1.387911	0.017115	2.592570
1	-2.454016	0.184279	0.062266
1	-1.838193	-0.683824	-1.349892
6	-2.984183	-1.875642	-0.034720
6	0.577732	-1.475600	3.937323
1	1.145731	-2.221649	1.998347
6	-1.271380	0.104194	3.973431

1	-2.115001	0.643411	2.076689
6	-4.298564	-1.637596	-0.829389
9	-3.300990	-1.978057	1.278421
9	-2.459285	-3.060914	-0.428284
6	-0.296988	-0.644240	4.652832
1	1.343895	-2.049181	4.463671
1	-1.936801	0.768661	4.529102
9	-4.825732	-0.466971	-0.444495
9	-4.004606	-1.555551	-2.134627
17	-5.478002	-2.943192	-0.562853
1	-0.212289	-0.570059	5.739938
1	-0.076605	-1.830039	-0.012000

Structure S14. [7,5]**MECP**

E(B3LYP)$_{sol}$ = -5736.35233864; -5736.35386855

26	-1.275585	-1.026561	-0.914893
8	1.686619	-1.447004	0.944532
16	0.975330	-2.698759	0.661963
8	0.423954	-3.451099	1.795149
8	-0.017582	-2.565302	-0.500937
6	2.217552	-3.848366	-0.139386
9	3.123662	-4.210604	0.768291
9	1.598758	-4.938227	-0.591393
9	2.825661	-3.238533	-1.148924
8	-4.828249	-0.273285	-2.589699
16	-4.265910	0.506763	-1.480539
8	-2.791208	0.199077	-1.246684
8	-4.525652	1.944153	-1.420659
6	-5.086730	-0.198005	0.067549
9	-5.357320	-1.494215	-0.087099
9	-6.221392	0.453066	0.292524
9	-4.290870	-0.053737	1.134344
8	-1.862054	-2.061994	-2.716469
8	-2.759592	-2.546915	-0.230511
6	-2.949998	-2.979014	-2.537148
1	-3.899810	-2.423335	-2.592615
1	-2.916597	-3.748946	-3.327399
6	-2.785803	-3.612257	-1.177531
1	-3.642750	-4.277516	-0.973348
1	-1.844700	-4.184691	-1.107761
6	-1.941260	-1.303825	-3.932539
1	-1.881617	-1.988821	-4.794057
1	-2.880120	-0.731066	-3.958198
1	-1.085241	-0.619996	-3.934399
6	-2.791965	-2.978526	1.134886
1	-1.874677	-3.530656	1.387675
1	-2.855961	-2.076228	1.749813
1	-3.688755	-3.598663	1.296473
8	-1.142671	-0.467379	1.028471
6	-1.358491	0.543306	1.723587
8	-1.844698	1.625785	1.222709
6	-1.109124	0.482587	3.182432
6	-0.369755	-0.591854	3.709848
6	-1.611424	1.480597	4.036202
6	-0.125253	-0.650642	5.081648
1	0.016796	-1.362383	3.043728
6	-1.371041	1.408442	5.407350
1	-2.205249	2.295901	3.621285
6	-0.623208	0.345598	5.929293
1	0.458193	-1.479088	5.489577

1	-1.767797	2.179355	6.071981
1	-0.430476	0.293360	7.004020
7	0.102305	-0.012366	-1.750271
7	1.262370	-0.207549	-2.016431
14	-1.636237	3.245822	0.612563
7	2.363431	-0.360764	-2.292263
6	-1.261475	3.137417	-1.205267
6	-3.299922	4.014801	0.966360
6	-0.250015	3.964145	1.648052
1	-1.067496	4.146535	-1.603600
1	-0.375971	2.516220	-1.395379
1	-2.124545	2.701830	-1.726871
1	-4.078458	3.449121	0.431444
1	-3.529715	4.021442	2.043976
1	-3.326247	5.056186	0.604732
1	-0.491864	3.928067	2.721614
1	0.695344	3.426378	1.483178
1	-0.078179	5.016941	1.371882
6	4.111220	0.559097	0.077570
6	5.171615	-0.375779	0.158676
6	4.171779	1.799165	-0.755246
6	4.985992	-1.588589	0.890004
6	6.432225	-0.185606	-0.482949
1	5.115281	2.352890	-0.623897
1	4.097948	1.549267	-1.830011
6	3.051644	2.770917	-0.436903
6	5.991169	-2.542124	0.966205
1	4.022674	-1.762064	1.370473
6	7.434185	-1.145502	-0.392087
1	6.617554	0.722497	-1.060864
6	2.985334	3.942174	-1.457736
9	3.200699	3.293369	0.805436
9	1.836301	2.149042	-0.461370
6	7.225421	-2.330278	0.330048
1	5.812036	-3.468331	1.518186
1	8.391009	-0.975818	-0.893505
9	4.192233	4.522540	-1.535918
9	2.682551	3.444778	-2.665345
17	1.775702	5.178574	-1.014632
1	8.015158	-3.083068	0.392198
1	3.162862	0.301827	0.550084

Structure S15. **P**

E(B3LYP)$_{sol}$ = -1410.08726548 E(B3LYP) = -1408.82375202

7	1.776733	2.279152	0.172319
7	1.309431	3.364704	-0.156417
7	0.967187	4.422794	-0.399433
6	1.098191	1.067216	-0.340394
6	2.018496	-0.124134	-0.157450
6	-0.259714	0.915932	0.385978
6	2.064388	-1.127252	-1.134024
6	2.798636	-0.258433	0.999228
1	-0.099952	0.747098	1.459862
1	-0.833528	1.848147	0.268004
6	-1.125939	-0.215789	-0.145003
6	2.873690	-2.252249	-0.956482
1	1.450434	-1.034817	-2.033559
6	3.608606	-1.382603	1.176796
1	2.781403	0.531052	1.753824
6	-2.593811	-0.111400	0.359645

9	-0.647119	-1.422240	0.234332
9	-1.159793	-0.190537	-1.506218
6	3.647491	-2.383123	0.200150
1	2.900453	-3.027894	-1.725879
1	4.216424	-1.475502	2.080440
9	-2.584494	-0.094022	1.701855
9	-3.118635	1.049908	-0.064197
17	-3.597227	-1.461273	-0.215451
1	4.283069	-3.261357	0.338957
1	0.891296	1.191392	-1.416861

Structure S16. **[7]TS2-OtBu**

E(B3LYP)$_{sol}$ = -4149.98092273 E(B3LYP) = -4146.95921595

26	-0.369125	-0.527374	-0.410319
8	-2.879020	1.889792	1.445407
16	-2.959611	1.340569	0.092386
8	-2.710406	2.207379	-1.058291
8	-2.262194	-0.033496	-0.034234
6	-4.721863	0.736269	-0.095635
9	-5.556557	1.767321	-0.067862
9	-4.861469	0.099980	-1.267586
9	-5.031421	-0.107276	0.886254
8	2.482421	-2.989275	1.215273
16	2.440949	-1.674270	0.579703
8	1.291367	-1.577291	-0.447681
8	2.538439	-0.469225	1.411029
6	3.903655	-1.597626	-0.590249
9	3.779814	-2.522111	-1.538169
9	5.015457	-1.828151	0.102822
9	3.979755	-0.396889	-1.151972
8	-1.232711	-2.659258	-0.517382
8	-1.057772	-0.783400	-2.436551
6	-1.573928	-3.008887	-1.850005
1	-0.678507	-3.383475	-2.380390
1	-2.347596	-3.798570	-1.855191
6	-2.093337	-1.766677	-2.526626
1	-2.308835	-1.976209	-3.587860
1	-3.001343	-1.384808	-2.032632
6	-0.600546	-3.724456	0.199688
1	-1.277827	-4.594528	0.234349
1	0.357723	-3.993900	-0.270544
1	-0.397840	-3.356435	1.208279
6	-1.294356	0.377895	-3.258153
1	-2.202621	0.896488	-2.921716
1	-0.419775	1.022598	-3.136474
1	-1.382292	0.054036	-4.307585
8	0.240961	1.253172	0.047207
6	1.193676	1.537495	-0.801555
8	1.313730	0.852402	-1.824526
8	-0.350892	-1.096167	1.646880
6	-0.397769	-0.574009	2.916934
6	-1.924277	-0.780409	3.294534
1	-2.195065	-1.841839	3.222711
1	-2.028667	-0.432658	4.333042
1	-2.550178	-0.177298	2.629677
6	0.468348	-1.471154	3.828571
1	0.158363	-2.523193	3.753037
1	1.515557	-1.387807	3.509394
1	0.369151	-1.136424	4.871651
6	-0.020202	0.902312	3.000531

1	1.006715	1.026309	2.632388
1	-0.695993	1.508268	2.385755
1	-0.082630	1.239229	4.046161
6	2.100523	2.659623	-0.459438
6	1.843094	3.476502	0.652546
6	3.233978	2.887159	-1.255294
6	2.715726	4.520723	0.961841
1	0.958812	3.280881	1.260653
6	4.106838	3.927322	-0.938086
1	3.415956	2.229693	-2.107195
6	3.846968	4.745215	0.168746
1	2.516253	5.160392	1.825003
1	4.993933	4.102595	-1.551722
1	4.531792	5.560879	0.415583

Frequencies -- -109.3829

Red. masses -- 11.0850

Frc consts -- 0.0781

IR Inten -- 26.2577

Structure S17. OtBu

E(B3LYP)$_{sol}$ = -233.114926638 E(B3LYP) = -232.841872292

6	-0.000004	0.024920	0.084999
6	1.275110	0.792009	-0.316054
1	1.299634	1.768523	0.192135
1	2.167878	0.224901	-0.013311
1	1.310972	0.967155	-1.403023
6	0.000332	-1.388023	-0.577676
1	0.895715	-1.950952	-0.278577
1	-0.894787	-1.951380	-0.278591
1	0.000314	-1.258005	-1.670891
6	-1.275508	0.791390	-0.316031
1	-2.167985	0.223819	-0.013299
1	-1.300516	1.767876	0.192189
1	-1.311467	0.966549	-1.402997
8	0.000082	-0.260033	1.428117

Structure S18. ^{2}TS3

E(B3LYP)$_{sol}$ = -233.095161374 E(B3LYP) = -232.816626254

6	0.294412	0.000000	0.211647
6	0.716159	1.294063	-0.489105
1	0.245866	2.156766	0.002316
1	1.810114	1.393149	-0.375824
1	0.487824	1.298400	-1.564960
6	0.716158	-1.294063	-0.489105
1	1.810114	-1.393149	-0.375824
1	0.245866	-2.156766	0.002316
1	0.487824	-1.298400	-1.564960
6	-1.762303	0.000000	-0.327933
1	-2.092689	-0.925718	0.147104
1	-2.092689	0.925718	0.147105
1	-1.696195	0.000000	-1.419113
8	0.125926	0.000000	1.446102

Frequencies -- -476.8338

Red. masses -- 8.3439

Frc consts -- 1.1178

IR Inten -- 25.3796

Structure S19. CH_3

E(B3LYP)$_{sol}$ = -39.8601119636 E(B3LYP) = -39.8095333169

6	0.000000	-0.000026	0.000255
1	-0.938485	-0.556488	-0.000510
1	0.951282	-0.534327	-0.000510
1	-0.012796	1.090973	-0.000510

Structure S20. **[2]TS6**

E(B3LYP)$_{sol}$ = -1273.67486717 E(B3LYP) = -1272.87319113

6	-1.660445	-0.576686	0.000015
9	-1.398496	-1.305175	-1.086176
9	-1.398605	-1.305198	1.086206
6	-0.781646	0.694752	0.000074
9	-1.018499	1.421958	-1.088167
9	-1.018452	1.421809	1.088424
17	-3.398681	-0.126592	-0.000069
53	1.503834	0.107837	-0.000057
6	3.963469	-0.688667	0.000142
1	4.026555	-1.149325	0.989200
1	3.959679	-1.364208	-0.858905
1	4.466346	0.273299	-0.130099

Frequencies -- -198.0746

Red. masses -- 10.6528

Frc consts -- 0.2462

IR Inten -- 2.1653

Structure S21. **Rf1**

E(B3LYP)$_{sol}$ = -935.949206149 E(B3LYP) = -935.209607462

6	0.348045	0.458865	-0.000841
9	0.683733	1.153752	-1.091129
9	0.683367	1.157724	1.087019
6	-1.143842	0.213438	-0.000665
9	-1.617080	-0.345490	-1.094213
9	-1.617676	-0.340357	1.095233
17	1.269628	-1.097910	0.002167

Structure S22. **[2]INT10**

E(B3LYP)$_{sol}$ = -1245.78942782 E(B3LYP) = -1244.71713609

6	-4.205614	-1.013096	-0.460547
6	-2.993494	-1.346859	0.127202
6	-2.028261	-0.347357	0.460471
6	-2.360801	1.007132	0.153827
6	-3.577961	1.328905	-0.434063
6	-4.511239	0.327868	-0.744525
1	-4.925235	-1.799355	-0.703294
1	-2.763388	-2.393250	0.346053
1	-1.641291	1.800490	0.358424
1	-3.805847	2.373521	-0.661859
1	-5.465643	0.589330	-1.207681
6	-0.809976	-0.730052	1.074371
1	-0.632487	-1.798401	1.221545
6	0.268098	0.205804	1.520255
1	-0.142441	1.155504	1.900955
1	0.860617	-0.239114	2.333153

6	2.146166	-0.521325	-0.105299
9	2.747344	-1.137897	0.927224
9	1.361209	-1.418193	-0.718169
6	1.274981	0.639110	0.449296
9	2.115157	1.557352	0.990125
9	0.643630	1.222116	-0.598225
17	3.382251	0.058829	-1.249519

Structure S23. **^{2}TS7**

E(B3LYP)$_{sol}$ = -1273.65953850 E(B3LYP) = -1272.85527817

6	0.863332	0.700944	0.000046
9	0.692788	1.431843	-1.086631
9	0.692787	1.431895	1.086688
6	-0.004784	-0.555349	0.000087
9	0.242094	-1.281788	-1.087586
9	0.241989	-1.281637	1.087881
17	2.839510	0.067335	0.000027
53	-2.179454	-0.010987	-0.000054
6	4.958003	-0.442421	-0.000164
1	4.915847	-1.533869	-0.039302
1	5.306623	0.057712	-0.907190
1	5.290713	-0.008083	0.945893

Frequencies -- -475.3052

Red. masses -- 16.9234

Frc consts -- 2.2526

IR Inten -- 67.0909

Structure S24. **Rf2**

E(B3LYP)$_{sol}$ = -773.524106215 E(B3LYP) = -772.931073102

6	-1.849933	-0.337180	-0.000087
9	-1.950091	-1.054514	-1.093324
9	-1.950623	-1.053949	1.093472
6	-0.806028	0.728851	-0.000119
9	-0.910620	1.485678	-1.091402
9	-0.910716	1.485863	1.091027
53	1.272344	-0.190901	0.000062

Structure S25. **^{2}TS8**

E(B3LYP)$_{sol}$ = -349.637030580 E(B3LYP) = -349.247290681

6	-2.422421	-0.755354	0.445847
6	-1.138166	-1.270400	0.265385
6	-0.079700	-0.457851	-0.194436
6	-0.362037	0.901831	-0.455696
6	-1.644335	1.416437	-0.273699
6	-2.683504	0.591845	0.176332
1	-3.224181	-1.408468	0.800447
1	-0.942891	-2.325005	0.480384
1	0.434637	1.565479	-0.798656
1	-1.837034	2.472474	-0.481043
1	-3.687646	0.998984	0.318757
6	1.250837	-1.041493	-0.368622
1	1.357593	-2.073341	-0.014664
6	2.353542	-0.421685	-0.865701
1	2.300268	0.558171	-1.343919
1	3.283732	-0.975382	-1.003880

6	3.389982	0.800061	1.038290
1	2.496194	1.369641	1.296894
1	3.646448	-0.062822	1.654246
1	4.187701	1.299918	0.485231

Frequencies -- -292.5131

Red. masses -- 9.7702

Frc consts -- 0.4925

IR Inten -- 4.5265

Structure S26. **[2]INT11**

E(B3LYP)$_{sol}$ = -349.693938710 E(B3LYP) = -349.310483392

6	-2.405426	-0.847803	0.246826
6	-1.105477	-1.312632	0.102204
6	-0.019730	-0.421959	-0.163463
6	-0.335140	0.967690	-0.270573
6	-1.640567	1.421985	-0.123113
6	-2.687375	0.523867	0.135795
1	-3.214443	-1.555091	0.448733
1	-0.896388	-2.382575	0.190752
1	0.461265	1.687555	-0.469326
1	-1.851800	2.491400	-0.209357
1	-3.711123	0.888037	0.250526
6	1.297988	-0.925447	-0.307961
1	1.436277	-2.003610	-0.172513
6	2.522197	-0.094142	-0.542590
1	2.300611	0.727751	-1.246315
1	3.293517	-0.713421	-1.030895
6	3.108844	0.503887	0.752962
1	2.375082	1.157698	1.250460
1	3.382958	-0.289569	1.466466
1	4.012159	1.099146	0.540938

Structure S27. **[5]INT12**

E(B3LYP)$_{sol}$ = -4459.00933989 E(B3LYP) = -4455.62967502

26	0.536871	-0.482545	-0.181296
8	-1.492225	-3.768923	-0.797147
16	-2.023803	-2.648305	-0.010876
8	-2.248679	-2.855379	1.426085
8	-1.358308	-1.313145	-0.309136
6	-3.702673	-2.330510	-0.781163
9	-4.443404	-3.433014	-0.719906
9	-4.350768	-1.342301	-0.164673
9	-3.542482	-1.989926	-2.067506
8	1.712657	2.348533	1.239488
8	0.500822	-0.384786	2.004732
6	1.996273	1.550134	2.365009
1	2.796627	0.820193	2.165662
1	2.320875	2.187714	3.215093
6	0.738794	0.808225	2.770505
1	0.803712	0.528242	3.832864
1	-0.131856	1.469380	2.636111
6	2.832337	2.975703	0.654871
1	2.457550	3.800484	0.031134
1	3.512811	3.400209	1.418178
1	3.397710	2.280827	0.017702
6	0.597741	-1.611916	2.737880
1	0.089941	-2.378727	2.142649
1	1.653924	-1.871510	2.901801

1	0.058334	-1.523273	3.694814
8	1.160791	-2.559499	-0.735468
8	0.404421	-0.500714	-2.371153
6	1.257903	-2.726184	-2.150663
1	2.287485	-2.479127	-2.454869
1	1.031174	-3.771064	-2.418784
6	0.242619	-1.843157	-2.835783
1	0.410577	-1.877893	-3.927641
1	-0.777901	-2.187660	-2.612277
6	1.588709	-3.708633	-0.003251
1	1.711205	-3.405922	1.040006
1	0.827268	-4.499412	-0.091950
1	2.570898	-4.042873	-0.371146
6	-0.512605	0.409826	-2.970540
1	-1.549579	0.125207	-2.720512
1	-0.301271	1.405482	-2.567864
1	-0.384556	0.409970	-4.066916
8	4.044767	-1.942011	-1.013478
16	3.676194	-1.042972	0.083464
8	2.550474	-0.068570	-0.258868
8	3.529670	-1.609440	1.434376
6	5.152444	0.107850	0.219008
9	5.234216	0.903981	-0.849220
9	6.260421	-0.619844	0.300471
9	5.067355	0.875666	1.312796
6	-0.846505	4.703255	-1.181599
6	-0.510913	4.749733	0.309880
1	-1.420315	4.636266	0.918688
1	0.190099	3.949092	0.582795
1	-0.062201	5.723489	0.558425
6	0.384648	4.960940	-2.051791
1	1.187537	4.252733	-1.802272
1	0.136913	4.848242	-3.117918
1	0.747520	5.985869	-1.884352
6	-2.003434	5.635985	-1.540412
1	-2.230757	5.574587	-2.615273
1	-2.906986	5.362339	-0.976086
1	-1.745484	6.677222	-1.292824
8	-1.185490	3.343524	-1.587629
8	-2.239448	2.854548	-0.751873
6	-1.846228	1.804099	0.009753
6	-2.949858	1.343121	0.883995
6	-4.242812	1.896593	0.804028
6	-2.686569	0.313614	1.801536
6	-5.251692	1.420094	1.638403
1	-4.454830	2.687454	0.084076
6	-3.702472	-0.162496	2.627498
1	-1.699931	-0.137795	1.828074
6	-4.983088	0.390577	2.549618
1	-6.256123	1.844766	1.571883
1	-3.494573	-0.994709	3.301979
1	-5.782372	0.010191	3.190915
8	-0.721210	1.346411	-0.043454

Structure S28. **^{5}INT12′**

E(B3LYP)$_{sol}$ = -3496.44800968 E(B3LYP) = -3494.14963993

26	0.000003	0.856470	0.000001
8	-1.247037	-0.617892	1.186654
16	-2.413934	-0.404981	0.261768
8	-3.728512	-0.190331	0.844385

8	-1.942861	0.640384	-0.745655
6	-2.505796	-1.976515	-0.749935
9	-2.824768	-2.990929	0.045433
9	-3.431850	-1.845885	-1.693178
9	-1.326975	-2.207303	-1.313564
8	3.728487	-0.190427	-0.844411
16	2.413912	-0.405041	-0.261775
8	1.942873	0.640350	0.745636
8	1.246999	-0.617945	-1.186643
6	2.505752	-1.976561	0.749953
9	3.431823	-1.845938	1.693181
9	2.824687	-2.990996	-0.045401
9	1.326935	-2.207311	1.313605
8	0.591300	2.523573	-1.229150
8	-0.591239	2.523599	1.229138
6	0.622931	3.703102	-0.430037
1	1.528501	3.697722	0.204087
1	0.636371	4.600595	-1.073716
6	-0.622719	3.703162	0.430072
1	-0.636047	4.600631	1.073788
1	-1.528288	3.697925	-0.204055
6	1.685481	2.377726	-2.139815
1	1.524769	1.434591	-2.673917
1	1.704317	3.224450	-2.845612
1	2.639810	2.314604	-1.591200
6	-1.685476	2.377821	2.139748
1	-2.639782	2.314781	1.591084
1	-1.524862	1.434666	2.673843
1	-1.704282	3.224535	2.845558

Structure S29. ^{7}INT9

E(B3LYP)$_{sol}$ = -4906.50624073 E(B3LYP) = -4902.95697997

26	-1.087429	-0.203255	-0.626905
8	1.283880	-2.876003	-0.618620
16	0.150865	-3.042764	0.291295
8	0.306732	-3.762577	1.546921
8	-0.629810	-1.727058	0.506245
6	-1.150633	-3.967049	-0.696435
9	-0.680646	-5.118266	-1.144886
9	-2.233076	-4.193447	0.054865
9	-1.530694	-3.209944	-1.742471
8	-2.896355	3.049182	-1.305551
16	-1.475591	2.860923	-0.993936
8	-1.267482	1.641233	-0.059256
8	-0.502234	2.902973	-2.081395
6	-1.007282	4.214479	0.211476
9	-1.815717	4.184974	1.269689
9	-1.112957	5.391161	-0.390294
9	0.247539	4.038899	0.622102
8	-2.502998	-0.129700	-2.214663
8	-3.051244	-0.591816	0.318917
6	-3.879794	-0.058158	-1.805174
1	-4.104409	0.983638	-1.524003
1	-4.527346	-0.376512	-2.638819
6	-4.032946	-0.989073	-0.628026
1	-5.045546	-0.889199	-0.199486
1	-3.869026	-2.042292	-0.921469
6	-2.237735	0.529946	-3.468220
1	-2.720116	-0.035973	-4.280786
1	-2.610781	1.563317	-3.429849

1	-1.152096	0.547492	-3.599457
6	-3.152870	-1.210474	1.608113
1	-3.000314	-2.296522	1.527231
1	-2.363948	-0.781781	2.231327
1	-4.140336	-0.982138	2.041202
7	0.350657	-0.267806	-1.767913
7	1.300723	-0.520043	-2.456046
7	2.206426	-0.749822	-3.115910
6	1.222497	0.785398	1.667481
6	0.321625	0.416263	2.695973
6	2.368777	-0.066023	1.220612
6	-0.685129	1.333884	3.128211
6	0.364571	-0.858961	3.336296
1	2.935031	-0.471195	2.075454
1	2.036858	-0.937278	0.631075
6	3.342252	0.716632	0.360187
6	-1.558390	1.008460	4.157922
1	-0.761929	2.303526	2.635225
6	-0.522100	-1.176098	4.357905
1	1.070252	-1.618086	2.997349
6	4.528205	-0.152264	-0.140356
9	3.854687	1.766086	1.047021
9	2.706776	1.220762	-0.733669
6	-1.482537	-0.245806	4.787153
1	-2.311870	1.734043	4.475613
1	-0.473429	-2.165410	4.819691
9	5.192309	-0.628135	0.926017
9	4.045351	-1.197049	-0.826296
17	5.645673	0.772920	-1.172435
1	-2.171678	-0.499121	5.596504
1	1.081234	1.749455	1.177287

Structure S30. DME

E(B3LYP)$_{sol}$ = -309.000989268 E(B3LYP) = -308.636336571

8	1.406436	-0.320934	-0.372607
8	-1.406381	-0.321080	0.372459
6	0.720588	0.745574	0.230608
1	0.740496	0.661830	1.337786
1	1.186621	1.719819	-0.035807
6	-0.720607	0.745576	-0.230564
1	-1.186697	1.719736	0.036048
1	-0.740518	0.662038	-1.337756
6	2.730612	-0.460053	0.062719
1	3.175141	-1.312291	-0.472771
1	3.340203	0.444129	-0.149999
1	2.794285	-0.658882	1.153047
6	-2.730630	-0.460012	-0.062665
1	-2.794503	-0.658607	-1.153024
1	-3.175121	-1.312335	0.472723
1	-3.340122	0.444166	0.150352

Optimized with SMD method

8	-1.408207	-0.337680	0.359008
8	1.408206	-0.337682	-0.359007
6	-0.720511	0.740571	-0.228523
1	-0.748882	0.677546	-1.337029
1	-1.184673	1.709607	0.055102
6	0.720511	0.740572	0.228523
1	1.184674	1.709606	-0.055104

1	0.748882	0.677548	1.337029
6	-2.743722	-0.445108	-0.058355
1	-3.199378	-1.296862	0.470808
1	-3.333955	0.466006	0.176527
1	-2.830002	-0.627631	-1.150074
6	2.743722	-0.445108	0.058355
1	2.830004	-0.627629	1.150074
1	3.199378	-1.296862	-0.470808
1	3.333954	0.466006	-0.176530

Structure S31. TBPB

E(B3LYP)$_{sol}$ = -653.496220775 E(B3LYP) = -652.754789754

6	2.850249	0.204810	0.184264
6	2.431379	-0.563307	1.439521
1	1.490252	-0.165463	1.847639
1	2.283281	-1.625674	1.202575
1	3.204184	-0.464686	2.217290
6	4.164746	-0.334418	-0.384325
1	4.082519	-1.412580	-0.585358
1	4.418649	0.178940	-1.323715
1	4.977236	-0.168219	0.338822
6	2.918493	1.713034	0.433278
1	3.202472	2.241307	-0.489404
1	1.941508	2.093193	0.765145
1	3.661658	1.939781	1.213379
8	1.941833	-0.070449	-0.913450
8	0.618008	0.355518	-0.566980
6	-0.229392	-0.704889	-0.390018
6	-1.625566	-0.212066	-0.174310
6	-1.987623	1.140487	-0.291993
6	-2.598066	-1.168977	0.157677
6	-3.311741	1.526463	-0.074598
1	-1.233256	1.881693	-0.557948
6	-3.919123	-0.777725	0.374749
1	-2.294381	-2.214473	0.238406
6	-4.277245	0.570433	0.259367
1	-3.592702	2.578326	-0.168538
1	-4.673191	-1.525065	0.633579
1	-5.312829	0.876903	0.428433
8	0.108651	-1.857200	-0.389817

Optimized with SMD method

6	2.843602	0.209909	0.204758
6	2.424293	-0.586842	1.440507
1	1.457665	-0.233077	1.830530
1	2.330810	-1.654345	1.195849
1	3.172954	-0.464955	2.238470
6	4.178932	-0.284515	-0.353575
1	4.132557	-1.360554	-0.580228
1	4.441959	0.257806	-1.274724
1	4.973422	-0.116529	0.389093
6	2.869124	1.714321	0.476711
1	3.130463	2.268314	-0.438370
1	1.887925	2.065419	0.829011
1	3.615611	1.946726	1.252090
8	1.952840	-0.076591	-0.912488
8	0.620124	0.340877	-0.584865
6	-0.231770	-0.715032	-0.437032
6	-1.621719	-0.219358	-0.194542

6	-1.989769	1.128882	-0.351364
6	-2.583065	-1.163506	0.204101
6	-3.307929	1.522177	-0.109706
1	-1.248847	1.864478	-0.667444
6	-3.897249	-0.763807	0.449203
1	-2.284262	-2.207568	0.317607
6	-4.261190	0.579269	0.292365
1	-3.592742	2.569802	-0.236396
1	-4.641021	-1.500843	0.762564
1	-5.291752	0.891251	0.482216
8	0.098998	-1.871151	-0.481251

Structure S32. acetone
E(B3LYP)$_{sol}$ = -193.248617123 E(B3LYP) = -193.017718953

6	0.000000	0.188968	-0.000002
6	-1.289581	-0.614501	0.000001
1	-2.151546	0.065589	0.000109
1	-1.332005	-1.270706	-0.885638
1	-1.331911	-1.270878	0.885519
6	1.289577	-0.614507	-0.000002
1	2.151548	0.065579	-0.000122
1	1.332003	-1.270691	0.885653
1	1.331903	-1.270895	-0.885509
8	0.000004	1.399030	0.000000

Structure S33. $TMSN_3$
E(B3LYP)$_{sol}$ = -573.662401640 E(B3LYP) = -573.220852941

14	0.673723	-0.000009	0.017349
6	0.782913	1.551315	1.076717
1	1.750353	1.597422	1.604725
1	0.687582	2.458818	0.458995
1	-0.015368	1.570893	1.837106
6	0.780126	-1.545995	1.084832
1	-0.019471	-1.561081	1.843944
1	0.685210	-2.456645	0.471692
1	1.746614	-1.590033	1.614757
6	1.948313	-0.004626	-1.353493
1	1.834833	-0.896339	-1.990512
1	1.839254	0.886248	-1.992470
1	2.969593	-0.006682	-0.938095
7	-0.895894	-0.000860	-0.827248
7	-2.012274	-0.000266	-0.344282
7	-3.088808	0.000178	0.028479

Structure S34. TMSOCOPh
E(B3LYP)$_{sol}$ = -829.796890969 E(B3LYP) = -829.098643161

14	2.450091	0.182937	0.000033
6	2.923420	-0.738886	1.560688
1	4.017262	-0.860882	1.630592
1	2.583560	-0.195097	2.457185
1	2.456604	-1.735347	1.560576
6	2.923747	-0.738916	-1.560505
1	2.456792	-1.735312	-1.560641
1	2.584364	-0.195025	-2.457119
1	4.017601	-0.861089	-1.629952
6	3.090802	1.944517	-0.000055

1	2.743291	2.490818	-0.891834
1	2.743988	2.490803	0.892000
1	4.193447	1.959158	-0.000496
8	0.728754	0.411426	-0.000081
6	-0.139686	-0.609024	-0.000038
6	-1.572299	-0.181597	-0.000033
8	0.209962	-1.772746	-0.000017
6	-1.942936	1.172371	0.000017
6	-2.563731	-1.175939	-0.000063
6	-3.293908	1.525773	0.000041
1	-1.164490	1.936410	0.000036
6	-3.912316	-0.819707	-0.000051
1	-2.251198	-2.221984	-0.000093
6	-4.278675	0.531669	0.000005
1	-3.581054	2.580305	0.000090
1	-4.681966	-1.595606	-0.000083
1	-5.335711	0.810730	0.000023

Structure S35. ClC_2F_4I
$E(B3LYP)_{sol}$ = -1233.81242196 E(B3LYP) = -1233.05975268

6	1.361457	-0.516713	0.000103
9	1.182090	-1.269938	1.085727
9	1.181687	-1.270233	-1.085244
6	0.331895	0.654064	0.000105
9	0.519507	1.403850	1.086811
9	0.519641	1.403935	-1.086515
17	3.023390	0.138156	-0.000303
53	-1.739322	-0.105307	-0.000058

Structure S36. CH_3I
$E(B3LYP)_{sol}$ = -337.738234904 E(B3LYP) = -337.675603497

6	-1.841716	0.000003	-0.000066
1	-2.176791	-0.888710	-0.546994
1	-2.175209	-0.029724	1.043532
1	-2.176797	0.918400	-0.495537
53	0.331681	0.000000	-0.000011

Structure S37. CH_3Cl
$E(B3LYP)_{sol}$ = -500.162125540 E(B3LYP) = -499.947744721

6	1.134023	0.000002	-0.000123
1	1.492213	-0.896909	-0.522783
1	1.492234	0.901662	-0.514535
1	1.491685	-0.004770	1.038194
17	-0.663545	0.000000	-0.000008

Structure S38. styrene
$E(B3LYP)_{sol}$ = -309.779660157 E(B3LYP) = -309.437873888

6	-1.785369	-1.046338	-0.000003
6	-0.409044	-1.284858	0.000001
6	0.516683	-0.223622	0.000005
6	0.011056	1.092485	0.000004
6	-1.361985	1.332552	0.000002
6	-2.268478	0.264630	-0.000003

1	-2.482771	-1.888084	-0.000007
1	-0.038628	-2.314077	-0.000001
1	0.700291	1.939709	0.000006
1	-1.730948	2.361610	0.000001
1	-3.344486	0.456250	-0.000008
6	1.958339	-0.531424	0.000003
1	2.194875	-1.602250	0.000012
6	2.980733	0.336900	-0.000007
1	2.835009	1.420716	-0.000016
1	4.015051	-0.015825	-0.000004

Structure S39. **[5]INT1-1**

E(B3LYP)$_{sol}$ = -2413.70775247 E(B3LYP) = -2412.24064756

26	0.002426	0.137797	-0.062631
8	-0.514148	1.929092	-1.216547
8	0.548050	1.794286	1.288651
6	0.052727	3.085859	-0.618117
1	1.134436	3.134773	-0.842397
1	-0.439446	3.999339	-1.000117
6	-0.158590	2.955309	0.875992
1	0.228402	3.850428	1.397221
1	-1.234700	2.835585	1.097106
6	-0.287509	1.776406	-2.613626
1	-0.828673	0.868231	-2.909573
1	-0.682799	2.646211	-3.167153
1	0.788279	1.644480	-2.815439
6	0.422287	1.454292	2.667673
1	-0.630818	1.241513	2.912826
1	1.023147	0.547118	2.808013
1	0.810364	2.271989	3.300311
8	-1.949164	0.225726	1.024101
6	-2.411974	-0.473184	0.079347
6	-3.862920	-0.823307	0.032047
8	-1.637927	-0.876803	-0.852284
6	-4.724292	-0.376054	1.045389
6	-4.369045	-1.597854	-1.022845
6	-6.081504	-0.699486	1.002781
1	-4.306945	0.222551	1.857143
6	-5.726802	-1.920347	-1.063649
1	-3.680026	-1.937507	-1.798196
6	-6.583611	-1.471487	-0.051707
1	-6.751691	-0.351341	1.793056
1	-6.120235	-2.524625	-1.885146
1	-7.646632	-1.725316	-0.084219
6	6.051704	-0.662927	-1.054542
6	4.686191	-0.373435	-1.078285
6	3.850534	-0.841010	-0.052701
6	4.391102	-1.602108	0.994997
6	5.756695	-1.890829	1.016902
6	6.587860	-1.421315	-0.007127
1	6.701762	-0.298618	-1.854304
1	4.243402	0.213810	-1.884705
1	3.721290	-1.958337	1.779800
1	6.176500	-2.484716	1.832922
1	7.657260	-1.648404	0.010471
6	2.389784	-0.531379	-0.074773
8	1.638389	-0.933082	0.862269
8	1.899578	0.154189	-1.029411

Structure S40. **[5]INT1-2**

E(B3LYP)$_{sol}$ = -2722.72812291 E(B3LYP) = -2720.91193634

26	0.244711	-0.700004	0.352256
8	0.585644	-2.238472	-1.153132
8	0.299733	-2.537037	1.541035
6	0.287689	-3.526376	-0.622351
1	-0.806211	-3.653332	-0.579920
1	0.742515	-4.313929	-1.251659
6	0.894275	-3.576476	0.763310
1	0.694424	-4.558168	1.230233
1	1.984553	-3.406170	0.709270
6	0.151594	-2.000860	-2.488881
1	0.416463	-0.958454	-2.709734
1	0.681419	-2.674820	-3.185775
1	-0.936922	-2.144935	-2.569376
6	0.835146	-2.401891	2.849933
1	1.916241	-2.185857	2.802016
1	0.316669	-1.553742	3.314825
1	0.659386	-3.319558	3.438559
8	0.238379	0.766922	2.120120
8	-1.857390	3.465872	0.877987
6	-0.827684	1.718853	2.072008
1	-1.752583	1.135344	1.997260
1	-0.845670	2.312489	3.002909
6	-0.721645	2.632525	0.856472
1	0.210754	3.233638	0.883780
1	-0.687609	2.012115	-0.057146
6	1.457457	1.263419	2.661930
1	2.168755	0.430994	2.669263
1	1.879773	2.069889	2.039019
1	1.293725	1.642145	3.686370
6	-1.991490	4.267823	-0.268413
1	-2.109937	3.656478	-1.185338
1	-2.890864	4.888417	-0.140690
1	-1.117391	4.935773	-0.409777
8	2.424737	-0.735519	0.627247
6	2.539324	0.153499	-0.274547
6	3.898802	0.628594	-0.679583
8	1.510034	0.645561	-0.821555
6	5.044873	0.083350	-0.081303
6	4.031637	1.626882	-1.656767
6	6.312305	0.533016	-0.456544
1	4.918143	-0.693298	0.675126
6	5.299522	2.075794	-2.030875
1	3.126154	2.035238	-2.109487
6	6.440626	1.529639	-1.431203
1	7.204196	0.106653	0.010070
1	5.401222	2.853422	-2.792345
1	7.433290	1.881516	-1.724861
6	-5.321735	1.694593	-0.353894
6	-4.086868	1.057319	-0.203733
6	-3.940687	-0.291519	-0.562732
6	-5.045580	-0.990645	-1.073382
6	-6.278566	-0.353524	-1.222646
6	-6.419198	0.992369	-0.863140
1	-5.425507	2.745193	-0.069166
1	-3.236697	1.609301	0.195120
1	-4.908338	-2.038454	-1.347446
1	-7.134335	-0.905772	-1.620464
1	-7.384688	1.492523	-0.979629
6	-2.620806	-1.015214	-0.423905

8	-2.523906	-2.188760	-0.785007
8	-1.653153	-0.308761	0.076030

Structure S41. [5]**INT1-3**

E(B3LYP)$_{sol}$ = -3264.11496416 E(B3LYP) = -3261.88462631

26	-0.032331	-0.272553	0.390157
8	-0.605199	0.857081	2.239311
8	-0.230466	1.862290	-0.286623
6	-0.802714	2.242534	1.959931
1	-1.844188	2.404680	1.632702
1	-0.604898	2.844774	2.865702
6	0.134599	2.641064	0.841725
1	0.009771	3.718130	0.625155
1	1.185106	2.440895	1.117734
6	-1.634246	0.288873	3.046685
1	-1.440400	-0.788386	3.105843
1	-1.619209	0.734422	4.056727
1	-2.619725	0.434103	2.577002
6	0.401405	2.201167	-1.514107
1	1.460518	1.895149	-1.499051
1	-0.152727	1.663691	-2.292994
1	0.322251	3.286476	-1.701504
8	0.043265	-1.756806	-1.253208
8	0.062147	-2.186105	1.429372
6	-0.512623	-2.974804	-0.753954
1	-1.599797	-2.850377	-0.617047
1	-0.333144	-3.795080	-1.471008
6	0.202610	-3.299442	0.539398
1	-0.237291	-4.203224	0.999304
1	1.274647	-3.449090	0.339139
6	-0.106875	-1.547285	-2.652770
1	0.359098	-0.580919	-2.879746
1	0.415202	-2.343985	-3.211079
1	-1.168459	-1.509891	-2.940277
6	0.894283	-2.271133	2.586618
1	1.942318	-2.420993	2.287056
1	0.805604	-1.312659	3.114164
1	0.552322	-3.091885	3.241438
8	-3.599115	-1.459063	-1.434623
16	-2.946625	-0.211075	-1.027982
8	-2.068974	-0.388536	0.202648
8	-2.331983	0.617260	-2.074738
6	-4.289650	0.862721	-0.289818
9	-4.826282	0.272584	0.781954
9	-5.247024	1.092458	-1.181575
9	-3.774662	2.039221	0.098819
8	1.892152	0.143407	0.379033
6	2.937319	-0.602988	0.189824
6	4.206805	0.147924	-0.149106
8	2.958781	-1.830797	0.268393
6	4.253899	1.549954	-0.167310
6	5.367911	-0.579850	-0.451152
6	5.440961	2.214634	-0.485997
1	3.350002	2.108834	0.076942
6	6.553761	0.082368	-0.773784
1	5.311314	-1.669779	-0.425854
6	6.592712	1.481660	-0.792103
1	5.469728	3.307580	-0.495547
1	7.452994	-0.492337	-1.011259
1	7.521468	2.000864	-1.043693

Structure S42. **[5]INT1-4**

E(B3LYP)sol = -2302.15482650 E(B3LYP) = -2300.75972003

26	0.894627	0.000114	-0.000401
8	2.359300	0.941197	-1.242762
8	1.185464	1.970447	0.919462
6	2.641520	2.306234	-0.918531
1	3.549994	2.351834	-0.290509
1	2.816431	2.888175	-1.839616
6	1.442766	2.855305	-0.175743
1	1.662685	3.872275	0.192106
1	0.553880	2.883985	-0.829990
6	3.373954	0.263364	-1.984660
1	3.025797	-0.767856	-2.119330
1	3.518522	0.743701	-2.965964
1	4.328493	0.267062	-1.429769
6	0.126409	2.398530	1.787701
1	-0.025965	1.597763	2.522011
1	0.410152	3.332870	2.298135
1	-0.800415	2.545959	1.210866
8	2.359254	-0.940857	1.241996
8	1.185355	-1.970648	-0.919843
6	2.640501	-2.306390	0.919010
1	3.549400	-2.353230	0.291703
1	2.814246	-2.887784	1.840664
6	1.441752	-2.855105	0.175946
1	1.661266	-3.872364	-0.191338
1	0.552572	-2.882946	0.829814
6	3.372773	-0.264034	1.986320
1	3.025680	0.767754	2.119412
1	3.513871	-0.743948	2.968344
1	4.328883	-0.269456	1.434163
6	0.126484	-2.398777	-1.788269
1	-0.800806	-2.544924	-1.211855
1	-0.024782	-1.598690	-2.523550
1	0.409788	-3.333876	-2.297560
6	-5.149356	0.801362	-0.913471
6	-3.754725	0.802794	-0.915813
6	-3.052754	0.000055	0.000017
6	-3.754819	-0.802578	0.915869
6	-5.149449	-0.800920	0.913589
6	-5.845787	0.000281	0.000075
1	-5.698038	1.423677	-1.624164
1	-3.191615	1.417715	-1.620101
1	-3.191777	-1.417594	1.620129
1	-5.698202	-1.423149	1.624302
1	-6.938687	0.000370	0.000097
6	-1.573834	-0.000077	0.000002
8	-0.914987	-0.730474	0.814680
8	-0.914923	0.730265	-0.814760

Structure S43. **[5]INT2-1**

E(B3LYP)sol = -2758.19270845 E(B3LYP) = -2756.34571569

26	-0.352900	-0.651293	-0.438511
8	-2.260435	-0.469682	0.734472
6	-2.828833	-0.866182	-0.321711
6	-4.311116	-1.025939	-0.378780
8	-2.127491	-1.131677	-1.355254

6	-5.089804	-0.746857	0.754706
6	-4.929240	-1.454866	-1.563307
6	-6.476738	-0.894869	0.703192
1	-4.586845	-0.417060	1.665567
6	-6.316682	-1.601644	-1.612483
1	-4.303316	-1.667752	-2.431704
6	-7.090887	-1.321900	-0.480284
1	-7.082927	-0.678219	1.586605
1	-6.797903	-1.936016	-2.535153
1	-8.177375	-1.437953	-0.519936
6	5.380605	-2.673309	-1.503920
6	4.137500	-2.039314	-1.461350
6	3.102429	-2.574807	-0.680009
6	3.319009	-3.750214	0.055154
6	4.562520	-4.383235	0.010420
6	5.594090	-3.845068	-0.768206
1	6.186605	-2.255581	-2.112889
1	3.943498	-1.125990	-2.026733
1	2.497131	-4.150802	0.651570
1	4.729987	-5.299888	0.581939
1	6.567594	-4.341723	-0.803037
6	1.774502	-1.893878	-0.629300
8	0.852845	-2.348582	0.112946
8	1.574066	-0.838941	-1.309426
6	0.511487	0.354867	2.755702
6	-0.654025	1.310186	2.993840
1	-0.349320	2.355796	2.836437
1	-1.484465	1.059888	2.319157
1	-0.999912	1.212149	4.034005
6	0.105100	-1.106613	2.949671
1	-0.820100	-1.327746	2.401937
1	0.892337	-1.781629	2.587706
1	-0.062772	-1.280924	4.023213
6	1.757341	0.709836	3.562639
1	2.580550	0.022374	3.318038
1	2.083950	1.739667	3.358777
1	1.536008	0.626460	4.637334
8	0.871089	0.375231	1.321528
8	1.279528	1.707921	0.960795
6	0.611181	2.156681	-0.121798
6	0.987890	3.544107	-0.474698
6	1.905689	4.291319	0.284716
6	0.387735	4.108923	-1.613034
6	2.215276	5.596740	-0.096465
1	2.373651	3.848397	1.164715
6	0.702449	5.414551	-1.986562
1	-0.318810	3.509065	-2.189339
6	1.615035	6.158433	-1.229443
1	2.928609	6.179102	0.491199
1	0.236655	5.854156	-2.871470
1	1.861193	7.181616	-1.524501
8	-0.201315	1.486297	-0.731847

Structure S44. ^{5}**TS1-1**

E(B3LYP)$_{sol}$ = -2758.18815204 E(B3LYP) = -2756.34075173

26	0.021071	-0.476160	-0.148823
8	-1.855648	-0.995741	0.745781
6	-2.177045	-1.610056	-0.326493
6	-3.499044	-2.290421	-0.434095
8	-1.362615	-1.629492	-1.291953

6	-4.396276	-2.267065	0.644450
6	-3.846672	-2.957373	-1.618931
6	-5.632840	-2.906055	0.537908
1	-4.104720	-1.744487	1.557177
6	-5.083865	-3.595060	-1.723089
1	-3.131497	-2.962778	-2.443543
6	-5.977299	-3.569812	-0.645517
1	-6.331464	-2.888532	1.378386
1	-5.354371	-4.114709	-2.645827
1	-6.945941	-4.070211	-0.728104
6	5.922752	-1.607558	-1.570665
6	4.613263	-1.146671	-1.423867
6	3.714356	-1.840275	-0.599464
6	4.132478	-2.997984	0.074287
6	5.442344	-3.456904	-0.074564
6	6.337905	-2.762041	-0.896313
1	6.623218	-1.067834	-2.213194
1	4.263037	-0.250902	-1.940019
1	3.413302	-3.522487	0.706184
1	5.767972	-4.359506	0.448937
1	7.363499	-3.122755	-1.012847
6	2.316130	-1.347841	-0.438889
8	1.505954	-1.956070	0.325336
8	1.928672	-0.306563	-1.056124
6	0.232200	0.710972	2.841223
6	-1.184174	1.206855	3.118632
1	-1.269233	2.284193	2.914704
1	-1.895889	0.657628	2.486565
1	-1.435926	1.035950	4.176323
6	0.368186	-0.795087	3.115489
1	-0.442671	-1.351863	2.628849
1	1.326585	-1.179472	2.741240
1	0.312839	-0.948682	4.204418
6	1.309696	1.506611	3.573737
1	2.309065	1.139476	3.297230
1	1.247086	2.575929	3.329366
1	1.179192	1.391035	4.660287
8	0.497389	0.779195	1.404892
8	0.418312	2.325753	0.991540
6	-0.161532	2.428922	-0.191808
6	-0.350376	3.838554	-0.630085
6	0.007871	4.928540	0.181383
6	-0.908575	4.056284	-1.900641
6	-0.196104	6.229232	-0.279514
1	0.444128	4.752848	1.165772
6	-1.108902	5.359670	-2.354114
1	-1.176216	3.194005	-2.513695
6	-0.753682	6.445272	-1.545133
1	0.081138	7.078411	0.349378
1	-1.542694	5.530605	-3.342085
1	-0.911357	7.465884	-1.903091
8	-0.514775	1.469691	-0.874045

Frequencies -- -585.8323

Red. masses -- 13.9963

Frc consts -- 2.8302

IR Inten -- 1692.1061

Structure S45. **[7]INT3-1**

E(B3LYP)$_{sol}$ = -2758.21917226 E(B3LYP) = -2756.37214982

26	0.068175	0.046115	-0.010026

8	0.434125	-1.819684	0.907833
6	0.805031	-2.276892	-0.221039
6	1.295644	-3.666529	-0.366582
8	0.741169	-1.492477	-1.221991
6	1.362166	-4.507950	0.755454
6	1.693345	-4.138906	-1.627656
6	1.824602	-5.816615	0.614564
1	1.048876	-4.118725	1.725856
6	2.155038	-5.448412	-1.763385
1	1.633231	-3.465875	-2.484897
6	2.220487	-6.286459	-0.643658
1	1.877813	-6.473983	1.485817
1	2.465453	-5.819070	-2.743325
1	2.582594	-7.312261	-0.752143
6	3.861836	4.380734	-2.095062
6	2.837756	3.478741	-1.804309
6	3.008558	2.537388	-0.776849
6	4.205861	2.501137	-0.044283
6	5.226951	3.404814	-0.339095
6	5.054998	4.343978	-1.363408
1	3.731989	5.115185	-2.893680
1	1.898482	3.487086	-2.360274
1	4.315192	1.758539	0.748325
1	6.160114	3.379216	0.228958
1	5.856136	5.051558	-1.592994
6	1.923986	1.578413	-0.461700
8	2.050099	0.716189	0.459330
8	0.823595	1.598716	-1.111731
6	-0.488313	0.731980	3.051924
6	-1.374722	-0.494961	3.307997
1	-2.412466	-0.263018	3.032587
1	-1.026179	-1.347195	2.709676
1	-1.339414	-0.767635	4.374122
6	0.983842	0.418367	3.432603
1	1.336950	-0.463133	2.881707
1	1.633993	1.268109	3.184322
1	1.028118	0.224439	4.515614
6	-0.999483	1.972060	3.790867
1	-0.344536	2.832746	3.588361
1	-2.011401	2.213994	3.437798
1	-1.026010	1.792192	4.876882
8	-0.432851	1.036391	1.679156
8	-2.643059	1.295253	1.141080
6	-2.748144	0.711727	0.049475
6	-4.070666	0.718482	-0.661411
6	-5.167674	1.379282	-0.086291
6	-4.213506	0.068229	-1.897112
6	-6.398667	1.387829	-0.742765
1	-5.036207	1.880838	0.874063
6	-5.447294	0.079368	-2.550408
1	-3.349504	-0.437982	-2.330103
6	-6.539584	0.737924	-1.974817
1	-7.252044	1.902757	-0.294473
1	-5.557856	-0.426590	-3.512784
1	-7.504646	0.745645	-2.488393
8	-1.792267	0.075836	-0.534761

Structure S46. ^{7}**TS2-1**

E(B3LYP)$_{sol}$ = -2758.20734240 E(B3LYP) = -2756.35400849

26	0.077229	-0.040231	0.165080

8	-0.775396	1.788837	0.879410
6	-1.074060	2.085704	-0.322812
6	-1.813259	3.329948	-0.645842
8	-0.722157	1.277931	-1.237806
6	-2.181774	4.215815	0.378743
6	-2.142979	3.619591	-1.979342
6	-2.877218	5.385285	0.069792
1	-1.914534	3.971541	1.408380
6	-2.839000	4.789900	-2.283917
1	-1.845689	2.915817	-2.758893
6	-3.205793	5.672198	-1.260640
1	-3.164725	6.076678	0.865733
1	-3.097231	5.017122	-3.321118
1	-3.750691	6.588902	-1.501284
6	-2.779734	-5.030233	-1.955783
6	-1.951898	-3.966607	-1.593057
6	-2.440961	-2.957422	-0.749107
6	-3.760069	-3.016410	-0.272777
6	-4.584685	-4.080806	-0.638318
6	-4.094688	-5.087757	-1.479074
1	-2.400872	-5.817341	-2.612436
1	-0.923556	-3.899113	-1.952576
1	-4.117825	-2.216984	0.378941
1	-5.612484	-4.127813	-0.269761
1	-4.741769	-5.921329	-1.764956
6	-1.567834	-1.822193	-0.354779
8	-1.970900	-0.912241	0.415253
8	-0.364195	-1.759254	-0.801795
6	0.452108	-0.402275	3.194427
6	0.951068	1.003985	3.426105
1	2.035065	0.951843	3.224036

1	0.507448	1.704146	2.709199
1	0.778932	1.338065	4.458065
6	-1.618797	-0.079177	3.527084
1	-1.781465	0.661728	2.741621
1	-1.998046	-1.081386	3.320637
1	-1.605502	0.258086	4.566832
6	0.760463	-1.431802	4.259431
1	0.220690	-2.367501	4.062168
1	1.842961	-1.634813	4.179465
1	0.546595	-1.066853	5.273147
8	0.328023	-0.849954	2.013748
8	3.249631	-0.195528	1.647850
6	3.073106	-0.041281	0.449380
6	4.200616	-0.140017	-0.538464
6	5.495692	-0.403079	-0.065585
6	3.981676	0.020906	-1.915358
6	6.561524	-0.503660	-0.960273
1	5.640295	-0.525348	1.009624
6	5.049950	-0.080891	-2.809307
1	2.969472	0.224582	-2.268334
6	6.339555	-0.342702	-2.333520
1	7.569142	-0.708460	-0.589214
1	4.876986	0.044017	-3.881333
1	7.174778	-0.422041	-3.034672
8	1.903242	0.232752	-0.093836

Frequencies -- -331.6695

Red. masses -- 7.3535

Frc consts -- 0.4766

IR Inten -- 28.3766

Structure S47. [6]**INT4-1**

E(B3LYP)sol = -2525.08687778 E(B3LYP) = -2523.49449336

26	-0.000055	-0.000132	-0.001154
8	-1.374049	-1.192995	-0.982335
6	-2.176864	-1.060273	-0.000335
6	-3.505837	-1.707766	0.000096
8	-1.786010	-0.346061	0.981217
6	-3.908283	-2.478882	-1.102510
6	-4.360497	-1.549182	1.103151
6	-5.162826	-3.088721	-1.099250
1	-3.226078	-2.586503	-1.947799
6	-5.613911	-2.161342	1.100781
1	-4.024325	-0.945389	1.948090
6	-6.014390	-2.930062	0.000987
1	-5.480249	-3.689440	-1.954990
1	-6.282120	-2.040881	1.956875
1	-6.997164	-3.408922	0.001334
6	0.934477	5.942321	1.100891
6	0.838029	4.550747	1.103252
6	0.273906	3.889808	-0.000034
6	-0.192236	4.623825	-1.102871
6	-0.093194	6.015219	-1.099613
6	0.469431	6.673441	0.000867
1	1.372476	6.460833	1.957170
1	1.192501	3.957757	1.948362
1	-0.626135	4.086772	-1.948333
1	-0.454377	6.590422	-1.955533
1	0.546031	7.763984	0.001223
6	0.170265	2.415125	-0.000420
8	-0.345735	1.786122	-0.982544
8	0.593071	1.719626	0.981312
8	1.719782	-0.593835	-0.982579
6	2.006646	-1.354959	-0.000309
6	3.231991	-2.181979	0.000119
6	4.100511	-2.145578	-1.102905
6	3.522554	-3.000693	1.103569
6	5.255976	-2.927040	-1.099676
1	3.852168	-1.501520	-1.948491
6	4.679479	-3.779992	1.101184
1	2.831938	-3.010987	1.948817
6	5.544933	-3.743105	0.000961
1	5.934524	-2.902079	-1.955749
1	4.909712	-4.418331	1.957588
1	6.451076	-4.354707	0.001289
8	1.193002	-1.373233	0.981497

Structure S48. [5]**INT2-2**

E(B3LYP)sol = -3067.21080486 E(B3LYP) = -3065.01585675

26	-0.515153	-0.170345	0.353570
8	-1.282070	-1.902883	1.670934
8	0.162142	-4.841856	0.086045
6	-0.536551	-3.113776	1.522693
1	0.511349	-2.849206	1.704575
1	-0.862040	-3.849944	2.278997
6	-0.662708	-3.699839	0.120812
1	-1.713814	-3.966432	-0.112731
1	-0.342632	-2.940997	-0.615693
6	-2.679120	-2.094058	1.861425
1	-3.122484	-1.109026	2.040714

1	-3.154621	-2.527517	0.966709	6	2.401774	-0.696215	0.011249
1	-2.859928	-2.750872	2.730323	8	2.745570	0.486133	0.037697
6	0.268094	-5.423050	-1.189431	8	1.187796	-1.121152	0.194755
1	0.726116	-4.727523	-1.920692	6	-0.473815	2.089929	2.898849
1	0.909147	-6.312767	-1.100586	6	-1.407803	3.151767	2.323860
1	-0.720114	-5.736535	-1.583517	1	-0.836356	3.988844	1.897578
8	-2.489526	0.635775	0.434151	1	-2.038029	2.704381	1.542174
6	-2.840517	-0.058672	-0.580864	1	-2.051588	3.548740	3.123554
6	-4.229202	0.070384	-1.116629	6	-1.250874	0.918747	3.498278
8	-2.024849	-0.861693	-1.104865	1	-2.026646	0.582782	2.800197
6	-5.144500	0.947057	-0.514596	1	-0.580884	0.075705	3.720691
6	-4.623189	-0.696444	-2.224005	1	-1.729436	1.248528	4.432310
6	-6.443350	1.054668	-1.015579	6	0.550570	2.655101	3.879433
1	-4.817801	1.535616	0.344521	1	1.221184	1.858623	4.234830
6	-5.921962	-0.587306	-2.723692	1	1.157264	3.439368	3.405976
1	-3.892438	-1.369101	-2.676981	1	0.031359	3.093639	4.745099
6	-6.832931	0.287797	-2.119949	8	0.251129	1.434754	1.797503
1	-7.155408	1.738425	-0.546198	8	1.037302	2.433555	1.116783
1	-6.227168	-1.184193	-3.587031	6	0.941712	2.301519	-0.224664
1	-7.849921	0.373120	-2.512206	6	1.871517	3.217650	-0.918638
6	4.086821	-4.094669	-0.484090	6	3.066284	3.643904	-0.317705
6	3.104712	-3.132220	-0.233613	6	1.543715	3.626573	-2.219986
6	3.433053	-1.768322	-0.251041	6	3.920982	4.494545	-1.018298
6	4.755320	-1.382581	-0.522255	1	3.328517	3.273130	0.673114
6	5.734626	-2.344296	-0.775770	6	2.400452	4.483327	-2.911122
6	5.401953	-3.704246	-0.757228	1	0.619685	3.262504	-2.673133
1	3.821111	-5.155125	-0.464153	6	3.587014	4.918843	-2.310024
1	2.083496	-3.443426	-0.019178	1	4.858354	4.819894	-0.560846
1	4.988757	-0.316202	-0.530074	1	2.146463	4.807928	-3.922976
1	6.761613	-2.035264	-0.988807	1	4.260606	5.585189	-2.855188
1	6.168662	-4.458595	-0.954738	8	0.118647	1.590940	-0.774750

Structure S49. ^{5}**TS1-2**

E(B3LYP)$_{sol}$ = -3067.20332460 E(B3LYP) = -3065.00817582

26	0.485992	0.085967	0.436518
8	1.260784	1.908424	1.622082
8	-0.139329	4.797924	-0.088624
6	0.525475	3.119459	1.418921
1	-0.524673	2.875203	1.613212
1	0.860210	3.883794	2.142027
6	0.660805	3.640502	-0.007217
1	1.716884	3.873805	-0.253373
1	0.323281	2.857053	-0.708639
6	2.654318	2.106106	1.836877
1	3.101122	1.123096	2.015853
1	3.140067	2.549225	0.952718
1	2.815411	2.758442	2.712661
6	-0.240817	5.323501	-1.388410
1	-0.719042	4.606279	-2.084957
1	-0.861107	6.230650	-1.336108
1	0.751505	5.596537	-1.801773
8	2.501897	-0.670277	0.521405
6	2.802424	0.003779	-0.519462
6	4.169758	-0.101337	-1.107373
8	1.943286	0.775278	-1.029419
6	5.127443	-0.946846	-0.526882
6	4.501385	0.654126	-2.242783
6	6.407666	-1.033936	-1.076973
1	4.847935	-1.528090	0.353626
6	5.781885	0.565416	-2.791092
1	3.738165	1.301186	-2.679153
6	6.735625	-0.277969	-2.208611
1	7.153096	-1.693306	-0.624995
1	6.039239	1.153115	-3.675992
1	7.738181	-0.347372	-2.639221
6	-4.057038	4.039187	-0.621528
6	-3.081429	3.083776	-0.323177
6	-3.419854	1.723482	-0.267009
6	-4.746096	1.334219	-0.513397
6	-5.718848	2.288585	-0.814689
6	-5.375920	3.645032	-0.869542
1	-3.782995	5.097024	-0.658639
1	-2.058009	3.399493	-0.128557
1	-4.987707	0.270814	-0.463736
1	-6.748812	1.976483	-1.007768
1	-6.137590	4.393698	-1.104502
6	-2.399559	0.655887	0.049176
8	-2.750252	-0.518360	0.139301
8	-1.176754	1.081568	0.206375
6	0.412420	-1.821376	3.047223
6	1.383388	-2.949521	2.700959
1	0.844416	-3.811999	2.285209
1	2.112847	-2.591159	1.960970
1	1.914998	-3.275845	3.608046
6	1.157980	-0.626409	3.658000
1	2.021214	-0.365261	3.035097
1	0.500798	0.250198	3.741570
1	1.511344	-0.908676	4.661558
6	-0.741580	-2.269710	3.942011
1	-1.429891	-1.430942	4.123356
1	-1.304819	-3.086195	3.471096

1	-0.350017	-2.624964	4.907482
8	-0.136091	-1.252611	1.829720
8	-0.943486	-2.499980	1.101663
6	-0.888877	-2.325054	-0.197606
6	-1.777187	-3.263061	-0.931228
6	-2.979526	-3.711461	-0.363664
6	-1.403914	-3.663618	-2.222512
6	-3.801981	-4.572050	-1.090839
1	-3.270093	-3.348970	0.622554
6	-2.227626	-4.532178	-2.939079
1	-0.474380	-3.282061	-2.649174
6	-3.424521	-4.987417	-2.373334
1	-4.746597	-4.913697	-0.660860
1	-1.940202	-4.850142	-3.944194
1	-4.071405	-5.662891	-2.939253
8	-0.131803	-1.536511	-0.769524

Frequencies -- -619.1538

Red. masses -- 14.0481

Frc consts -- 3.1730

IR Inten -- 1264.3067

Structure S50. **^{7}INT3-2**

E(B3LYP)$_{sol}$ = -3067.23523697 E(B3LYP) = -3065.03565673

26	-0.571335	-0.282199	0.297033
8	-0.768111	-2.394348	0.837101
8	2.306820	-4.291960	0.488358
6	0.436793	-3.071285	1.208177
1	1.115080	-2.289984	1.570930
1	0.223423	-3.766123	2.039463
6	1.069234	-3.804451	0.034645
1	0.419577	-4.636686	-0.310919
1	1.186661	-3.091498	-0.802825
6	-1.888019	-3.216004	0.517982
1	-2.787838	-2.609918	0.673462
1	-1.846723	-3.553748	-0.529588
1	-1.917652	-4.087054	1.193065
6	3.048758	-4.963039	-0.501198
1	3.292756	-4.297693	-1.351749
1	3.988610	-5.301959	-0.041399
1	2.506033	-5.847937	-0.891905
8	-2.761201	-0.173506	0.590350
6	-2.867406	-0.325750	-0.662426
6	-4.182897	-0.248586	-1.344558
8	-1.806937	-0.569471	-1.329752
6	-5.345385	-0.009256	-0.594598
6	-4.263832	-0.415408	-2.736250
6	-6.583334	0.058573	-1.234542
1	-5.255725	0.123292	0.485205
6	-5.503973	-0.345709	-3.372232
1	-3.345383	-0.593255	-3.298651
6	-6.662601	-0.109939	-2.622376
1	-7.489643	0.245806	-0.653369
1	-5.569787	-0.472761	-4.455588
1	-7.633053	-0.054758	-3.122756
6	5.147099	-1.913153	-0.620756
6	3.815652	-1.501789	-0.514007
6	3.307323	-0.530568	-1.389534
6	4.147705	0.028235	-2.365790
6	5.476136	-0.384473	-2.471535
6	5.978590	-1.357395	-1.598462

1	5.535220	-2.670735	0.065069
1	3.170031	-1.942058	0.244448
1	3.731814	0.787932	-3.030401
1	6.124728	0.053208	-3.234929
1	7.020146	-1.680109	-1.679820
6	1.882850	-0.049154	-1.313758
8	1.470301	0.841265	-2.033942
8	1.143147	-0.676440	-0.413304
6	-0.814119	0.133940	3.440238
6	-1.708619	1.373038	3.298317
1	-1.086573	2.272267	3.196521
1	-2.349504	1.278462	2.412200
1	-2.342656	1.484685	4.191580
6	-1.689487	-1.125950	3.653522
1	-2.408746	-1.223941	2.829905
1	-1.062074	-2.027956	3.697430
1	-2.235538	-1.026013	4.604306
6	0.204223	0.295916	4.573070
1	0.821766	-0.610525	4.664628
1	0.865596	1.143433	4.346099
1	-0.305153	0.478352	5.532304
8	-0.095370	-0.124951	2.253936
8	0.996793	1.819721	1.874539
6	0.488275	2.264979	0.818128
6	0.989632	3.567495	0.279754
6	1.656930	4.472539	1.119149
6	0.810127	3.860115	-1.081217
6	2.122411	5.682371	0.602853
1	1.800726	4.216109	2.170461
6	1.289648	5.066975	-1.592855
1	0.329272	3.117067	-1.718213
6	1.938294	5.979672	-0.752924
1	2.633396	6.394258	1.255960
1	1.161182	5.295218	-2.653875
1	2.308234	6.925583	-1.157732
8	-0.428385	1.674013	0.154830

Structure S51. ^{7}**TS2-2**

E(B3LYP)$_{sol}$ = -3067.22030501 E(B3LYP) = -3065.01387279

26	-0.567149	-0.182906	0.379659
8	-0.826493	-2.458444	0.335708
8	2.318289	-4.231008	-0.078060
6	0.333256	-3.223989	0.663670
1	0.954847	-2.570861	1.286088
1	0.035154	-4.104135	1.263523
6	1.127288	-3.661298	-0.559326
1	0.555491	-4.396038	-1.165412
1	1.320737	-2.776611	-1.192876
6	-1.833204	-3.144921	-0.398655
1	-2.784385	-2.634224	-0.208683
1	-1.622066	-3.132002	-1.479946
1	-1.913452	-4.188340	-0.048456
6	3.184661	-4.674046	-1.094248
1	3.494311	-3.845101	-1.759139
1	4.082599	-5.086663	-0.611453
1	2.718700	-5.467263	-1.714346
8	-2.803444	-0.278815	0.578423
6	-2.835432	-0.102944	-0.675417
6	-4.118500	0.073092	-1.402751
8	-1.733293	-0.101220	-1.315620

6	-5.333148	0.035857	-0.700157
6	-4.114901	0.274850	-2.792162
6	-6.538316	0.195530	-1.385025
1	-5.310374	-0.115507	0.380634
6	-5.322374	0.435219	-3.473180
1	-3.157501	0.307109	-3.315535
6	-6.532926	0.394733	-2.770977
1	-7.485064	0.168173	-0.839841
1	-5.322027	0.594910	-4.554217
1	-7.477602	0.521711	-3.306374
6	5.216415	-1.687160	-0.404298
6	3.870315	-1.320010	-0.326726
6	3.345188	-0.379023	-1.225150
6	4.181722	0.193457	-2.196360
6	5.526258	-0.171598	-2.270055
6	6.045981	-1.114029	-1.373740
1	5.617936	-2.425164	0.295099
1	3.217569	-1.775344	0.417203
1	3.749751	0.925652	-2.881294
1	6.173842	0.278806	-3.026890
1	7.099645	-1.400595	-1.431448
6	1.899298	0.039368	-1.186609
8	1.438098	0.826605	-1.992993
8	1.197952	-0.534142	-0.225405
6	-0.899715	-0.445936	3.432740
6	-1.925812	0.660324	3.501060
1	-1.330204	1.587133	3.563330
1	-2.528278	0.701110	2.587132
1	-2.562653	0.571163	4.391624
6	-2.197987	-2.083305	3.326301
1	-2.714580	-1.806162	2.405389
1	-1.484629	-2.906988	3.258628
1	-2.741222	-1.975586	4.268671
6	-0.132114	-0.747962	4.702065
1	0.462080	-1.665090	4.592438
1	0.562718	0.100192	4.832513
1	-0.782558	-0.810721	5.585007
8	-0.281575	-0.692412	2.348626
8	0.630206	2.231317	2.633456
6	0.394357	2.404693	1.445864
6	1.021267	3.520929	0.665195
6	1.786687	4.478867	1.349667
6	0.867439	3.611450	-0.726628
6	2.386491	5.525447	0.649618
1	1.897716	4.379339	2.431253
6	1.476891	4.656610	-1.424821
1	0.303874	2.840375	-1.251231
6	2.231382	5.614880	-0.739808
1	2.979507	6.272386	1.184001
1	1.367839	4.717237	-2.510585
1	2.705785	6.432448	-1.289829
8	-0.439795	1.648834	0.755033

Frequencies -- -359.2748

Red. masses -- 7.6894

Frc consts -- 0.5848

IR Inten -- 25.6571

Structure S52. **^{6}INT4-2**

E(B3LYP)$_{sol}$ = -2834.10578373 E(B3LYP) = -2832.16415825

26	0.279689	0.236704	-0.087374

8	0.697112	-0.791702	-1.937901
8	0.054001	-4.260909	-1.217696
6	0.131963	-2.095453	-2.130535
1	-0.961273	-2.048288	-2.007126
1	0.365220	-2.444774	-3.149347
6	0.736453	-3.037238	-1.101583
1	1.824012	-3.153216	-1.289687
1	0.624235	-2.605928	-0.090279
6	0.777320	0.033302	-3.104373
1	1.266161	0.962576	-2.793465
1	1.378865	-0.477090	-3.874175
1	-0.230165	0.258728	-3.488656
6	0.464713	-5.227322	-0.281956
1	0.283764	-4.892945	0.759344
1	-0.119719	-6.140617	-0.466094
1	1.542001	-5.468903	-0.383893
8	2.337800	1.048911	-0.542027
6	2.741369	0.190399	0.279263
6	4.181360	0.012477	0.592248
8	1.867879	-0.566580	0.855906
6	5.133228	0.831052	-0.036095
6	4.591705	-0.968556	1.508737
6	6.488650	0.666619	0.250221
1	4.788329	1.589228	-0.741728
6	5.948738	-1.129995	1.792015
1	3.834105	-1.590448	1.988857
6	6.896433	-0.313650	1.163324
1	7.231223	1.303571	-0.236700
1	6.270652	-1.892113	2.505996
1	7.958902	-0.440797	1.387332
6	-3.697156	-4.228477	0.517975
6	-2.848498	-3.126349	0.386266
6	-3.324638	-1.835852	0.666332
6	-4.655909	-1.661938	1.077072
6	-5.498352	-2.765221	1.216455
6	-5.019888	-4.051312	0.936881
1	-3.322741	-5.229720	0.289310
1	-1.822440	-3.267295	0.048207
1	-5.006402	-0.648287	1.281234
1	-6.532460	-2.624688	1.541631
1	-5.681095	-4.915649	1.042859
6	-2.456318	-0.617619	0.524571
8	-2.896770	0.510380	0.630178
8	-1.179928	-0.887451	0.267436
8	-0.584095	1.813548	-1.167634
6	-0.677688	2.461048	-0.075427
6	-1.333656	3.783313	-0.012911
6	-1.851258	4.362403	-1.181915
6	-1.443948	4.452168	1.216028
6	-2.473241	5.609425	-1.121065
1	-1.759019	3.819000	-2.123903
6	-2.067472	5.698835	1.272062
1	-1.040826	3.977284	2.112127
6	-2.580816	6.277268	0.104936
1	-2.878314	6.063190	-2.028902
1	-2.157304	6.221856	2.227270
1	-3.070064	7.253875	0.151355
8	-0.175515	1.923953	0.969112

Structure S53. **^{5}INT2-3**

E(B3LYP)$_{sol}$ = -3608.59757847 E(B3LYP) = -3605.98755661

26	-0.371644	-0.739921	0.368205
8	-0.391558	-2.742715	1.406802
8	-0.512522	-2.172849	-1.270152
6	-0.358449	-3.780913	0.425833
1	0.685760	-3.957389	0.114413
1	-0.779589	-4.711756	0.847857
6	-1.162405	-3.329565	-0.773336
1	-1.170955	-4.129095	-1.536754
1	-2.199981	-3.086793	-0.482294
6	0.529714	-2.956852	2.471833
1	0.418292	-2.120249	3.172697
1	0.301197	-3.902588	2.993890
1	1.564548	-2.965266	2.094304
6	-1.010564	-1.625823	-2.486378
1	-2.039111	-1.253725	-2.347816
1	-0.334914	-0.800010	-2.738919
1	-0.980099	-2.386188	-3.285624
8	3.570871	0.281372	-0.318183
16	2.684434	-0.829944	-0.668954
8	1.639736	-1.100174	0.410219
8	2.153409	-0.886575	-2.037662
6	3.701584	-2.389545	-0.481205
9	4.118655	-2.530731	0.780314
9	4.759819	-2.355708	-1.284318
9	2.960849	-3.463930	-0.795521
8	-2.311111	-0.562464	0.233954
6	-3.046760	0.507718	0.149469
6	-4.370866	0.316882	-0.556540
8	-2.740244	1.611761	0.591745
6	-4.791732	-0.942761	-1.008432
6	-5.198219	1.430962	-0.763867
6	-6.018877	-1.086883	-1.660953
1	-4.145896	-1.803871	-0.834104
6	-6.422880	1.289505	-1.418775
1	-4.853119	2.401037	-0.401016
6	-6.835761	0.029798	-1.868992
1	-6.341110	-2.072395	-2.007949
1	-7.060176	2.163111	-1.579902
1	-7.795259	-0.081589	-2.381292
6	-0.414152	1.382365	3.157285
6	-1.024957	2.779349	3.087068
1	-0.256445	3.542764	2.900652
1	-1.776904	2.805965	2.287263
1	-1.505868	3.008513	4.050251
6	-1.479092	0.335336	3.484541
1	-2.342883	0.457482	2.819899
1	-1.085918	-0.683639	3.361159
1	-1.794868	0.460744	4.530797
6	0.798450	1.299279	4.080310
1	1.207158	0.277428	4.090377
1	1.589752	1.982467	3.739413
1	0.513800	1.578852	5.106081
8	0.012604	0.952651	1.817958
8	0.849595	1.975626	1.240627
6	0.796353	1.929379	-0.103937
6	1.601033	2.996483	-0.727350
6	1.791131	4.243051	-0.110895
6	2.184484	2.714721	-1.973919
6	2.551467	5.218779	-0.755669
1	1.332946	4.445884	0.858632
6	2.951813	3.693018	-2.603644

1	2.061038	1.720271	-2.406679
6	3.130920	4.943330	-1.999669
1	2.693551	6.195839	-0.288070
1	3.420752	3.476874	-3.566246
1	3.731321	5.707790	-2.499703
8	0.190152	1.070147	-0.720599

Structure S54. **^{5}TS1-3**

E(B3LYP)$_{sol}$ = -3608.58892381 E(B3LYP) = -3605.97860910

26	-0.289646	-0.662698	0.347316
8	-0.346486	-2.728693	1.230907
8	-0.407915	-1.995700	-1.390233
6	-0.294118	-3.712128	0.192843
1	0.758002	-3.873082	-0.098638
1	-0.729109	-4.661552	0.553822
6	-1.061821	-3.189633	-1.000253
1	-1.029850	-3.934629	-1.816019
1	-2.112961	-2.976627	-0.737498
6	0.560196	-3.010607	2.296547
1	0.510202	-2.169413	2.997173
1	0.265588	-3.942199	2.809875
1	1.591108	-3.084782	1.917915
6	-0.851288	-1.385227	-2.597481
1	-1.906967	-1.078873	-2.508856
1	-0.206490	-0.510916	-2.744202
1	-0.722881	-2.083976	-3.441309
8	3.602546	0.341300	-0.251467
16	2.772057	-0.786229	-0.679313
8	1.702147	-1.146361	0.351040
8	2.295256	-0.810072	-2.067576
6	3.835084	-2.317171	-0.508995
9	4.227581	-2.481906	0.757146
9	4.909330	-2.228664	-1.286215
9	3.134621	-3.404593	-0.870208
8	-2.228407	-0.532741	0.101039
6	-2.938466	0.539493	-0.126859
6	-4.322906	0.275786	-0.671113
8	-2.557902	1.688727	0.069670
6	-4.820736	-1.027030	-0.823574
6	-5.128402	1.366177	-1.033449
6	-6.104598	-1.237457	-1.333016
1	-4.189974	-1.866942	-0.529662
6	-6.409576	1.156971	-1.546443
1	-4.721851	2.370989	-0.903387
6	-6.900156	-0.145618	-1.697324
1	-6.487784	-2.255164	-1.446219
1	-7.030260	2.010945	-1.830373
1	-7.904014	-0.309676	-2.098315
6	-0.683013	0.985700	3.159360
6	-1.429170	2.312538	3.018406
1	-0.723540	3.148264	2.919069
1	-2.075545	2.288510	2.131166
1	-2.037338	2.481975	3.919962
6	-1.678521	-0.160561	3.408153
1	-2.404518	-0.225864	2.587821
1	-1.167831	-1.128192	3.494923
1	-2.206091	0.036702	4.353715
6	0.414805	1.034624	4.218953
1	0.927200	0.063537	4.293890
1	1.160873	1.797652	3.956051

1	-0.014445	1.285515	5.201054
8	-0.072560	0.598904	1.897917
8	0.808077	1.928330	1.387238
6	0.818012	1.947746	0.078407
6	1.532162	3.119689	-0.484405
6	1.530669	4.366591	0.158254
6	2.225252	2.931575	-1.691559
6	2.206802	5.439209	-0.424127
1	0.989455	4.491379	1.097759
6	2.907871	4.007362	-2.257528
1	2.243402	1.939896	-2.146418
6	2.895175	5.258753	-1.629339
1	2.198262	6.417526	0.062146
1	3.458101	3.867901	-3.190959
1	3.429593	6.098957	-2.080360
8	0.337020	1.069769	-0.644107

Frequencies -- -586.1345

Red. masses -- 13.9230

Frc consts -- 2.8182

IR Inten -- 923.7972

Structure S55. ^{7}**INT3-3**

$E(B3LYP)_{sol}$ = -3608.61517137 E(B3LYP) = -3606.00719354

26	0.480430	-0.054616	0.152981
8	1.683482	1.579552	1.097333
8	0.784064	1.409863	-1.428675
6	1.780083	2.738787	0.255500
1	0.876373	3.354562	0.386752
1	2.676939	3.320038	0.533014
6	1.877651	2.287673	-1.186986
1	1.801177	3.171787	-1.842781
1	2.830273	1.772101	-1.397432
6	1.301702	1.894903	2.442107
1	1.181456	0.939591	2.965792
1	2.088982	2.501671	2.919382
1	0.339318	2.428653	2.443410
6	0.450825	1.151110	-2.800699
1	1.333980	0.778939	-3.339387
1	-0.324297	0.378582	-2.780599
1	0.044387	2.070422	-3.249280
8	-2.187670	1.827685	-1.398643
16	-1.854372	2.221314	-0.026345
8	-1.011219	1.184641	0.724693
8	-1.379372	3.588301	0.218657
6	-3.445503	2.049194	0.939628
9	-3.926996	0.814526	0.795937
9	-4.342096	2.919593	0.481978
9	-3.229549	2.285269	2.231600
8	2.098026	-0.843163	-0.399769
6	2.959323	-0.889055	-1.398470
6	4.375671	-0.565338	-1.005660
8	2.650339	-1.154533	-2.544939
6	4.672017	0.041039	0.225965
6	5.410725	-0.841265	-1.911917
6	5.993534	0.359738	0.548190
1	3.854402	0.279814	0.907505
6	6.731427	-0.533439	-1.582290
1	5.153383	-1.298354	-2.869579
6	7.024489	0.067047	-0.351622
1	6.221754	0.837054	1.504803

1	7.536668	-0.758761	-2.286176
1	8.058953	0.310779	-0.095428
6	0.820666	-2.323730	2.289200
6	0.819997	-3.409024	1.205945
1	-0.212854	-3.675452	0.944072
1	1.332407	-3.047014	0.304629
1	1.333302	-4.311317	1.571819
6	2.272337	-1.974826	2.693364
1	2.838432	-1.647886	1.812033
1	2.284053	-1.176979	3.451110
1	2.753113	-2.868037	3.121216
6	-0.003623	-2.740908	3.511307
1	0.012327	-1.946551	4.272699
1	-1.046347	-2.909156	3.209895
1	0.401150	-3.664974	3.952658
8	0.273242	-1.111281	1.801346
8	-1.790801	-1.744398	1.088916
6	-1.859302	-1.538902	-0.151436
6	-3.153396	-1.851410	-0.825471
6	-4.001472	-2.842655	-0.307822
6	-3.531212	-1.110122	-1.955657
6	-5.214397	-3.114569	-0.941019
1	-3.698221	-3.393987	0.584132
6	-4.752825	-1.378493	-2.574003
1	-2.882787	-0.307019	-2.305906
6	-5.590048	-2.382680	-2.073720
1	-5.871347	-3.894976	-0.549370
1	-5.056180	-0.797488	-3.448286
1	-6.543858	-2.591895	-2.565179
8	-0.905393	-1.075179	-0.854391

Structure S56. ^{7}**TS2-3**

E(B3LYP)$_{sol}$ = -3608.60284750 E(B3LYP) = -3605.98784042

26	0.406106	-0.127622	0.168813
8	1.525279	1.816754	0.709590
8	0.596744	0.970545	-1.644529
6	1.529083	2.749934	-0.373796
1	0.606730	3.350538	-0.340446
1	2.403130	3.421173	-0.288622
6	1.603417	1.979158	-1.675544
1	1.409160	2.670052	-2.512777
1	2.590244	1.510688	-1.826108
6	1.222223	2.419203	1.972023
1	1.159352	1.602324	2.699751
1	2.025900	3.122045	2.252024
1	0.254150	2.940093	1.924203
6	0.179079	0.448840	-2.915372
1	1.059492	0.162952	-3.507709
1	-0.409132	-0.448896	-2.699321
1	-0.446049	1.201957	-3.417735
8	-2.220138	1.924659	-1.460244
16	-1.991064	2.148914	-0.029083
8	-1.174926	1.043431	0.643698
8	-1.555889	3.488541	0.394461
6	-3.636945	1.877208	0.819160
9	-4.060724	0.632558	0.619973
9	-4.532013	2.727645	0.322483
9	-3.512030	2.090147	2.127278
8	2.193615	-0.776049	-0.131160
6	2.941975	-0.981655	-1.192849

6	4.377850	-0.559675	-1.013418
8	2.538326	-1.444022	-2.245694
6	4.742015	0.358658	-0.015129
6	5.352596	-1.050615	-1.894205
6	6.071741	0.770095	0.106968
1	3.963835	0.761989	0.636016
6	6.683560	-0.651532	-1.759872
1	5.041383	-1.744157	-2.678183
6	7.044945	0.258709	-0.758961
1	6.351557	1.492739	0.878174
1	7.443136	-1.045790	-2.439849
1	8.086573	0.574996	-0.658402
6	0.916973	-1.717987	2.734371
6	1.211748	-2.975406	1.953089
1	0.219206	-3.391613	1.709016
1	1.721556	-2.745187	1.010764
1	1.784409	-3.700554	2.546289
6	2.866815	-1.087846	3.065575
1	3.145981	-0.945200	2.019625
1	2.707728	-0.186039	3.661192
1	3.281036	-1.952001	3.591194
6	0.422104	-1.891220	4.154825
1	0.383217	-0.927248	4.679455
1	-0.609943	-2.272394	4.059112
1	1.015488	-2.619728	4.723455
8	0.504757	-0.664157	2.140852
8	-1.768846	-2.629067	1.228450
6	-1.792666	-2.093295	0.131344
6	-3.023382	-2.095185	-0.719951
6	-4.032185	-3.031277	-0.442483
6	-3.208313	-1.154401	-1.742224
6	-5.203615	-3.042368	-1.199443
1	-3.877304	-3.739532	0.373757
6	-4.390525	-1.154180	-2.484813
1	-2.456284	-0.387626	-1.913852
6	-5.384196	-2.102697	-2.222320
1	-5.983114	-3.779117	-0.988721
1	-4.538805	-0.401636	-3.263164
1	-6.307025	-2.104848	-2.808715
8	-0.732699	-1.502185	-0.402136

Frequencies -- -335.5835

Red. masses -- 7.4410

Frc consts -- 0.4937

IR Inten -- 22.7794

Structure S57. **[6]INT4-3**

E(B3LYP)$_{sol}$ = -3375.47667353 E(B3LYP) = -3373.12274970 a.u. after 2 cycles

----

26	0.021103	-0.367262	0.140155
8	-0.152373	-2.623487	-0.223179
8	-0.210601	-1.219621	2.059896
6	-0.391044	-3.354124	0.979577
1	-1.476381	-3.435463	1.150603
1	0.052709	-4.364049	0.904298
6	0.275924	-2.568190	2.091298
1	0.080745	-3.021143	3.075376
1	1.360027	-2.502789	1.918949
6	-0.857577	-3.107812	-1.364840
1	-0.601277	-2.447326	-2.203332
1	-0.532361	-4.136503	-1.596595
1	-1.943608	-3.077725	-1.195209

6	-1.224850	-0.843028	3.004131
1	-0.797950	-0.849916	4.019409
1	-1.537507	0.170358	2.728653
1	-2.088660	-1.519653	2.929968
8	-3.659012	0.343855	1.123119
16	-3.220605	-0.736415	0.245579
8	-1.876244	-0.443229	-0.436421
8	-3.291866	-2.120353	0.744167
6	-4.328633	-0.701204	-1.260983
9	-4.277245	0.487805	-1.847245
9	-5.579265	-0.957390	-0.891413
9	-3.933699	-1.634612	-2.131726
8	1.842849	-0.787224	0.027459
6	2.941447	-0.042048	0.044456
6	4.188445	-0.814276	-0.282142
8	2.956009	1.144810	0.306115
6	4.135149	-2.171216	-0.637666
6	5.426652	-0.156293	-0.232072
6	5.310980	-2.862809	-0.937683
1	3.162043	-2.664150	-0.679198
6	6.600537	-0.849485	-0.530092
1	5.441306	0.900272	0.043022
6	6.544139	-2.203439	-0.882893
1	5.267742	-3.918775	-1.217245
1	7.564001	-0.334891	-0.489170
1	7.464183	-2.745501	-1.117464
8	0.223898	1.336688	-1.016538
6	0.126214	2.046017	0.035039
6	0.248408	3.514851	-0.005155
6	0.520854	4.157984	-1.222747
6	0.096562	4.262646	1.172919
6	0.638287	5.547146	-1.259675
1	0.640667	3.553671	-2.123621
6	0.213494	5.651838	1.130166
1	-0.110353	3.739239	2.107884
6	0.483909	6.293154	-0.084746
1	0.852037	6.052261	-2.204697
1	0.095428	6.238399	2.044437
1	0.576216	7.382049	-0.116004
8	-0.091350	1.425134	1.132726

Structure S58. **[5]INT2-4**

E(B3LYP)$_{sol}$ = -2646.63999411 E(B3LYP) = -2644.87238908

26	-0.223122	-0.988656	0.053659
8	0.227956	-2.791296	1.123227
8	-0.670334	-2.519397	-1.392377
6	0.012874	-3.972408	0.341879
1	0.949314	-4.239938	-0.180192
1	-0.286308	-4.809326	0.996122
6	-1.082640	-3.671830	-0.656706
1	-1.219483	-4.534220	-1.331675
1	-2.036725	-3.453935	-0.143550
6	1.242225	-2.916265	2.120842
1	1.329916	-1.939307	2.609587
1	0.960310	-3.684242	2.859608
1	2.208505	-3.183956	1.659454
6	-1.519936	-2.169228	-2.490797
1	-1.148592	-1.215983	-2.885042
1	-1.476412	-2.950653	-3.266504
1	-2.558185	-2.037613	-2.144389

6	-5.951819	1.207827	-0.251428
6	-4.747682	0.543859	-0.017235
6	-3.567995	0.997279	-0.632092
6	-3.601703	2.115735	-1.482708
6	-4.808113	2.776241	-1.713180
6	-5.981860	2.322779	-1.098160
1	-6.870572	0.858456	0.225343
1	-4.701209	-0.326828	0.639195
1	-2.674812	2.449678	-1.952608
1	-4.837815	3.645956	-2.373611
1	-6.926382	2.841540	-1.280460
6	-2.287805	0.296819	-0.390792
8	-1.206620	0.685622	-0.924568
8	-2.245147	-0.731022	0.383497
6	0.371653	1.306193	2.549415
6	-0.082882	2.526222	1.757846
1	0.778371	3.068919	1.342958
1	-0.738921	2.230186	0.928188
1	-0.630136	3.210955	2.422098
6	-0.796358	0.516091	3.138639
1	-1.530119	0.230545	2.372239
1	-0.442847	-0.390636	3.651834
1	-1.303390	1.150117	3.880575
6	1.421867	1.632403	3.605241
1	1.771873	0.718458	4.107834
1	2.285423	2.151341	3.167330
1	0.976822	2.294417	4.362559
8	0.945485	0.306886	1.602487
8	2.120120	0.886751	0.992162
6	2.438220	0.248004	-0.144696
6	3.671640	0.747091	-0.763767
6	4.472709	1.729867	-0.148599
6	4.038091	0.211374	-2.013513
6	5.630400	2.169475	-0.786122
1	4.190820	2.141671	0.821368
6	5.197540	0.658764	-2.642166
1	3.404470	-0.547444	-2.475550
6	5.992147	1.636025	-2.029947
1	6.255691	2.929954	-0.313830
1	5.484563	0.247649	-3.612252
1	6.901224	1.985188	-2.525572
8	1.743833	-0.663772	-0.592918

Structure S59. **[5]TS1-4**

E(B3LYP)$_{sol}$ = -2646.63228695 E(B3LYP) = -2644.86512730

26	0.154625	0.793763	0.055394
8	-0.286520	2.649342	1.060958
8	0.668439	2.342553	-1.392562
6	0.016695	3.831526	0.307488
1	-0.889374	4.155031	-0.235080
1	0.338387	4.637799	0.988442
6	1.120113	3.485610	-0.665455
1	1.295232	4.334858	-1.347630
1	2.056552	3.233499	-0.138420
6	-1.365563	2.808982	1.986820
1	-1.513184	1.840062	2.475331
1	-1.110129	3.575022	2.736516
1	-2.288043	3.100604	1.456442
6	1.463671	1.996708	-2.531669
1	1.073434	1.046425	-2.914052

1	1.380108	2.782777	-3.299287
1	2.518919	1.866380	-2.240671
6	6.051158	-1.039023	-0.312557
6	4.835827	-0.410958	-0.043186
6	3.650701	-0.908624	-0.612869
6	3.689523	-2.035487	-1.452716
6	4.907988	-2.659781	-1.718001
6	6.086848	-2.162123	-1.148822
1	6.974275	-0.656168	0.128554
1	4.784113	0.464593	0.606545
1	2.758836	-2.405728	-1.886277
1	4.942781	-3.535960	-2.369441
1	7.040515	-2.653053	-1.358381
6	2.364380	-0.242400	-0.329748
8	1.271482	-0.682413	-0.840115
8	2.290497	0.777293	0.426318
6	-0.275384	-1.152775	2.573652
6	0.213622	-2.448905	1.938277
1	-0.625904	-2.997013	1.488190
1	0.951693	-2.241617	1.151519
1	0.675201	-3.088618	2.704523
6	0.874729	-0.330144	3.179801
1	1.665873	-0.124833	2.447681
1	0.507873	0.624596	3.583578
1	1.302134	-0.914147	4.008975
6	-1.386919	-1.366314	3.597105
1	-1.765140	-0.403438	3.971831
1	-2.222767	-1.933615	3.167999
1	-0.987953	-1.935115	4.449680
8	-0.757909	-0.252724	1.517447
8	-2.118258	-0.974011	0.895038
6	-2.454290	-0.346618	-0.206990
6	-3.757930	-0.755420	-0.767580
6	-4.640933	-1.589460	-0.055415
6	-4.099493	-0.287152	-2.050136
6	-5.859798	-1.947806	-0.627613
1	-4.373094	-1.946540	0.940149
6	-5.320077	-0.653082	-2.613549
1	-3.401112	0.356321	-2.587413
6	-6.198460	-1.481749	-1.904217
1	-6.550303	-2.591434	-0.078538
1	-5.589682	-0.293241	-3.608829
1	-7.154872	-1.766454	-2.349440
8	-1.731863	0.515103	-0.740638

Frequencies -- -555.6746

Red. masses -- 13.4923

Frc consts -- 2.4546

IR Inten -- 1368.4080

Structure S60. **[7]INT3-4**

E(B3LYP)$_{sol}$ = -2646.65442020 E(B3LYP) = -2644.88361151

26	-0.057226	-0.681486	0.327401
8	0.301705	-2.313864	1.636616
8	-0.365648	-2.462992	-0.901127
6	0.199718	-3.623479	1.061245
1	1.190094	-3.925211	0.677961
1	-0.131136	-4.343343	1.828299
6	-0.801753	-3.539456	-0.066598
1	-0.811628	-4.485439	-0.632779
1	-1.816445	-3.319911	0.307918

6	1.291642	-2.191699	2.672042
1	1.268699	-1.148202	3.002712
1	1.039749	-2.867296	3.503964
1	2.288715	-2.435775	2.270285
6	-0.970622	-2.405187	-2.198904
1	-0.577750	-1.509282	-2.691412
1	-0.703753	-3.308168	-2.770395
1	-2.066860	-2.326132	-2.110601
6	-5.941054	0.664804	-0.784232
6	-4.720932	0.172920	-0.324763
6	-3.528234	0.565694	-0.959979
6	-3.562690	1.451894	-2.052694
6	-4.786973	1.940279	-2.505393
6	-5.973274	1.546774	-1.872526
1	-6.870681	0.364636	-0.295784
1	-4.672331	-0.512745	0.523141
1	-2.626072	1.748156	-2.528469
1	-4.821083	2.630110	-3.351459
1	-6.931597	1.931965	-2.229667
6	-2.241589	0.052814	-0.475752
8	-1.134357	0.379202	-1.040129
8	-2.151428	-0.729964	0.529511
6	-0.243167	1.688374	2.422461
6	-0.785185	2.726724	1.435794
1	0.020089	3.060866	0.767759
1	-1.595224	2.308974	0.822660
1	-1.181465	3.593856	1.984397
6	-1.382543	1.076708	3.269683
1	-2.129720	0.598356	2.621970
1	-0.986122	0.327130	3.970489
1	-1.863831	1.878614	3.849880
6	0.856528	2.270003	3.314489
1	1.254068	1.497959	3.990621
1	1.679268	2.652320	2.695702
1	0.454908	3.092762	3.924480
8	0.313938	0.590042	1.709567
8	1.974654	1.482970	0.464023
6	2.421739	0.522955	-0.220333
6	3.755373	0.674320	-0.853078
6	4.610120	1.722005	-0.462500
6	4.152139	-0.230084	-1.854862
6	5.858750	1.855057	-1.066807
1	4.289132	2.416754	0.315403
6	5.402147	-0.088707	-2.454521
1	3.474485	-1.033324	-2.147740
6	6.253994	0.952195	-2.061882
1	6.528286	2.662555	-0.762736
1	5.717178	-0.790060	-3.230418
1	7.233328	1.059573	-2.534645
8	1.767482	-0.561709	-0.404623

Structure S61. ^{7}**TS2-4**
E(B3LYP)$_{sol}$ = -2646.64601915 E(B3LYP) = -2644.86955607

26	-0.010666	-0.081915	0.046116
8	1.103094	-0.615332	1.784115
8	0.228526	-2.240959	-0.105474
6	1.465790	-1.997108	1.892551
1	2.445021	-2.150974	1.407568
1	1.535070	-2.282324	2.955679
6	0.385692	-2.800364	1.205158

1	0.690896	-3.857200	1.130679
1	-0.574095	-2.725010	1.743413
6	2.011416	0.298242	2.422662
1	1.631699	1.305258	2.220772
1	2.022378	0.105073	3.506706
1	3.022943	0.183811	2.001746
6	-0.590352	-3.008619	-0.998771
1	-0.670172	-2.435696	-1.929311
1	-0.114055	-3.983335	-1.188084
1	-1.594195	-3.157057	-0.568300
6	-6.081442	-0.944079	0.744981
6	-4.700254	-0.838023	0.897455
6	-3.895148	-0.555287	-0.221337
6	-4.478678	-0.379776	-1.489793
6	-5.861179	-0.487099	-1.634313
6	-6.660401	-0.768857	-0.518944
1	-6.711880	-1.163191	1.609584
1	-4.227407	-0.968684	1.872644
1	-3.838046	-0.162939	-2.346561
1	-6.320071	-0.352957	-2.616344
1	-7.743756	-0.852830	-0.635475
6	-2.438333	-0.438099	-0.056069
8	-1.683578	-0.144895	-1.068155
8	-1.857373	-0.597947	1.055108
6	-0.395578	2.922935	0.180884
6	-0.537484	2.982488	-1.319970
1	0.496395	2.927154	-1.704085
1	-1.096399	2.120311	-1.705519
1	-0.993044	3.922941	-1.655296
6	-2.395763	3.120514	0.717277
1	-2.844284	2.329164	0.112947
1	-2.331996	2.935078	1.791345
1	-2.582074	4.148621	0.396763
6	0.195849	4.125543	0.876118
1	0.094828	4.046300	1.966806
1	1.270148	4.096377	0.618803
1	-0.226816	5.072770	0.517281
8	-0.173748	1.793633	0.749836
8	2.559184	2.218785	-0.665569
6	2.554627	1.013497	-0.830339
6	3.780981	0.165224	-0.773326
6	5.018335	0.779663	-0.520631
6	3.710464	-1.229803	-0.933252
6	6.174261	0.004339	-0.422908
1	5.051626	1.864762	-0.404791
6	4.869409	-2.001651	-0.834137
1	2.743377	-1.694128	-1.133302
6	6.100596	-1.385385	-0.577994
1	7.137145	0.482443	-0.228832
1	4.818504	-3.085327	-0.965322
1	7.007567	-1.990471	-0.504057
8	1.421539	0.334476	-1.037149

Frequencies -- -291.4152

Red. masses -- 7.1081

Frc consts -- 0.3557

IR Inten -- 19.1656

Structure S62. **[6]INT4-4**

E(B3LYP)$_{sol}$ = -2413.51547235 E(B3LYP) = -2411.99766166

26	0.031363	0.680780	-0.023403

8	0.465679	2.506505	-1.054751
8	-0.629758	2.234200	1.312325
6	0.206486	3.714419	-0.326529
1	1.108637	3.991941	0.246034
1	-0.046647	4.527790	-1.026461
6	-0.959030	3.432318	0.595420
1	-1.098639	4.267873	1.300414
1	-1.890774	3.268780	0.026357
6	1.433503	2.609010	-2.115320
1	1.552742	1.601903	-2.530079
1	1.061580	3.302913	-2.884673
1	2.396516	2.964155	-1.713884
6	-1.466030	1.924795	2.442116
1	-1.118290	0.963805	2.836943
1	-1.359067	2.715434	3.200382
1	-2.517825	1.837216	2.125095
6	-5.553335	-1.549839	-1.210390
6	-4.334377	-0.877017	-1.174665
6	-3.412069	-1.164301	-0.149852
6	-3.713379	-2.126924	0.833199
6	-4.935000	-2.794573	0.789257
6	-5.852339	-2.505859	-0.230130
1	-6.273800	-1.334778	-2.002443
1	-4.078285	-0.132300	-1.930442
1	-2.982013	-2.339812	1.615008
1	-5.176205	-3.543775	1.546329
1	-6.809174	-3.032887	-0.262305
6	-2.132031	-0.461153	-0.109128
8	-1.254788	-0.687767	0.806355
8	-1.802621	0.431613	-0.970007
8	1.476730	-0.333312	-0.999005
6	2.232294	-0.366120	0.055433
6	3.523629	-1.048036	0.052781
6	3.972559	-1.699381	-1.112573
6	4.309358	-1.052691	1.221709
6	5.204555	-2.349090	-1.104952
1	3.345935	-1.690614	-2.006197
6	5.540020	-1.704481	1.219416
1	3.939790	-0.546509	2.115334
6	5.986004	-2.350625	0.058361
1	5.559631	-2.858552	-2.003283
1	6.155498	-1.713984	2.121486
1	6.951639	-2.862361	0.060832
8	1.764557	0.240108	1.076224

Structure S63. **[6]INT5-1**

E(B3LYP)$_{sol}$ = -3098.76294899 E(B3LYP) = -3096.74661945

26	0.231840	-0.344598	0.164139
8	2.265366	0.083132	0.464575
6	2.141671	1.186191	-0.164777
6	3.293956	2.099988	-0.342568
8	0.995460	1.472342	-0.632970
6	4.548814	1.759676	0.186987
6	3.126707	3.306883	-1.040260
6	5.631196	2.623380	0.016811
1	4.654737	0.818205	0.728839
6	4.211921	4.167363	-1.207977
1	2.141093	3.551246	-1.440530
6	5.463105	3.825881	-0.680384
1	6.609120	2.361348	0.427981

1	4.084777	5.107304	-1.750516
1	6.312357	4.501408	-0.812827
6	-4.958495	2.558832	1.723159
6	-3.917179	1.814008	1.168597
6	-2.714227	1.663499	1.877118
6	-2.557127	2.258781	3.138908
6	-3.602238	3.000839	3.689613
6	-4.801468	3.150868	2.982293
1	-5.895747	2.679653	1.174499
1	-4.013175	1.343124	0.188406
1	-1.612043	2.126722	3.668967
1	-3.484539	3.464686	4.672001
1	-5.619055	3.733355	3.415310
6	-1.607261	0.873051	1.291766
8	-0.490098	0.754955	1.882184
8	-1.750986	0.284638	0.167389
8	-1.152327	-3.079877	-2.103179
6	-0.851757	-1.902383	-2.142984
6	-1.499717	-0.937267	-3.090802
6	-2.486520	-1.425637	-3.962728
6	-1.154856	0.423203	-3.121857
6	-3.123941	-0.564010	-4.855110
1	-2.738345	-2.486981	-3.919285
6	-1.796753	1.283005	-4.016041
1	-0.391962	0.809318	-2.446227
6	-2.779596	0.793046	-4.882333
1	-3.891940	-0.948369	-5.531324
1	-1.528175	2.342274	-4.034714
1	-3.279590	1.469683	-5.580746
8	0.090314	-1.389625	-1.354145
7	0.384135	-2.030085	1.564013
14	1.267938	-3.610051	1.128353
7	0.015640	-1.842752	2.727331
6	2.060837	-4.146401	2.746793
6	-0.042096	-4.794160	0.532247
6	2.527630	-3.138840	-0.161774
7	-0.341763	-1.640721	3.780416
1	2.589795	-5.102700	2.598703
1	2.796474	-3.405308	3.099312
1	1.316298	-4.302887	3.544550
1	-0.796889	-4.985584	1.312182
1	-0.537866	-4.375698	-0.359044
1	0.416913	-5.759182	0.257460
1	2.022221	-2.858299	-1.096785
1	3.125816	-2.278387	0.174168
1	3.197202	-3.993052	-0.356256

Structure S64. **[6]TS4-1**

E(B3LYP)$_{sol}$ = -3098.73697990 E(B3LYP) = -3096.72306534

26	-0.437927	-0.077826	-0.026855
8	0.148600	1.837330	0.646297
6	-0.274687	2.372860	-0.436428
6	-0.136596	3.830872	-0.661460
8	-0.802558	1.614963	-1.303408
6	0.460384	4.640542	0.318087
6	-0.603231	4.398725	-1.857709
6	0.590538	6.012385	0.099048
1	0.814434	4.178658	1.241515
6	-0.471407	5.770983	-2.071795
1	-1.064629	3.748503	-2.603264

6	0.125155	6.577088	-1.094606
1	1.054721	6.645104	0.859467
1	-0.833957	6.215908	-3.001680
1	0.227587	7.652225	-1.264193
6	-5.699745	-2.813289	-1.644824
6	-4.403033	-2.331014	-1.464529
6	-4.131986	-1.433962	-0.419030
6	-5.160382	-1.022763	0.444025
6	-6.454939	-1.508674	0.259975
6	-6.724406	-2.402786	-0.783444
1	-5.914855	-3.510997	-2.457849
1	-3.587140	-2.635577	-2.122471
1	-4.923325	-0.325870	1.250015
1	-7.257574	-1.191794	0.930322
1	-7.739672	-2.782415	-0.925997
6	-2.758126	-0.919308	-0.219640
8	-2.486592	-0.090578	0.699730
8	-1.798946	-1.307314	-0.977057
8	2.595856	-1.157547	0.833862
6	2.305239	-1.133253	-0.396353
6	3.236012	-1.809963	-1.341982
6	4.416750	-2.406237	-0.869355
6	2.929347	-1.850791	-2.712095
6	5.282546	-3.038412	-1.761669
1	4.640435	-2.365274	0.197739
6	3.798003	-2.485130	-3.600627
1	2.007857	-1.381835	-3.060931
6	4.973861	-3.078425	-3.126773
1	6.201215	-3.501997	-1.394154
1	3.558862	-2.517850	-4.666270
1	5.653498	-3.574625	-3.824540
8	1.275284	-0.568570	-0.877980
7	0.159925	-1.066518	1.655558
14	2.064724	-0.298032	2.501495
7	-0.605120	-1.630716	2.420611
6	0.901154	0.723091	3.615253
6	2.673124	-1.748199	3.528330
6	3.330943	1.041615	2.050794
7	-1.292287	-2.176421	3.146425
1	1.528047	1.224015	4.373749
1	0.387457	1.497022	3.026410
1	0.144161	0.124808	4.145326
1	1.854130	-2.457676	3.730404
1	3.488937	-2.295245	3.034770
1	3.029685	-1.374764	4.502595
1	4.262028	0.636267	1.628940
1	2.865903	1.700939	1.297537
1	3.564295	1.661985	2.930985

Frequencies -- -151.9294

Red. masses -- 7.4674

Frc consts -- 0.1016

IR Inten -- 39.7049

Structure S65. **^{6}INT6-1**

E(B3LYP)$_{sol}$ = -3098.78226297 E(B3LYP) = -3096.76479512

26	0.541163	-0.253830	0.793605
8	-1.051864	-1.284708	1.522200
6	-1.247356	-1.947777	0.435736
6	-2.439752	-2.829518	0.323794
8	-0.454996	-1.783513	-0.522702

6	-3.330540	-2.956752	1.400798
6	-2.683565	-3.517676	-0.874889
6	-4.463940	-3.762015	1.274711
1	-3.118497	-2.415118	2.324213
6	-3.817395	-4.321836	-0.997477
1	-1.973680	-3.404553	-1.696353
6	-4.708278	-4.443058	0.076179
1	-5.158725	-3.861957	2.112378
1	-4.008941	-4.857602	-1.930571
1	-5.596165	-5.073531	-0.021074
6	5.814737	-1.837630	-1.960353
6	4.611582	-1.273535	-1.534399
6	3.959008	-1.789831	-0.404050
6	4.513881	-2.872425	0.296172
6	5.717540	-3.433155	-0.132768
6	6.367870	-2.916286	-1.259768
1	6.323980	-1.438223	-2.840982
1	4.158448	-0.434465	-2.065521
1	3.986576	-3.257983	1.170685
1	6.151623	-4.275851	0.411005
1	7.310613	-3.357218	-1.594583
6	2.679822	-1.191148	0.052027
8	2.062890	-1.658302	1.059342
8	2.169450	-0.194566	-0.553775
8	-2.141397	2.549846	-0.857118
6	-0.907873	2.221877	-0.563374
6	0.067411	3.330211	-0.480409
6	-0.375701	4.662858	-0.406934
6	1.443568	3.040476	-0.443475
6	0.550534	5.695615	-0.269883
1	-1.445587	4.872506	-0.444846
6	2.362511	4.080411	-0.308859
1	1.787084	2.008684	-0.526010
6	1.918835	5.404358	-0.216007
1	0.207009	6.730288	-0.199035
1	3.430790	3.855723	-0.273452
1	2.643272	6.214729	-0.101257
8	-0.607214	1.036497	-0.362944
7	0.984060	1.026139	2.087847
14	-3.476561	1.390754	-0.898908
7	1.417706	1.969341	2.682699
6	-3.711798	0.781738	0.851551
6	-4.886671	2.481190	-1.470563
6	-3.057387	0.058086	-2.139134
7	1.830974	2.876422	3.254524
1	-4.549063	0.065004	0.892368
1	-2.813346	0.268385	1.224457
1	-3.947579	1.620489	1.526470
1	-5.058983	3.310994	-0.766449
1	-4.678927	2.909813	-2.464170
1	-5.818992	1.896623	-1.541247
1	-2.878022	0.494779	-3.135077
1	-2.161070	-0.500503	-1.832594
1	-3.896609	-0.652408	-2.224629

Structure S66. ^{7}**INT7-1**

E(B3LYP)$_{sol}$ = -4344.58956961 E(B3LYP) = -4341.50840659

26	-1.751005	0.490740	-0.567206
8	-3.450176	-0.238481	-1.469118
6	-4.167267	-0.084886	-0.415088

6	-5.572316	-0.564759	-0.401383	6	2.936436	-2.300805	2.618325
8	-3.631839	0.427856	0.602627	1	1.517338	-3.688859	1.752059
6	-6.116690	-1.184722	-1.536811	6	2.256252	0.029440	2.630390
6	-6.346740	-0.416351	0.759788	1	0.339095	0.467264	1.731697
6	-7.428935	-1.659641	-1.506732	6	3.191019	-0.962296	2.944623
1	-5.496262	-1.285760	-2.429120	1	3.682304	-3.067731	2.837219
6	-7.658378	-0.890974	0.785330	1	2.460829	1.075100	2.868216
1	-5.899440	0.069023	1.629247	1	4.134536	-0.692089	3.423936
6	-8.198973	-1.513594	-0.346672	8	-1.281660	-1.160819	0.609178
1	-7.855023	-2.143466	-2.389133	7	-0.425029	-0.110569	-1.825146
1	-8.263194	-0.777067	1.688344	14	-2.318669	-3.914634	0.381730
1	-9.226739	-1.885668	-0.324833	7	0.185875	-1.139590	-1.919226
6	-0.580027	5.883111	2.153613	6	-2.407120	-3.471918	-1.428863
6	-0.727459	4.549210	1.773511	6	-2.087575	-5.750330	0.658573
6	-1.299475	4.236405	0.531442	6	-3.692548	-3.189565	1.423934
6	-1.731438	5.260148	-0.324582	7	0.786277	-2.117321	-2.012630
6	-1.575872	6.593845	0.055569	1	-3.192129	-4.073292	-1.917755
6	-0.998893	6.905465	1.292952	1	-2.650926	-2.409692	-1.576116
1	-0.133325	6.130304	3.119783	1	-1.448967	-3.688211	-1.927561
1	-0.394462	3.734774	2.418642	1	-1.226490	-6.129736	0.085235
1	-2.174256	4.990394	-1.285053	1	-1.918506	-5.973326	1.724202
1	-1.904318	7.394187	-0.611973	1	-2.983383	-6.304313	0.332263
1	-0.877160	7.950747	1.589033	1	-3.624324	-3.540396	2.466304
6	-1.414250	2.821898	0.108488	1	-3.644245	-2.090622	1.421277
8	-1.986412	2.505541	-0.985368	1	-4.677229	-3.486099	1.026247
8	-0.952934	1.879942	0.825147	6	1.335199	3.766221	-1.659982
8	-0.803307	-3.279273	1.035540	6	1.687981	2.477712	-2.033316
6	-0.497889	-2.008817	1.063384	6	2.551129	1.681348	-1.220793
6	0.793374	-1.653262	1.691388	6	3.035809	2.266858	-0.012722
6	1.735823	-2.650389	2.004111	6	2.675141	3.559138	0.349623
6	1.061163	-0.306813	1.992149	6	1.824140	4.319940	-0.465924

1	0.656142	4.346028	-2.289415
1	1.280609	2.043154	-2.948699
1	3.723242	1.702413	0.617822
1	3.061986	3.987912	1.278124
1	1.529594	5.328049	-0.167470
6	2.878980	0.364981	-1.626394
1	2.558815	0.040934	-2.618833
6	3.599578	-0.640875	-0.787684
1	3.426622	-0.473592	0.283264
1	3.239187	-1.653040	-1.022716
6	5.842223	-1.646908	-0.077613
9	5.675421	-1.251041	1.198726
9	5.287112	-2.863192	-0.200521
6	5.107942	-0.646082	-1.012079
9	5.638417	0.581018	-0.770992
9	5.396354	-0.977618	-2.293413
17	7.585326	-1.752728	-0.430215

Structure S67. **[7,5]MECP-1**

E(B3LYP)$_{sol}$ = -4344.58927961; -4344.58922683

26	-1.805164	0.492978	-0.537301
8	-3.500200	-0.252508	-1.434635
6	-4.209471	-0.119161	-0.372977
6	-5.608710	-0.613083	-0.352897
8	-3.671225	0.388441	0.646870
6	-6.156198	-1.223903	-1.491772
6	-6.374368	-0.487009	0.816469
6	-7.463447	-1.711756	-1.456897
1	-5.542005	-1.307221	-2.390178
6	-7.681023	-0.974554	0.846718
1	-5.924083	-0.008321	1.688176
6	-8.225027	-1.587858	-0.288726
1	-7.892185	-2.188322	-2.341909
1	-8.279223	-0.878018	1.756069
1	-9.248924	-1.970048	-0.263186
6	-0.590560	5.901993	2.137185
6	-0.755120	4.565686	1.772527
6	-1.345392	4.245613	0.540740
6	-1.778074	5.264857	-0.320384
6	-1.605588	6.600819	0.044341
6	-1.010472	6.919496	1.271254
1	-0.130159	6.154906	3.095454
1	-0.423298	3.754498	2.422467
1	-2.234677	4.989444	-1.272746
1	-1.934507	7.397516	-0.627368
1	-0.874820	7.966489	1.554897
6	-1.475193	2.828504	0.131645
8	-2.064100	2.507949	-0.951760
8	-1.005263	1.890456	0.848312
8	-0.758145	-3.273471	0.995322
6	-0.479383	-1.998147	1.051674
6	0.804262	-1.628380	1.685703
6	1.757708	-2.615553	1.997879
6	1.055887	-0.279878	1.990419
6	2.952488	-2.253870	2.616185
1	1.551665	-3.655576	1.742322
6	2.246157	0.068458	2.631125
1	0.325068	0.486574	1.730550
6	3.191496	-0.913281	2.945396
1	3.706573	-3.012812	2.834920

1	2.438451	1.115584	2.872543
1	4.131013	-0.633357	3.426802
8	-1.281716	-1.156463	0.615715
7	-0.473323	-0.059499	-1.813061
14	-2.249716	-3.925908	0.305471
7	0.190189	-1.055494	-1.898839
6	-2.320567	-3.436927	-1.494215
6	-1.979214	-5.762509	0.536922
6	-3.654848	-3.261727	1.346517
7	0.852759	-1.992324	-1.982105
1	-3.072558	-4.052732	-2.015761
1	-2.600656	-2.380639	-1.617745
1	-1.345366	-3.605502	-1.978177
1	-1.102170	-6.106535	-0.034561
1	-1.818588	-6.009819	1.598499
1	-2.857524	-6.328220	0.184279
1	-3.598308	-3.646719	2.377458
1	-3.626674	-2.162735	1.382382
1	-4.626921	-3.561801	0.921464
6	1.281968	3.659325	-1.775613
6	1.686518	2.381781	-2.134186
6	2.544431	1.612872	-1.289084
6	2.964862	2.212420	-0.064157
6	2.556787	3.494072	0.280729
6	1.714905	4.229617	-0.567632
1	0.610929	4.219909	-2.431049
1	1.327545	1.937269	-3.065246
1	3.644202	1.667280	0.591251
1	2.899427	3.935462	1.220376
1	1.386841	5.231430	-0.283928
6	2.937018	0.309722	-1.680096
1	2.655758	-0.032110	-2.676420
6	3.651121	-0.671064	-0.806803
1	3.436266	-0.503875	0.256878
1	3.326452	-1.694425	-1.043783
6	5.905064	-1.594265	-0.027109
9	5.692889	-1.190013	1.239814
9	5.393047	-2.829605	-0.149401
6	5.162188	-0.629477	-0.989601
9	5.643205	0.618045	-0.750232
9	5.491922	-0.964339	-2.260500
17	7.658219	-1.644712	-0.336876

Structure S68. ^{7}**TS5-1**
E(B3LYP)$_{sol}$ = -4344.55056777 E(B3LYP) = -4341.47105958

26	-1.848387	0.531241	-0.269263
8	-3.474202	-0.184793	-1.319184
6	-4.258563	-0.056379	-0.312093
6	-5.661887	-0.535508	-0.401115
8	-3.794055	0.434928	0.750804
6	-6.133189	-1.120093	-1.586794
6	-6.508237	-0.421420	0.712582
6	-7.444776	-1.592992	-1.654579
1	-5.457222	-1.194722	-2.440563
6	-7.819027	-0.894016	0.640480
1	-6.116453	0.036292	1.622895
6	-8.286985	-1.480668	-0.541821
1	-7.814188	-2.048789	-2.576624
1	-8.479733	-0.806687	1.506581
1	-9.314150	-1.851080	-0.596562

6	-0.396380	5.979513	2.221703	6	-2.196926	-3.276270	-1.575995
6	-0.621983	4.638162	1.909558	6	-1.971384	-5.748657	0.293551
6	-1.270774	4.298409	0.712819	6	-3.782200	-3.364346	1.142730
6	-1.701175	5.304842	-0.165285	7	1.382368	-1.488956	-1.778120
6	-1.474504	6.645103	0.150351	1	-2.871378	-3.871203	-2.214706
6	-0.820440	6.982531	1.341673	1	-2.503416	-2.222411	-1.642676
1	0.110759	6.246628	3.152088	1	-1.173800	-3.371823	-1.973734
1	-0.300921	3.837846	2.578677	1	-1.042814	-6.030531	-0.228387
1	-2.202324	5.014289	-1.090316	1	-1.889874	-6.073522	1.343264
1	-1.806293	7.430748	-0.532887	1	-2.803562	-6.303642	-0.170578
1	-0.641504	8.032869	1.586218	1	-3.800112	-3.829612	2.141531
6	-1.484262	2.873110	0.363600	1	-3.794268	-2.272074	1.267853
8	-2.066389	2.546255	-0.719054	1	-4.703254	-3.657670	0.612187
8	-1.086053	1.940367	1.130011	6	1.435194	3.044449	-2.682977
8	-0.849627	-3.276712	1.017301	6	1.960800	1.765053	-2.827533
6	-0.616402	-2.003933	1.210454	6	2.441824	1.041751	-1.709243
6	0.685745	-1.659499	1.822076	6	2.399653	1.674080	-0.444813
6	1.672550	-2.644663	2.012918	6	1.870418	2.953307	-0.304408
6	0.931142	-0.330375	2.206446	6	1.375608	3.642908	-1.417637
6	2.896073	-2.299880	2.583230	1	1.056765	3.578399	-3.558184
1	1.470087	-3.669104	1.698314	1	1.984489	1.293734	-3.813972
6	2.154558	0.001407	2.791129	1	2.777393	1.156165	0.435087
1	0.173005	0.435099	2.039091	1	1.827629	3.417488	0.683478
6	3.135629	-0.977253	2.979097	1	0.947143	4.639938	-1.297056
1	3.673888	-3.056016	2.709872	6	2.882743	-0.338032	-1.886889
1	2.345708	1.034026	3.092208	1	3.154698	-0.591835	-2.916243
1	4.099646	-0.708814	3.416217	6	3.751263	-1.028004	-0.863281
8	-1.462769	-1.149054	0.907082	1	3.389689	-0.860679	0.159338
7	-0.337052	0.055562	-1.374274	1	3.723470	-2.112798	-1.037402
14	-2.272592	-3.904899	0.179168	6	6.103899	-1.384536	0.068738
7	0.344559	-0.947929	-1.551658	9	5.666397	-1.151765	1.320782

9	5.981381	-2.700731	-0.165262
6	5.208993	-0.599478	-0.930914
9	5.350844	0.718958	-0.646487
9	5.705461	-0.810606	-2.177017
17	7.818362	-0.918666	-0.047325

Frequencies -- -611.6531

Red. masses -- 12.7547

Frc consts -- 2.8115

IR Inten -- 202.2280

Structure S69. ^{5}**INT9-1**

E(B3LYP)$_{sol}$ = -3514.74870377 E(B3LYP) = -3512.37340880

26	0.870473	-0.319891	-0.930608
8	-0.436880	1.092617	-1.575833
6	0.172397	2.059210	-0.974923
6	-0.387193	3.431656	-1.033372
8	1.208539	1.790611	-0.320960
6	-1.563260	3.688762	-1.754388
6	0.250194	4.466714	-0.331459
6	-2.101386	4.976826	-1.768849
1	-2.040841	2.868992	-2.294058
6	-0.290467	5.752317	-0.348507
1	1.160507	4.237191	0.225309
6	-1.466364	6.007383	-1.065757
1	-3.016942	5.180104	-2.329708
1	0.202871	6.560088	0.197755
1	-1.889097	7.015493	-1.077764
6	6.145399	-1.727039	1.900978
6	4.831136	-1.504467	1.488621
6	4.577331	-0.997314	0.204598
6	5.643330	-0.715655	-0.664322
6	6.956073	-0.938965	-0.247808
6	7.207310	-1.444067	1.033562
1	6.345186	-2.123285	2.899615
1	3.988049	-1.721045	2.146973
1	5.421499	-0.323614	-1.658555
1	7.787335	-0.720340	-0.922538
1	8.236550	-1.619376	1.357701
6	3.182889	-0.759037	-0.241166
8	2.928994	-0.327815	-1.404575
8	2.198100	-0.986101	0.541916
7	0.498455	-1.862341	-1.962102
7	1.087520	-2.731049	-2.556457
7	1.616860	-3.570137	-3.129162
6	-0.759205	-0.857521	0.709045
6	-0.702691	0.286387	1.574701
6	-1.952102	-1.175527	-0.150480
6	0.337324	0.383638	2.541490
6	-1.622943	1.365676	1.466763
1	-2.329621	-0.286274	-0.669681
1	-1.666940	-1.903048	-0.920720
6	-3.090026	-1.794999	0.654900
6	0.447085	1.503564	3.354118
1	1.069807	-0.421300	2.604058
6	-1.501588	2.482405	2.280314
1	-2.427646	1.323449	0.734295
6	-4.290536	-2.177705	-0.254468
9	-3.548439	-0.936241	1.600055
9	-2.664964	-2.915232	1.287481
6	-0.467334	2.558744	3.225885

1	1.255036	1.566342	4.086625
1	-2.208129	3.308458	2.174797
9	-4.724309	-1.070001	-0.878768
9	-3.871856	-3.042856	-1.189098
17	-5.626912	-2.900624	0.671543
1	-0.372449	3.444728	3.858608
1	-0.145964	-1.715549	1.002645

Structure S70. OBz⁻
E(B3LYP)sol = -420.521421077 E(B3LYP) = -419.963543249

6	1.836738	-1.210267	0.000031
6	0.437719	-1.205274	0.000006
6	-0.279186	0.000000	-0.000007
6	0.437718	1.205274	0.000006
6	1.836738	1.210267	0.000030
6	2.543303	0.000000	0.000043
1	2.384598	-2.159764	0.000040
1	-0.153780	-2.124899	-0.000005
1	-0.153781	2.124898	-0.000005
1	2.384596	2.159764	0.000040
1	3.638771	0.000001	0.000063
6	-1.842262	0.000000	-0.000037
8	-2.370313	1.133409	-0.000035
8	-2.370314	-1.133408	-0.000036

Structure S71. OTf⁻
E(B3LYP)sol = -961.931056451 E(B3LYP) = -960.954703923

8	1.246707	-0.348805	1.404218
16	0.938155	-0.000110	0.000242
8	1.247333	1.390214	-0.399804
8	1.247258	-1.041662	-1.003809
6	-0.949013	0.000153	-0.000319
9	-1.453907	0.900185	0.867620
9	-1.454219	-1.201549	0.345297
9	-1.452628	0.301682	-1.213673
